# A miR-137-XIAP axis contributes to the sensitivity of TRAIL-induced cell death in glioblastoma

**DOI:** 10.3389/fonc.2022.870034

**Published:** 2022-07-28

**Authors:** Fenghao Geng, Fen Yang, Fang Liu, Jianhui Zhao, Rui Zhang, Shijie Hu, Jie Zhang, Xiao Zhang

**Affiliations:** ^1^ Department of Radiation Medicine, Ministry of Education Key Laboratory of Hazard Assessment and Control in Special Operational Environment, School of Public Health, Fourth Military Medical University, Xi’an, China; ^2^ State Key Laboratory of Cancer Biology, Department of Biochemistry and Molecular Biology, Fourth Military Medical University, Xi’an, China; ^3^ Department of Neurology, Air Force Medical Center, Fourth Military Medical University, Beijing, China; ^4^ Department of Dermatology, Xijing Hospital, Fourth Military Medical University, Xi’an, China; ^5^ Department of Neurosurgery, Xijing Hospital, Fourth Military Medical University, Xi’an, China; ^6^ Department of Immunology, Fourth Military Medical University, Xi’an, China; ^7^ Research Office of the Institute of Tropical Medicine, Hainan Hospital of Chinese People's Liberation Army (PLA) General Hospital, Sanya, China

**Keywords:** miRNA, glioblastoma, TRAIL, apoptosis, combination therapy

## Abstract

Glioblastoma (GBM) is the most lethal primary brain tumor in the central nervous system with limited therapeutic strategies to prolong the survival rate in clinic. TNF-related apoptosis-inducing ligand (TRAIL)-based strategy has been demonstrated to induce cell death in an extensive spectrum of tumor cells, including GBM, while a considerable proportion of malignant cells are resistant to TRAIL-induced apoptosis. MiR-137 is highly expressed in the brain, but significantly decreases with advanced progression of GBM. However, the functional link between miR-137 and TRAIL-induced apoptosis in GBM cells has not been established. Here, GBM cells were transfected with miR-137, and gene expression levels were examined by qRT-PCR and western blot. Apoptotic cells were measured by Annexin-V staining and TUNEL assay. Our data showed that miR-137 sensitizes GBM cells to the TRAIL-mediated apoptosis. Mechanistically, we identified that XIAP is a *bona fide* target of miR-137, which is essential for miR-137-regulated sensitivity of TRAIL-induced cell death in GBM cells. Finally, in a xenograft model, combined utilization of miR-137 and TRAIL potently suppresses tumor growth *in vivo*. Collectively, we demonstrate that a miR-137-XIAP axis is required for the sensitivity of TRAIL-induced cell death and shed a light on the avenue for the treatment of GBM.

## Introduction

Glioblastoma (GBM) is the most lethal primary brain tumor in the central nervous system. Despite recent advances in multidisciplinary therapeutics for glioblastoma incorporating surgery, radiotherapy and chemotherapy, the median survival time is still no more than one and a half years. Therefore, it is urgent to explore the novel strategies for the treatment of GBM (1).

One of potential therapeutic approaches in cancer treatment is to reactivate apoptosis of transformed cells using member of TNF (tumor necrosis factor)-family, of which the TNF-related apoptosis-inducing ligand (TRAIL) gained much attention due to its therapeutic potential as a tumor-specific apoptosis inducer with no obvious cytotoxicity on normal cells (2). Although the TRAIL-based strategy has been demonstrated to induce cell death in an extensive spectrum of cancer cells including brain tumor cells, a considerable proportion of malignant cells are still resistant to TRAIL-induced apoptosis. More importantly, data from clinical trials showed that TRAIL application has only suboptimal benefit in the clinic because of complex mechanisms of TRAIL-resistance (3). Therefore, exploration of the novel strategy to optimize TRAIL-based therapeutics is still very harsh but emergent. However, the mechanism of TRAIL-resistance is incompletely understood.

A growing body of evidence has demonstrated that microRNAs (miRNAs) exert critical roles in maintenance of tissue homeostasis (4). Furthermore, a serial of miRNAs has been identified to regulate radio- or chemo-therapy resistance in a variety of tumors, including glioblastoma (5, 6). MiR-137 is one of brain-enriched microRNAs which plays an important role in differentiation of neural stem cell and significantly decreases with advanced progression of glioblastoma (7). In previous studies, it was reported that miR-137 can reverse the multiple malignant behaviors of glioblastoma cells by targeting multiple oncogenic pathways. For example, COX-2 and LRP6, which can functionally regulate cancer cell proliferation, local invasion and drug sensitivity, have been demonstrated to be direct targets of miR-137 in brain tumor cells (8, 9). In addition, it has been well documented that aberrantly high activation of EGFR signal pathway drives progression of glioblastoma, and EGFR is identified as a direct target of miR-137 in GBM. This finding further confirms that miR-137 is a tumor suppressor and plays important role in glioblastoma (10). Given application of the TRAIL-based therapy in pre-clinical trials and obstacle of TRAIL resistance for the therapeutic benefit in GBM, we wondered to know that if there is a functional link between miR-137 and TRAIL sensitivity and to further investigate a proper synergistic effect of miR-137 and TRAIL in the treatment of GBM.

In the present study, we found that exogenous expression of miR-137 increases sensitivity of TRAIL-induced apoptosis in GBM cells. By utilization of a global screening strategy, we identified that XIAP (X-linked IAP), an inhibitor of apoptosis protein (IAP) in apoptosis pathway, is a *bona fide* target directly regulated by miR-137 in a post-transcriptional gene silencing behavior. Importantly, the rescue experiment further confirmed that XIAP is essential for miR-137 to regulate sensitivity of TRAIL-induced apoptosis in GBM cells. Finally, our functional study demonstrated that combined utilization of miR-137 and TRAIL potently suppresses tumor growth *in vivo*. Collectively, our findings revealed that a miR-137-XIAP axis is a novel regulatory mechanism of TRAIL-mediated cell death and sheds a light on the avenue to explore novel strategies for the treatment of GBM.

## Materials and methods

### Bioinformatics analysis

The GSE90603, GSE63319, GSE165937 human glioblastoma datasets were acquired from the GEO database (https://www.ncbi.nlm.nih.gov/geo/). These datasets included miRNA sequencing data from both glioblastoma and normal tissues. The Venn diagram was processed using the Bioinformatics & Evolutionary Genomics (https://bioinformatics.psb.ugent.be/webtools/Venn/). The Volcano Plot was optimized using the OmicStudio tools at https://www.omicstudio.cn/tool. The GSE13030 with clinical information and matching gene expression was selected for survival analysis. The GSE154043 is composed of two subsets: GSE154041 (mRNA sequencing data) and GSE154042 (miRNA sequencing data), which was used to analyze the relationship between miR-137 and XIAP.

Predicted target genes of miR-137 were determined from the union of miRNA target predictions from TargetScan 8.0 (http://www.targetscan.org), PicTar (http://pictar.mdc-berlin.de/cgi-bin/PicTar vertebrate.cgi), PITA (https://genie.weizmann.ac.il/pubs/mir07/mir07_dyn_data.html) and microT (https://dianalab.e-ce.uth.gr/html/dianauniverse/index.php?r=microT_CDS). These genes were classified according to biological process by the PANTHER classification system (http://pantherdb.org/). The TCGA LGG (Brain Lower Grade Glioma) and GBM (Glioblastoma Multiforme) cohorts with the expression level of XIAP were acquired from the Gene Expression Profiling Interactive Analysis (http://gepia.cancer-pku.cn/). In addition, we analyzed the correlation between miR-137 and XIAP in TCGA datasets by using ENCORI (https://starbase.sysu.edu.cn).

### Cell culture

Human embryonic kidney cell lines (HEK-293T) and human glioblastoma cell lines (U-87 MG, U251) were purchased from the Type Culture Collection of the Chinese Academy of Sciences (Shanghai, China). All the cell lines were maintained in Dulbecco’s modified Eagle’s medium (DMEM) (Gibco) supplemented with 10% fetal calf serum (FCS) (BIOIND, Kibbutz, Israel). All the cells were incubated in a humidified atmosphere of 5% CO_2_ in air at 37°C.

### Transfection and lentiviral transduction

Cells were seeded in 6-well plates a day before transfection. The transfection was performed when they were at 50–70% confluence. All transfections were performed using Lipofectamine 2000 reagent (Invitrogen) following the manufacturer’s instructions. Analyses on recipient cells or further research were performed 48 hours after transfection.

For Lentivirus packaging, HEK-293T cells were seeded in 60-mm dishes before transfection, then it was performed using a transient co-transfection system with 1 μg pMD2G, 3 μg psPAX2 and 4 μg pLenti6.3-Luciferase/miR-137. For lentiviral transduction, U-87 MG was plated on 24-well plates and allowed to attach overnight. The medium was replaced with 500 μL fresh lentiviral supernatant (LV-Luciferase or LV-miR-137) and 8 mg/mL polybrene (Sigma-Aldrich, St. Louis, MO, USA). The polybrene was used to assist the uptake of viral particles. After infection, cells were screened using 5 μg/mL blasticidin (Sigma-Aldrich, St. Louis, MO, USA) for 7 days and were used for the following experiments. The primer and oligo sequences are listed in **Table 1**.

**Table 1:** Oligos used for PCR and transfection.

primers for PCR Cloning	Sequences (5’-3’)
**miR-137**	Forward: GAATTCAAACACCCGAGGAAATGAAA
	Reverse: CTCGAGAGGAAGCAGCCGAGCACA
**XIAP ORF**	Forward: GGATCCATGACTTTTAACAGTTTTGAAGG
	Reverse: CTCGAGTTAAGACATAAAAATTTTTTGCTTG
**XIAP 3’UTR (WT)**	Forward: GAATTCTTAGTCATGCAAAGATTC
	Reverse: CTGCAGGGTATTAGGATGGGAGTT
qRT-PCR primers	Sequences (5’-3’)
**GAPDH**	Forward: TCACCAGGGCTGCTTTTAAC
	Reverse: GACAAGCTTCCCGTTCTCAG
**XIAP**	Forward: AATAGTGCCACGCAGTCTACA
	Reverse: CAGATGGCCTGTCTAAGGCAA
**U6**	Forward: GTGCTCGCTTCGGCAGCACATATAC
	Reverse: AAAAATATGGAACGCTTCACGAATTTG
**miR-137**	Forward: TTATTGCTTAAGAATACGCGTAG
	Reverse: Universal Primer (QIAGEN)
oligo mimics	Sequences (5’-3’)
**Hsa-miR-137 mimics**	UUAUUGCUUAAGAAUACGCGUAG
	ACGCGUAUUCUUAAGCAAUAAUU
**Hsa-miR-137 inhibitor**	CUACGCGUAUUCUUAAGCAAUAA

### Xenograft tumor model

Six-week-old male Balb/c nude mice were purchased from the Vital River Laboratories (Beijing, China). All animal procedures were approved by the Laboratory Animal Welfare and Ethics Committee of Fourth Military Medical University (FMMU). Approximately 1×10^7^ U-87 MG cells which stably expressing Luciferase or miR-137 and suspended in 200 μL PBS, were injected subcutaneously into the left flank of each mouse, twelve mice per group. Tumor volume was monitored using a vernier caliper once every three days. Once the tumors reached 200 mm^3^ as calculated by (π × length × width^2^)/6, each group were divided into two groups. Liposomes were packed with pLenti6.3-Luciferase or pLenti6.3-TRAIL according to co-incubation. Then they were injected into the tumor multi-directionally once every four days, meanwhile the tumor volume was measured. Mice were killed by spinal dislocation when they were treated five times. Then tumors were removed and snap-frozen in liquid nitrogen for further research or fixed in formalin for immunohistochemical analysis.

### Real-time quantitative PCR analysis

Total RNA was extracted from cultured cells using TRIzol reagent (Invitrogen) according to manufacturer’s instructions. 1 μg of RNA was employed to synthesize cDNA using the PrimeScript RT Reagent Kit Perfect Real Time (TaKaRa, Dalian, China) or the miScript II RT Kit (Qiagen, Hilden, Germany). For the detection of target gene mRNA levels, we employed the Fluorescent qRT-PCR using GAPDH as an internal control. The expression levels of miR-137 in glioblastoma cells and tissues were examined by qRT-PCR assays and normalized to the expression of U6 snRNA (sn-RNU6). All the primers were obtained from AuGCT (Beijing, China) and the reactions were run in triplicates. Relative expression levels of miRNA or mRNA were acquired and analyzed using the Bio-Rad C1000 Thermal Cycler (Bio-Rad, California, CA, USA). The primer sequences are listed in **Table 1** and **Supplementary Table S1**.

### Luciferase report assay

HEK-293T cells were seeded in 48-well plates 24 h before transfection. Then cells were transfected with a mixture of 100 ng pGL3-XIAP-3’UTR, 20 μM miR-137 mimics or negative control (miR-NC), and 5 ng PRL-TK using Lipofectamine 2000 reagent and performed in three independent experiments. Firefly and Renilla luciferase activities were examined 48 h after transfection using a dual-luciferase reporter system (Promega, Madison, WI, USA).

### Western blot analysis and immunohistochemistry (IHC)

Cells were trypsinized 48 h after transfection, and Western blot analysis was performed as previously described (11). The antibodies were used as follows: XIAP (CST, cat#14334S, Danvers, MA, USA), Caspase 3 (Proteintech, cat#19677-1-AP, Rosemont, IL, USA) and GAPDH (Proteintech, cat#10494-1-AP, Rosemont, IL, USA). The caspase 3 antibody can be used to analyze both the completed and cleaved forms of caspase-3. The intensity analysis of western blotting was quantified by Image J software. The protein expression data represents relative XIAP intensity from three separate experiments, and normalized to that of GAPDH. Standard IHC staining was performed as previously described (11). In brief, following deparaffinization and rehydration, the slides were incubated with anti-XIAP antibody (CST, cat#14334S, Danvers, MA, USA, 1:100). Images were captured with a microscope (BX51 Olympus, Tokyo, Japan) and processed with identical settings.

### Cell death and cell proliferation assay

Cells were plated at 2.0×10^3^ cells per well in 96-well plates 24 h after transfection and allowed to adhere overnight prior to drug treatments. Cells were then treated with TRAIL (300 ng/mL) (Peprotech, Cranbury, NJ, USA), cell counting kit-8 assay (CCK-8) was performed (at 0, 24, 48, 72 h) to analyze cell density. For detection of caspase-3/7 activity, cells were treated with TRAIL for 24h and analyzed by using Caspase-Glo Assay kits (Promega, Madison, WI, USA) according to the manufacturer’s instructions. When using Z-VAD (10 μM) (Selleck, Houston, TX, USA), Necrosulfonamide/NSA (5 μM) (Selleck, Houston, TX, USA) and Necrostatin 1S/Nec-1S (20 μM) (MCE, Monmouth Junction, NJ, USA) to differentiate apoptotic and necroptotic cell death, cells were plated at 4.0×10^3^ cells per well in 96-well plates and cell density was measured 24 h after the treatment with those agents.

Cells were transfected with different mimics or genes 24 h before TRAIL treatment, then they were collected and suspended in PBS after 48 h TRAIL treatment. The obtained cells were stained with FITC Annexin-V Apoptosis Detection Kit I (BD Biosciences, Franklin Lakes, NJ, USA) and were analyzed by flow cytometry (FCM). Meanwhile, Cell apoptosis in xenograft tumor tissues was detected by TUNEL staining using an *In Situ* Cell Death Detection Kit, Fluorescein (Roche, Mannheim, Germany) according to the manufacturer’s protocol. The nuclei were then counterstained with DAPI (Sigma-Aldrich, St. Louis, MO, USA). Subsequently, samples were subjected to laser scanning confocal microscopy analysis (Nikon, Tokyo, Japan).

### Patient samples

The glioblastoma samples (n = 10) and paired adjacent normal tissues (n = 10) were obtained from the department of Neurosurgery at Xijing Hospital in the Fourth Military Medical University (FMMU). Collection of clinical samples was approved by the Medical Ethics Committee of Xijing Hospital. All specimens were promptly snap-frozen using liquid nitrogen and stored at -80°C. The tissues were used for RNA isolation when needed and were used to analyze the expression level of miR-137 by using qRT-PCR assay.

### Statistical analyses

Statistical analyses were performed using GraphPad Prism version 8.00 (GraphPad Inc., USA). All the data were exhibited as the mean ± SD of at least three independent experiments. The differences between groups were analyzed using two-sided Student’s t-test or two-way ANOVA, and a p value < 0.05 was considered statistically significant.

## Results

### MiR-137 expression was decreased in glioblastoma tissues

**Figure 1:**
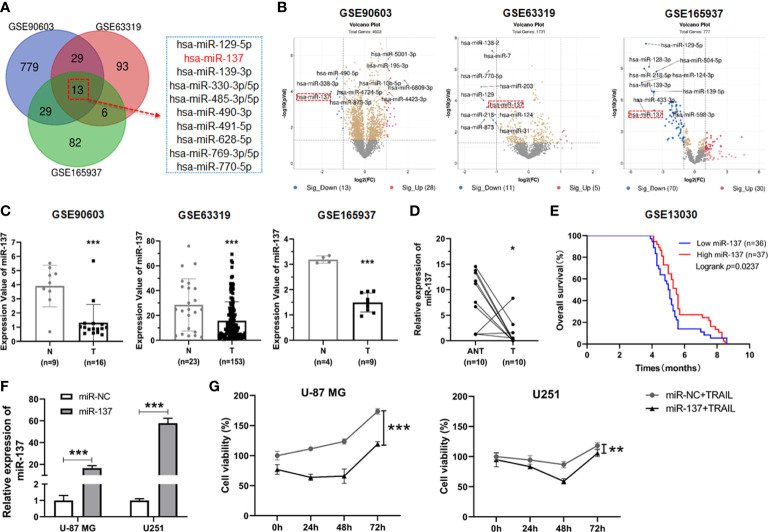
miR-137 is reduced in tumor tissues of glioblastoma patients and correlated with the survival rate in clinic. **(A)** Bioinformatic interrogation of Gene Expression Omnibus (GEO) datasets. The downregulated miRNAs in glioblastoma samples compared with normal brain tissues. Identify key miRNAs related to glioblastoma by combining three GEO datasets. **(B)** The differential expression miRNAs of GEO datasets (threshold set as |log_2_(Fold Change)|≥1, *P* < 0.05). Here’s the top ten miRNAs in each GEO datasets. **(C)** The expression value of miR-137 in glioblastoma (T) and normal (N) tissues (GSE90603, GSE63319, GSE165937). (***P < 0.001.) **(D)** Relative expression of miR-137 in carcinoma (T) and adjacent normal tissues (ANT) of glioblastoma patients (n = 10) measured by qRT-PCR. [The p value was determined by Paired-samples t-test. (*P < 0.05)]. **(E)** Kaplan–Meier overall survival (OS) curves according to miR-137 expression levels in glioblastoma cohorts. **(F)** qRT-PCR analysis of the relative miR-137 expression levels in U-87 MG/U251 cells transfected with the miR-137 or miR-NC mimics. (***P < 0.001.) **(G)** Cell viability of U-87 MG/U251 cells initially transfected with miR-NC or miR-137 24 h followed by TRAIL treatment determined by CCK-8 assay. [Data were presented as the mean ± SD of different groups. The p value was analyzed by Student’s t-test or two-way ANOVA, n = 3. **P < 0.01, ***P < 0.001 relative to controls].

Given miRNA dysregulation frequently occurs in glioblastoma, we wondered to identify the functional miRNA(s) associated with progression of GBM. By combined utilization of bioinformatic analysis of raw data from a serial of GEO datasets, we identified twelve downregulated miRNAs in GBM tissues compared with normal brain tissues (**Figure 1A**). Among all of them, miR-129-5P, miR-139, miR-330, *etc.* have been reported to function as tumor suppressors in GBM previously (12–14). These evidences further confirmed the reliability of our screening strategy. In these selected miRNAs, miR-137 is one of top ten differential expressed miRNAs in all tested GEO datasets (**Figure 1B**). Accordingly, we focused on miR-137 for further analysis. We first analyzed the expression levels of miR-137 in the three GEO datasets, the results showed that the miR-137 expression in GBM tissues is significantly lower than that in normal tissues (**Figure 1C**). To further confirm this expression pattern, we collected fresh tissues and performed qRT-PCR to examine the expression level of miR-137 in GBM tissues and adjacent normal tissues from the GBM patients. The data showed a significant downregulation of miR-137 in cancer tissues compared with the paired normal tissues (**Figure 1D**). Subsequently, in order to test whether miR-137 can be a potential clinical prognostic indicator, we analyzed the relationship between the miR-137 expression and the survival rate in clinic. The result showed that the higher level of miR-137 is closely associated with the favorable prognosis in clinic (**Figures 1E**). These results indicated that miR-137 might be a potential tumor suppressor in glioblastoma.

### MiR-137 sensitizes GBM cells to the TRAIL-mediated apoptosis

It has been reported previously that GBM cells differentially respond to TRAIL-induced cell death (11, 15). Given that miR-137 plays a pivotal role in neuronal differentiation and downregulates in glioma genesis, we wondered to determine the biological role of miR-137 on the sensitivity of TRAIL-induced cell death in GBM cells. We transiently transfected TRAIL-low responsive GBM cell lines (U-87MG and U251 cells) with miR-137 mimics and negative control (miR-NC). MiR-137 was overexpressed in the two GBM cell lines respectively (**Figure 1F**). Then the transfected cells were incubated with the TRAIL recombinant proteins and cell density were measured. The synergistic inhibitory effects for miR-137 in combination with TRAIL were observed in the two cell lines, which suggested that TRAIL can further induce GBM cell death in the presence of miR-137 (**Figure 1G**).

**Figure 2:**
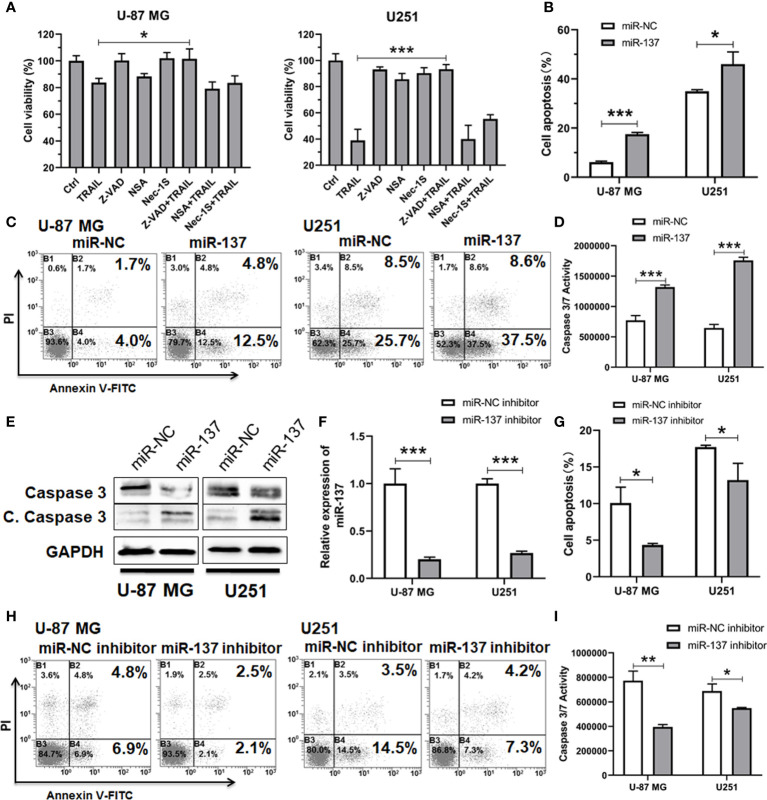
miR-137 increased the sensitivity of U-87 MG/U251 cells to TRAIL-induced apoptosis. **(A)** Cell viability of U-87 MG/U251 cells initially transfected with miR-137 followed by 24 h treatment with TRAIL and/or different inhibitor determined by CCK-8 assay. Z-VAD, pan-caspase inhibitor; NSA (Necrosulfonamide), MLKL inhibitor; Nec-1s (Necrostatin 2 racemate), RIPK1 inhibitor. **(B)** Cell apoptosis of U-87 MG/U251 cells initially transfected with miR-NC or miR-137 24 h followed by 48 h TRAIL treatment determined by FCM analysis with Annexin-V and PI staining. **(C)** Representative pictures of cell apoptosis. **(D)** Caspase-3/7 assay after treatment of U-87 MG/U251 cells with miR-NC/137 and TRAIL. Cell apoptosis reflected by caspase-3/7 activity.**(E)** Western blotting analyses of Caspase-3 and Cleaved Caspase-3 expression. Increased cell apoptosis reflected by the decrease of caspase-3 and the increase of cleaved caspase-3 (C. Caspase 3). **(F)** qRT-PCR analysis of the relative miR-137 expression levels in U-87 MG/U251 cells transfected with the miR-NC or miR-137 inhibitor. **(G)** Cell apoptosis of U-87 MG/U251 cells treated with miR-NC/137 inhibitor and TRAIL. **(H)** Representative pictures of cell apoptosis. **(I)** Caspase-3/7 assay after treatment with miR-NC/137 inhibitor and TRAIL. [Data were presented as the mean ± SD of different groups. The p value was analyzed by Student’s t-test, n = 3. *P < 0.05, **P < 0.01, ***P < 0.001 relative to controls].

To clarify the types of cell death that TRAIL induced in combination with miR-137, we assessed the effects of pan-caspase (Z-VAD), RIPK1 (Necrostatin 1S) and MLKL (Necrosulfonamide) inhibitors on cell density in the two GBM cell lines. All the two cell lines demonstrated rescue from inhibitory effects of miR-137 and TRAIL with pan-caspase inhibitors Z-VAD (**Figure 2A**). These suggested that GBM cell sensitivity to miR-137 with TRAIL is predominantly mediated by caspase-dependent apoptotic cell death. Then cells were assessed by the Annexin-V staining. Flow cytometry analysis revealed that overexpression of miR-137 raises the proportion of apoptotic cells evidently with regard to their respective controls (**Figures 2B**, **C**). The pro-apoptotic effects were also confirmed through caspase-3/7 activity and protein levels of caspase-3 (**Figures 2D**, **E**). Furthermore, the miR-137 inhibitor was introduced into U-87 MG and U251 cells, respectively (**Figures 2F**). As expected, knockdown of the endogenous miR-137 decreases the effects which were induced by TRAIL in GBM cells (**Figures 2G**–**I**). These findings suggested that overexpression of miR-137 sensitizes GBM cells to TRAIL-induced apoptosis.

### XIAP is suppressed in miR-137-overexpressed GBM cells

**Figure 3:**
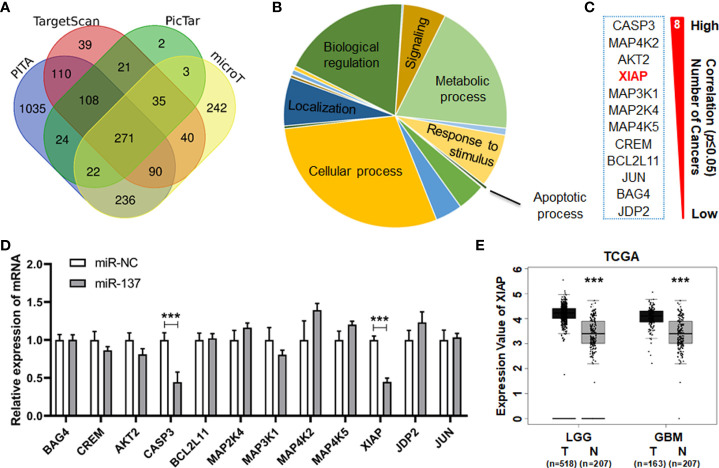
miR-137 may be the regulator of XIAP. **(A)** Predicted target genes of miR-137 collected from four prediction algorithms (PITA, TargetScan, PicTar and microT). **(B)** All the predicted target genes classified by biological process according to the PANTHER classification system (http://pantherdb.org/ ). Identify critical genes related to apoptotic process. **(C)** Gene list correlated with the apoptosis signaling pathway. The reverse relationship between miR-137 and these candidate target genes were analyzed in TCGA database (ENCORI website). The number of cancers showed how many tumor types the negative correlation existed. (*P < 0.05) **(D)** qRT-PCR analysis depicting the changes in the expression pattern of these candidate target genes in miR-NC/miR-137-treated U251 cells. [Data were exhibited as mean ± SD, n = 3. ***P < 0.001 using Student’s t-test.] **(E)** The expression value of XIAP in tumor (T) and normal (N) tissues of LGG (Brain Lower Grade Glioma) and GBM (Glioblastoma Multiforme) cohorts. (***P < 0.001).

To identify the downstream target(s) of miR-137 which can regulate sensitivity of TRAIL-induced cell death in the GBM cells, we firstly used four prediction websites (PITA, TargetScan, PicTar and microT) to analyze the putative target genes of miR-137 (**Figure 3A**). The predicted target genes from the algorithms were further analyzed according to the PANTHER classification system. Twelve genes were clustered in the apoptosis signaling pathway (**Figure 3B**). The reverse relationship between miR-137 and these genes were analyzed in TCGA database. Among them, we focused on XIAP (X-chromosome Inhibitory apoptotic protein), which functionally antagonizes apoptotic stimulation in transformed cells. More importantly, we found that miR-137 expression level is inversely correlated with XIAP expression in five types of cancers (**Figure 3C**). Using qRT-PCR, we confirmed that the mRNA level of XIAP is downregulated in miR-137-overexpressed U251 cells (**Figure 3D**). Additionally, we found that the expression level of XIAP significantly decreases in LGG and GBM samples compared with the control in the TCGA database (**Figure 3E**). These data suggested that XIAP is suppressed by mir-137 in GBM cells, and it may be a target of miR-137.

### XIAP is a *bona fide* target of miR-137 in GBM cells

**Figure 4:**
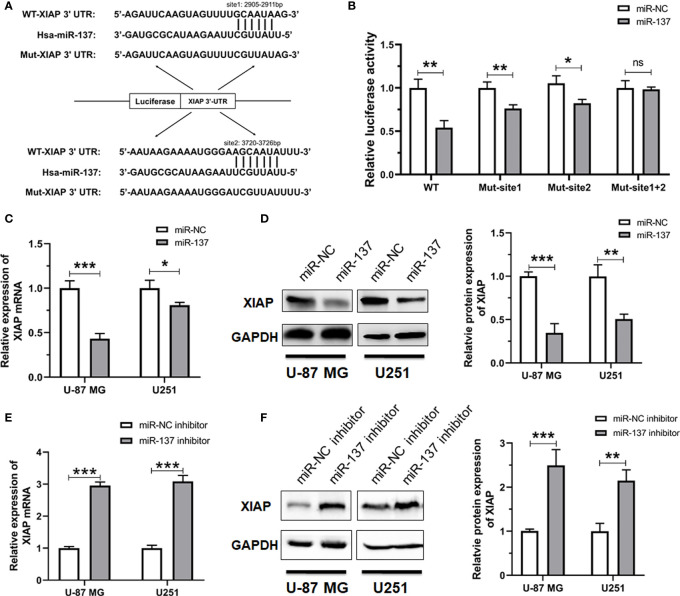
XIAP is a *bona fide* target of miR-137 in GBM cells. **(A)** The potential binding sites of miR-137 to the 3’UTR of human XIAP. The wild-type and mutant XIAP 3’UTR were constructed into pGL3 vectors. **(B)** Relative luciferase activity analyses in HEK-293T cells co-transfected with Luciferase-XIAP 3’UTR fusion and miR-NC/137. [The luciferase activity was examined 48 h after transfection. Data represents relative firefly luciferase units from three separate experiments, and normalized to that of renilla luciferase]. (ns: not significant, *P < 0.05, **P < 0.01) **(C)** qRT-PCR analyses of XIAP mRNA, **(D)** Western blotting analyses of XIAP expression (left panel), the intensity analysis of western blotting (right panel) in U-87 MG/U251 cells transfected with the miR-NC/137. **(E)** qRT-PCR analyses of XIAP mRNA, **(F)** Western blotting analyses of XIAP expression in cells treated with the miR-NC or miR-137 inhibitor. [The analyses were performed 48 h after transfection. Data were exhibited as mean ± SD, n = 3. *P < 0.05, **P < 0.01, ***P < 0.001 relative to controls].

According to the silicon analysis, two predicted binding sites of miR-137 present on the 3’UTR region of XIAP (**Figure 4A**). To determine whether miR-137 recognizes these two putative sites (2905–2911 bp and 3720–3726 bp), we constructed luciferase reporter plasmids containing wild-type or target site 1- or site 2-mutant 3’UTR of XIAP. The indicated plasmids were co-transfected into HEK-293T cells with miR-NC or miR-137 mimics. The transfection efficiency was normalized by co-transfection with renilla reporter vector. Overexpression of miR-137 significantly decreased the normalized luciferase activity of wild-type XIAP 3’UTR, but had slight effect on any mutation of XIAP 3’UTR (**Figure 4B**). Subsequently, we attempted to validate whether miR-137 could downregulate the expression level of XIAP in GBM cells. Using qRT-PCR analysis, we found that the mRNA level of XIAP significantly decreases when the miR-137 was overexpressed in the GBM cells (**Figure 4C**). Consistent with the reduction of the XIAP mRNA level, Western blot analysis also showed that enforced expression of miR-137 dramatically reduced the XIAP protein level (**Figure 4D**). Meanwhile, the miR-137 inhibitor was introduced into U-87 MG and U251 cells, respectively. As expected, knockdown of the endogenous miR-137 increased XIAP mRNA and protein levels in GBM cells (**Figures 4E**, **F**).

**Figure 5:**
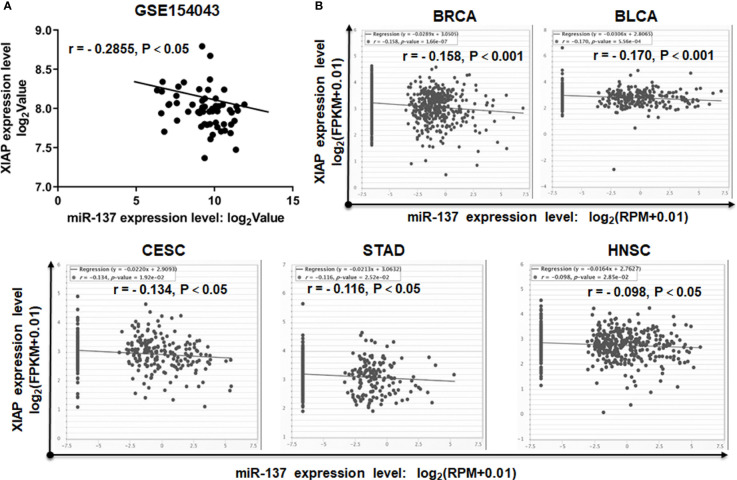
miR-137 negatively correlates with XIAP mRNA levels in cancer patient samples. **(A)** The correlation between miR-137 and XIAP expression in glioblastoma cohorts (GSE154043). [The p value was determined by Pearson correlation test]. **(B)** TCGA Correlation analysis of miR-137 and XIAP in TCGA database (BRCA, BLCA, CESC, STAD and HNSC). The relative levels of miR-137 were plotted against that of XIAP using the online tool (http://starbase.sysu.edu.cn/).

To further determine the correlation between the expression levels of miR-137 and XIAP, we acquired the miRNA and mRNA sequencing data of GBM samples from the GEO database. The expression level of miR-137 is reversely correlated with the expression of XIAP (**Figure 5A**). More strikingly, by mining the data from TCGA, we also found the negative correlation between miR-137 and XIAP in multiple types of cancers, including breast cancer (BRCA), bladder urothelial cancer (BLCA), cervical squamous cell carcinoma and endocervical adenocarcinoma (CESC), stomach adenocarcinoma (STAD) and head and neck squamous cell carcinoma (HNSC) (**Figure 5B**). All the data showed that there is a general negative correlation between miR-137 and XIAP in cancer samples. Thus, these results indicated that XIAP is a *bona fide* target of miR-137 in GBM cells, or even in other types of cancers.

### XIAP is essential for miR-137-regulated sensitivity of TRAIL-induced cell death in GBM cells *in vitro*

**Figure 6:**
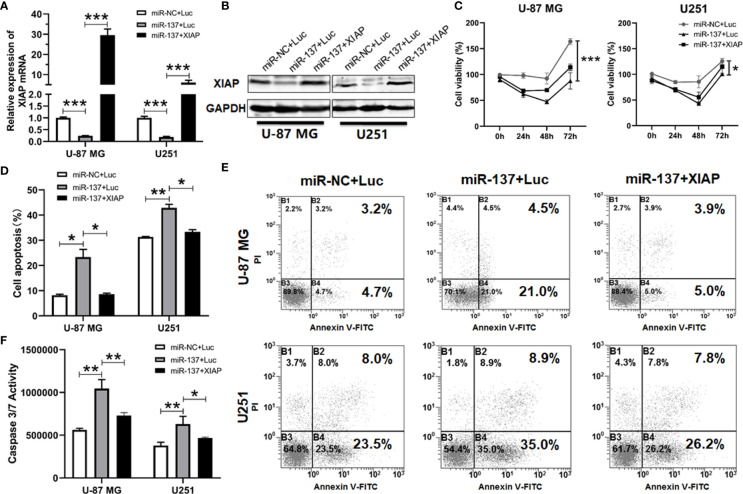
XIAP treatment abrogated miR-137-increased sensitivity to TRAIL-induced apoptosis in glioblastoma cells. **(A)** qRT-PCR and **(B)** Western blotting analyses of XIAP expression in miR-NC/137 with/without XIAP-treated sets in U-87 MG and U251 cells. The analyses were performed 48 h after treatment. **(C)** Cell viability of U-87 MG/U251 cells treated with different molecules and TRAIL determined by CCK-8 assay. **(D)** Flow cytometry analyses for cell apoptosis. Cells were initially treated with above sets 24 h followed by 48 h TRAIL treatment, then were determined by FCM analysis with Annexin-V and PI staining. **(E)** Representative pictures of cell apoptosis. **(F)** Caspase-3/7 assay after treatment with miR-NC/137, Luc/XIAP and TRAIL. Cell apoptosis reflected by caspase-3/7 activity. [Data were presented as mean ± SD of different groups, n = 3. *P < 0.05, **P < 0.01, ***P < 0.001 using Student’s t-test].

Given that XIAP is a negative regulator of apoptosis in cancer cells and is a direct target of miR-137 in GBM cells, we hypothesized that a miR-137-XIAP axis could play a functional role in regulating sensitivity of GBM cells to TRAIL-induced cell death. To test the hypothesis, we simultaneously transfected both XIAP-expressing plasmid and miR-137 mimics into GBM cells. Western blot and qRT-PCR analyses validated the rescued expression of exogenous XIAP in GBM cells transfected with both miR-137 and XIAP-expressing plasmid (**Figures 6A**, **B**). Furthermore, cell density and Annexin-V staining assay showed that the exogenously expressed XIAP abrogated the miR-137-triggerred TRAIL sensitivity in GBM cells (**Figures 6C**–**E**). Meanwhile, caspase-3/7 assay also confirmed the effects (**Figure 6F**). The results demonstrated that XIAP is potential negative regulator for mir-137-mediated TRAIL sensitivity in GBM cells *in vitro*.

### Combined utilization of miR-137 and TRAIL potently suppresses tumor growth *in vivo*

**Figure 7:**
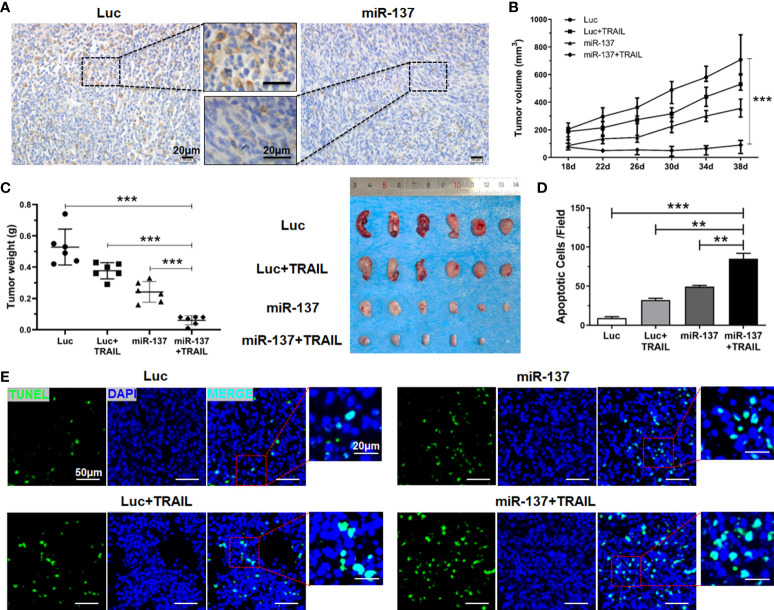
miR-137 cooperating with TRAIL suppressed tumor growth in nude mice. **(A)** IHC analysis depicting the changes of XIAP expression level in excised tumors using anti-XIAP antibody, with scale bars = 20 µm. **(B)** Graphical representation of the changes in the tumor volume followed by subcutaneous injection of pLenti6.3-Luciferase/TRAIL plasmid for a period of 3 weeks (n = 6 per group). **(C)** Tumor weight of the excised tumors from the different treatment sets (left panel), representative appearance of tumor mass resected from each group of mice (right panel). **(D)** TUNEL staining assay showing the apoptotic cells of the excised tumors in each group. [Apoptotic cells were averaged in six random fields each group]. (**P < 0.01, ***P < 0.001.) **(E)** Representative fields of cell apoptosis, with scale bars = 20 µm or 50 µm.

**Figure 8:**
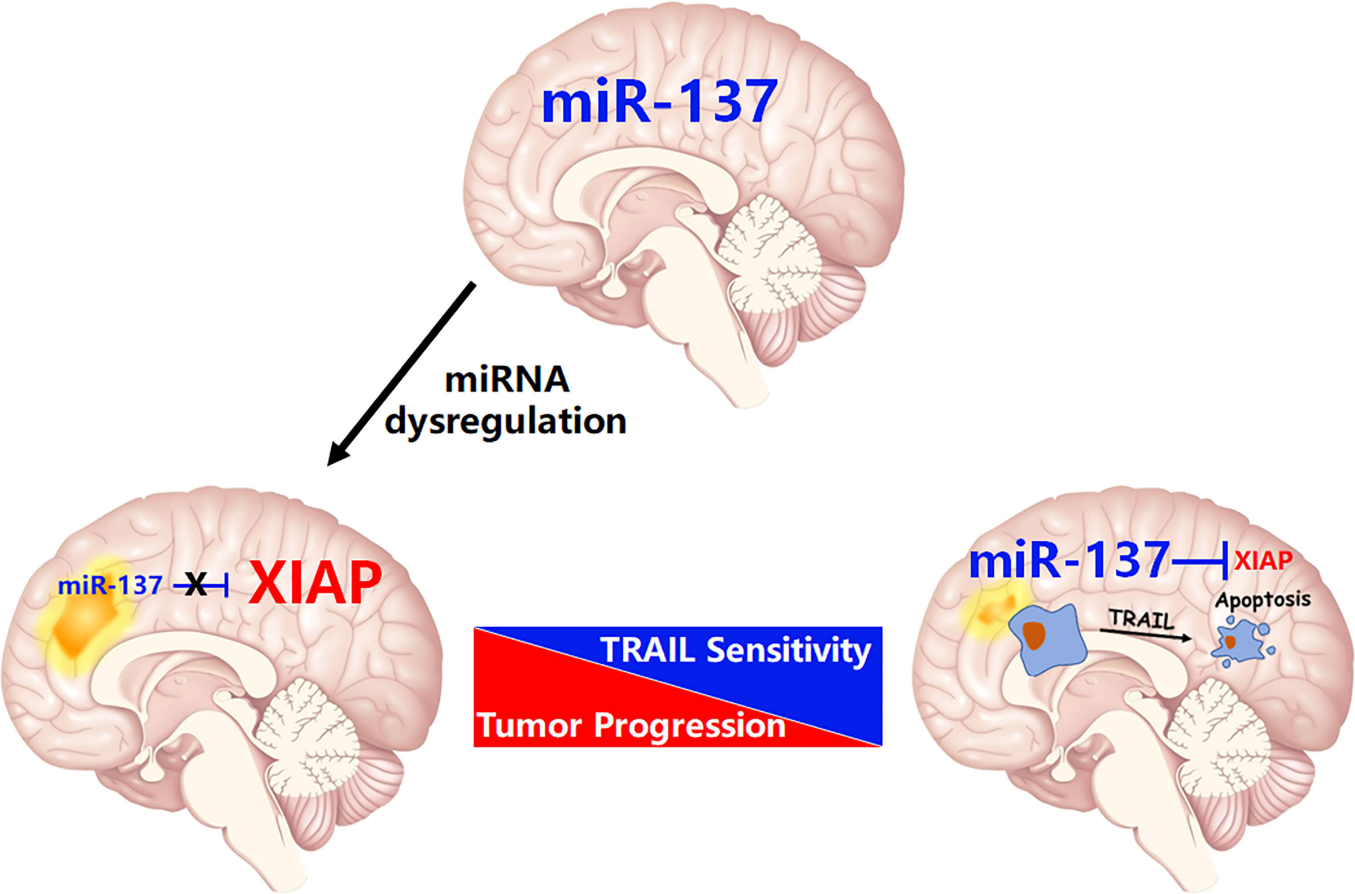
The schematic diagram of this study: miR-137 suppresses XIAP expression to sensitize TRAIL-induced cell death in GBM.

To determine the therapeutic benefit of combined utilization of miR-137 and TRAIL *in vivo*, we first investigated the inhibitory effect of miR-137 on the expression of XIAP in U-87 MG cell-derived xenografts. The results of IHC staining showed that overexpression of miR-137 significantly inhibited endogenous expression of XIAP *in vivo* (**Figure 7A**). Furthermore, in the established xenograft mouse model, we used liposome-capsuled TRAIL-expressing plasmid to perform a multipoint injection on tumor tissues in miR-137-overexpressed or control xenografts. Each mouse was administrated liposome-capsuled TRAIL-expressed or control plasmids for five times every four days. Although monotherapy with miR-137 overexpression or TRAIL administration led to anti-tumor effect *in vivo*, combined utilization of miR-137 and TRAIL had a more significant effect on regression of tumor growth (**Figures 7B**, **C**). Subsequently, the combined effect was also confirmed by the number of apoptotic cancer cells using TUNEL staining assay (**Figures 7D**, **E**). Taken together, the above results further demonstrated that overexpression of miR-137 could be a potential strategy to improve the efficacy of TRAIL-based therapeutics in GBM (**Figure 8**).

## Discussions

For decades, most research in the field of cancer biology focused on the involvement of protein-coding genes. Only in recent two decades, it was found that an entire class of molecules, termed microRNA (miRNA), plays key roles in regulating cellular activity (16). According to its expression pattern and function in development and progression of malignant diseases, it is termed as oncomiR (oncogenic miRNA) or tumor suppressor miRNA (17). Consequently, an increasing number of cancer-associated clinical trials have been carried out involving miRNAs as novel biomarkers or therapeutic reagents (18–20). The powerful role of miRNA helps us to extend our knowledge on the cancer biology (21).

Each miRNA is often able to regulate more than one target, and vice versa, mRNAs are frequently targeted by several miRNAs. As such, miRNAs function as the master regulators that control the expression of thousands of coding genes or non-coding RNAs, including most of the oncogenes, such as MYC, RAS, EGFR, and the potent tumor suppressors, including TP53, PTEN, and BRCA1 (21, 22). In our recent study, by utilization of a systemic functional screening for miRNAs regulating TRAIL-induced apoptosis in GBM cells, we identified that miR-7, one of brain-enriched miRNAs, sensitizes TRAIL-induced cell death in GBM cells. The mechanistic study showed that XIAP, one of IAP family members, is essential for miR-7-invovlved regulation of TRAIL sensitivity (11). More strikingly, data from Shah’s group further confirms the potential role of miR-7 on sensitization of TRAIL-induced apoptosis in GBM cells. They found that miR-7 upregulates death receptor 5 and primes apoptosis in resistant glioblastoma (23). In the present study, we identified that miR-137, another brain-enriched miRNA, is also an upstream regulator of XIAP (**Figure 8**). This finding extends our knowledge of miRNA-mediated negative regulation of XIAP in transformed cells.

Although TRAIL has demonstrated tremendous promise in a wide range of human cancer cell lines in recent years, a proportion of cancer cells including GBM harbor innate resistance. Here, we established a functional link between miR-137 and TRAIL sensitivity in GBM. However, it was recently reported that the necroptosis may also be involved in TRAIL-induced cell death. For example, it was reported that combinational treatment with TRAIL and smac mimetics triggers necroptosis in Burkitt’s lymphoma cells (24), virus-induced cochlear hair cell death is mediated by TRAIL-induced necroptosis (25). Therefore, we confirmed the type of cell death induced by TRAIL and miR-137 in GBM cells. As shown in **Figure 2A**, TRAIL combined with miR-137 induces caspase-dependent cell apoptosis. It occurs coincidently with a recent study that miR-137 promotes apoptosis in ovarian cancer cells *via* downregulation of XIAP (26). What is more, as showed in the **Figure 5B**, miR-137 is negatively correlated with the expression of XIAP in multiple types of cancers, including BRCA, BLCA, STAD, CESC and HNSC. All the evidence emphasizes that a miR-137-XIAP axis is a universal regulatory mechanism in cancers. With the development of miRNA biology, a growing body of evidence demonstrates that tissue or cell-type specific expression is one of characteristics of miRNAs. For instance, more than 70% abundance of miR-122 is expressed in hepatocytes (27, 28), miR-1 is mostly specifically expressed in heart tissues (29), miR-206 is abundantly expressed in skeletal muscle (30, 31). The tissue-specific miRNAs play a critical role on maintenance of tissue homeostasis. It is also identified that a bunch of miRNAs are highly expressed in the central nervous system. More importantly, a serial of recent studies reported that a module of miRNAs, constituted by miR-124, miR-128 and miR-137, are co-expressed during neuronal differentiation and simultaneously lost in glioma genesis (32, 33). These results indicate that brain-enriched miRNAs potentially regulate terminal differentiation and functions as tumor suppressors. Among them, miR-137 is one of the earliest reported functional miRNAs in neurons. It has been reported that miR-137 induces differentiation of brain tumor stem cells and inhibits proliferation of GBM cells by post-transcriptional repression of cyclin-dependent kinase 6 (CDK6) (34).

In recent years, as reported that miR-137 inhibits proliferation, angiogenesis, metastasis and ferroptosis in multiple cancers, including GBM (35). These studies have identified a serial of downstream targets regulating progression of GBM, such as cyclin-dependent kinase 6 (CDK6), epidermal growth factor receptor (EGFR) and enhancer of zeste homolog 2 (EZH2) etc. (7). It has also been reported recently that stanniocalcin-1 (STC1), a secreted glycoprotein, may act as a novel metastasis/metastatic dissemination-promoting factor regulated by miR-137 in GBM (36). In addition, it was reported that direct inhibition of SLC1A5 by miR-137 enhances glioma cell oxidative stress and triggers lipid peroxidation, which eventually induces tumor cell death (37). However, the role of miR-137 in regulating the sensitivity of TRAIL-induced apoptosis in tumor cells is still unknown. Here, by combined utilization of bioinformatic prediction, reporter assay, qRT-PCR and Western blot, we validated that XIAP, which exerts a critical role as an antagonist of cell apoptosis, is a novel direct target of miR-137 in GBM. Our *in vitro* and *in vivo* experiments indicate that a miR-137-XIAP axis contributes to the sensitivity of TRAIL-induced cell death in glioblastoma. Our findings facilitate a better understanding of therapeutic resistance of glioblastoma and suggest that combined utilization of miR-137 and TRAIL could be a potent strategy in the treatment of glioblastoma.

## Data availability statement

The original contributions presented in the study are included in the article/**Supplementary material**. Further inquiries can be directed to the corresponding authors.

## Ethics statement

The studies involving human participants were reviewed and approved by Medical Ethics Committee of Xijing Hospital in the Fourth Military Medical University (FMMU). The patients/participants provided their written informed consent to participate in this study. The animal study was reviewed and approved by Laboratory Animal Welfare and Ethics Committee of Fourth Military Medical University (FMMU).

## Author contributions

XZ, JZ, and SH contributed to conception and design of the study. FG and FY performed experiments, analyzed the data, and wrote the first draft of the study. JHZ provided clinical samples. FL and JHZ performed bioinformatics analysis, analyzed the data, and wrote part of the manuscript related to clinical significance. RZ analyzed the results and improved the manuscript. All authors contributed to manuscript revision, read, and approved the submitted version.

## Funding

This research was supported by the National Natural Science Foundation of China (No. 81773262, 81903149, 31770909 and 82173046), the Science and Technology Department of Shanxi Province, China (2018JC-013), the Natural Science Basic Research Project of Shaanxi Province, China (No. 2021SF-058 and 2020JM-322), the Natural Science Foundation of Hainan Province, China (No. 819QN377) and Scientific Research Fund Project of Xijing hospital (No. XJZT19ML39).

## Acknowledgments

We thank the staff of GSE90603, GSE63319, GSE165937, GSE13030, GSE154043 and TCGA database for their hard work. The authors are grateful to Jingtao Hu for his excellent technical assistance.

## Conflict of interest

The authors declare that the research was conducted in the absence of any commercial or financial relationships that could be construed as a potential conflict of interest.

## Publisher’s note

All claims expressed in this article are solely those of the authors and do not necessarily represent those of their affiliated organizations, or those of the publisher, the editors and the reviewers. Any product that may be evaluated in this article, or claim that may be made by its manufacturer, is not guaranteed or endorsed by the publisher.
