# Supplementary material for: A miR-137-XIAP axis contributes to the sensitivity of TRAIL-induced cell death in glioblastoma
Source: Front Oncol. 2022 Jul 28;12:870034. doi: 10.3389/fonc.2022.870034 (PMC9366219; doi:10.3389/fonc.2022.870034)
Supplement: Supplementary file 1 [file DataSheet_1.zip › Original source data - Western blot-Revised/WB Easy to assess.pptx]

## Slide 1
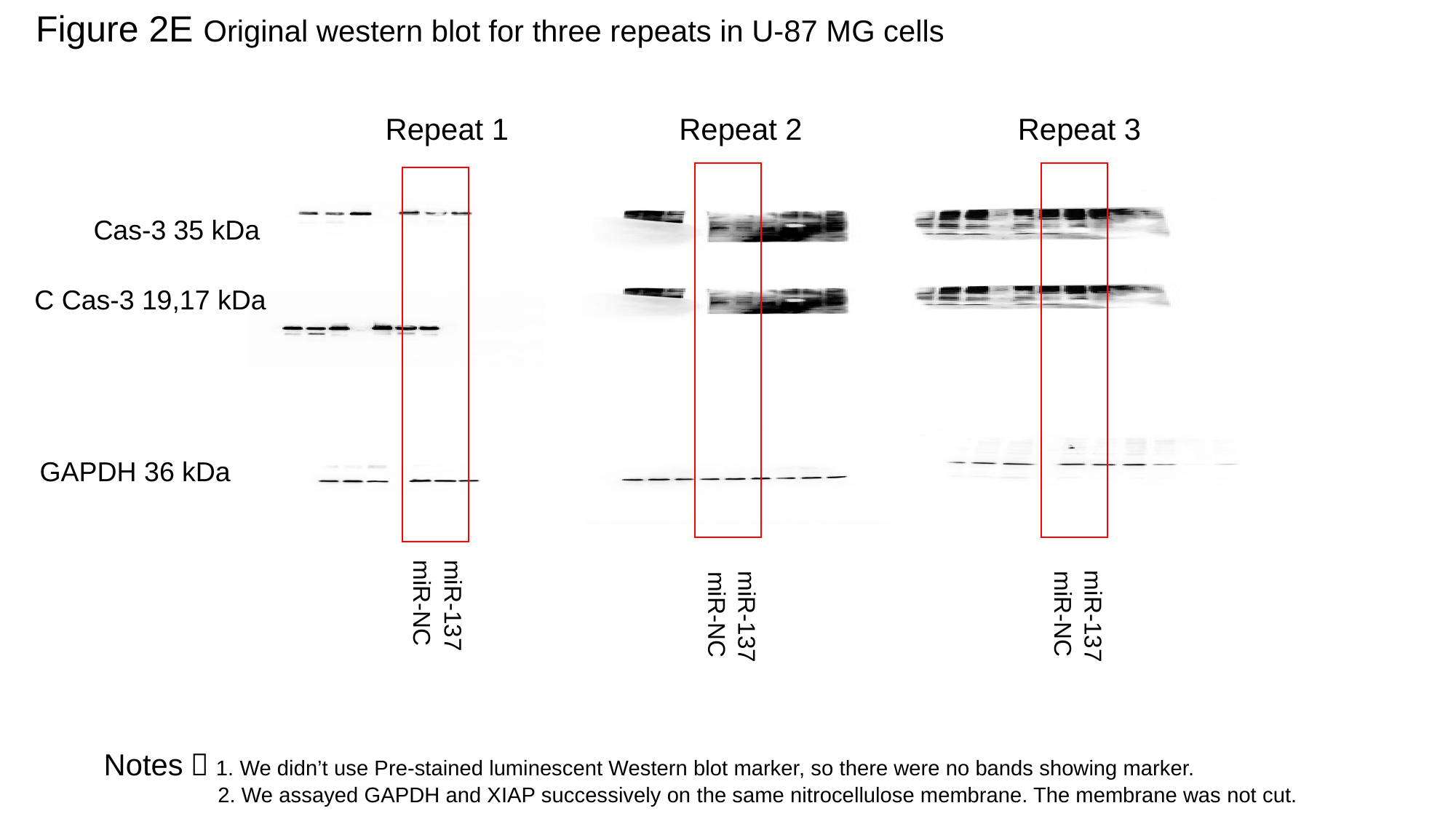

Figure 2E Original western blot for three repeats in U-87 MG cells
Repeat 1
Repeat 2
Repeat 3
Cas-3 35 kDa
C Cas-3 19,17 kDa
GAPDH 36 kDa
miR-NC
miR-137
miR-NC
miR-NC
miR-137
miR-137
Notes：1. We didn’t use Pre-stained luminescent Western blot marker, so there were no bands showing marker.
 2. We assayed GAPDH and XIAP successively on the same nitrocellulose membrane. The membrane was not cut.

## Slide 2
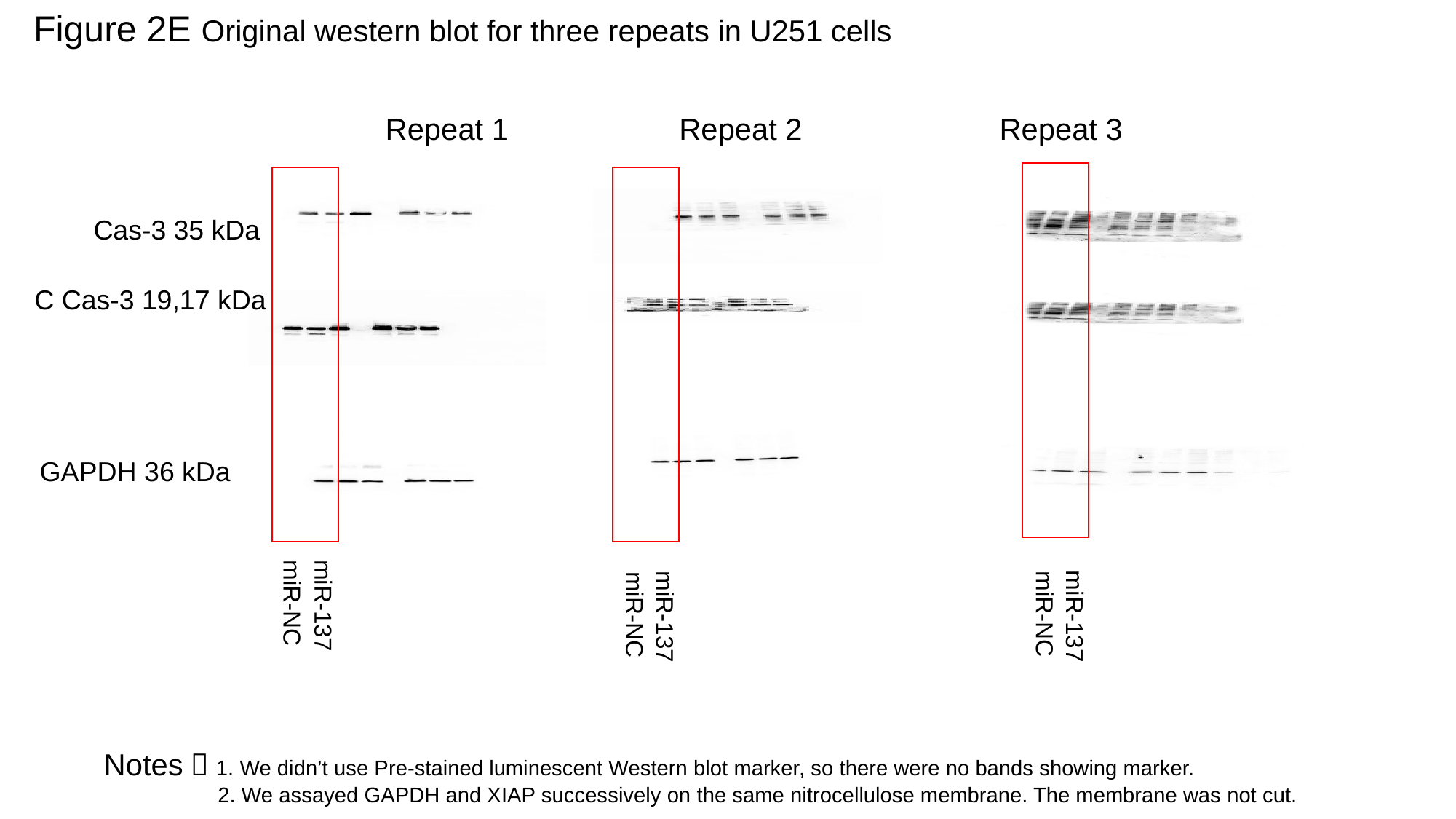

Figure 2E Original western blot for three repeats in U251 cells
Repeat 1
Repeat 2
Repeat 3
Cas-3 35 kDa
C Cas-3 19,17 kDa
GAPDH 36 kDa
miR-NC
miR-137
miR-NC
miR-NC
miR-137
miR-137
Notes：1. We didn’t use Pre-stained luminescent Western blot marker, so there were no bands showing marker.
 2. We assayed GAPDH and XIAP successively on the same nitrocellulose membrane. The membrane was not cut.

## Slide 3
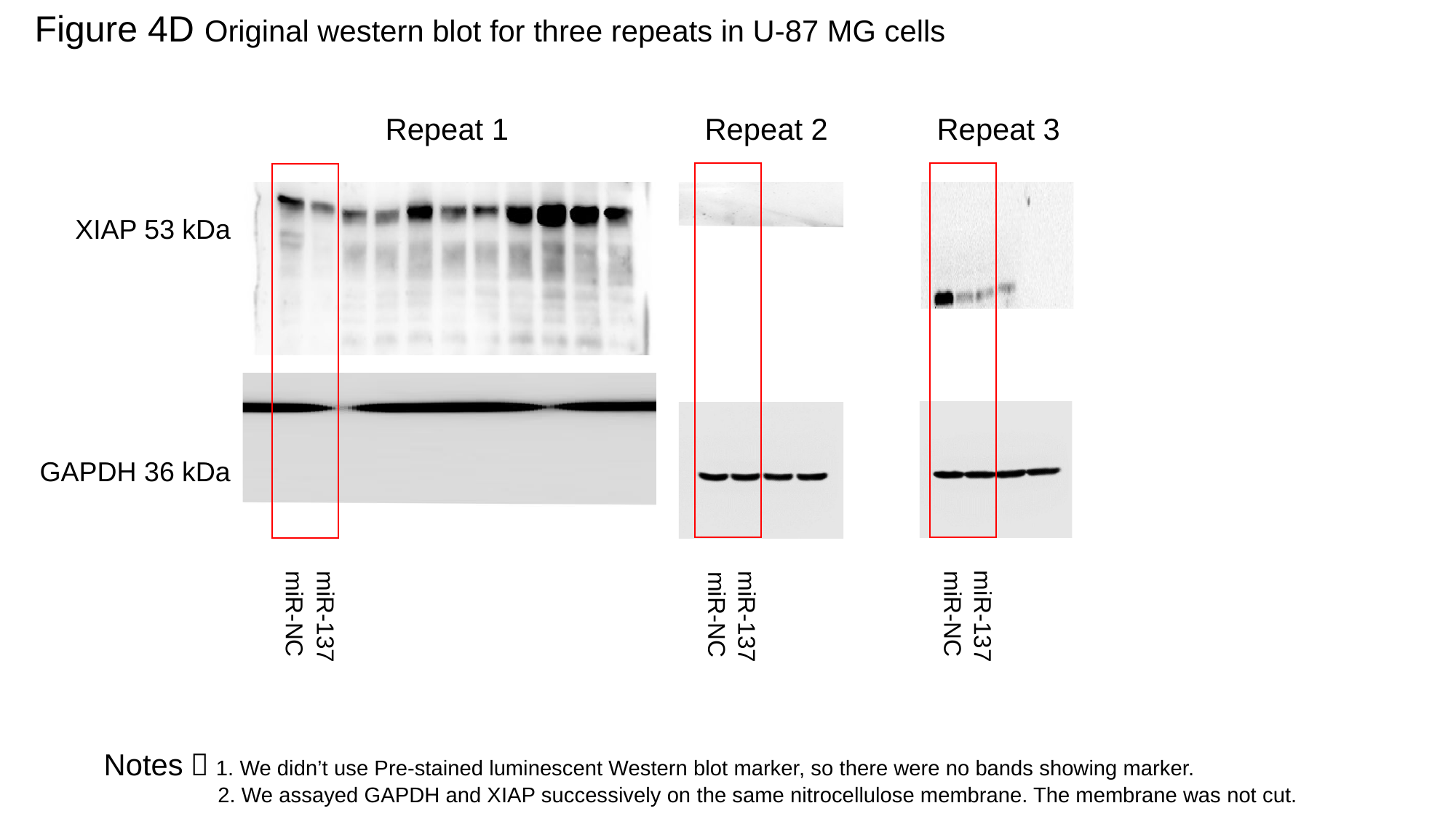

Figure 4D Original western blot for three repeats in U-87 MG cells
Repeat 1
Repeat 2
Repeat 3
XIAP 53 kDa
GAPDH 36 kDa
miR-NC
miR-NC
miR-NC
miR-137
miR-137
miR-137
Notes：1. We didn’t use Pre-stained luminescent Western blot marker, so there were no bands showing marker.
 2. We assayed GAPDH and XIAP successively on the same nitrocellulose membrane. The membrane was not cut.

## Slide 4
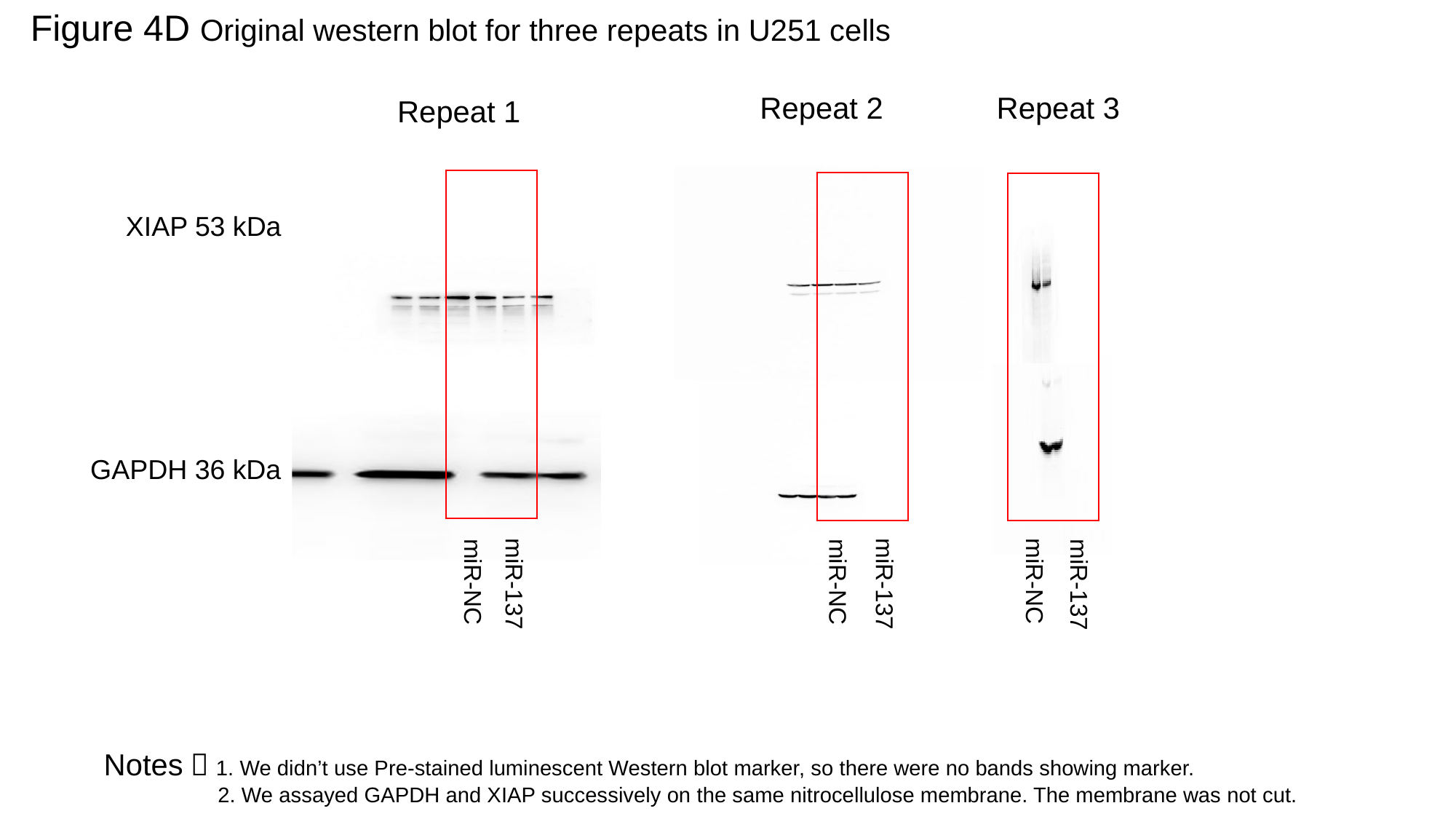

Figure 4D Original western blot for three repeats in U251 cells
Repeat 2
Repeat 3
Repeat 1
XIAP 53 kDa
GAPDH 36 kDa
miR-NC
miR-NC
miR-NC
miR-137
miR-137
miR-137
Notes：1. We didn’t use Pre-stained luminescent Western blot marker, so there were no bands showing marker.
 2. We assayed GAPDH and XIAP successively on the same nitrocellulose membrane. The membrane was not cut.

## Slide 5
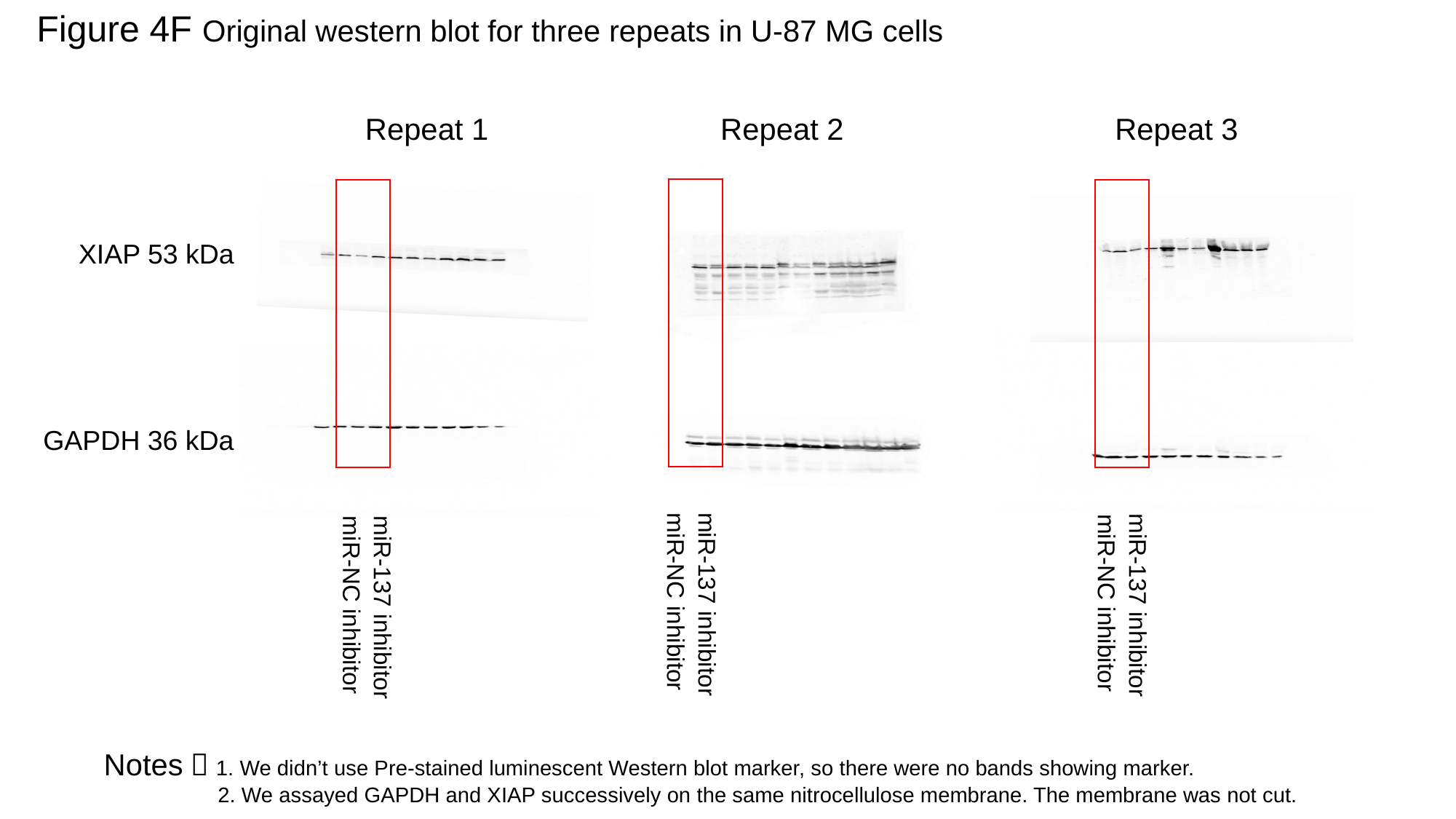

Figure 4F Original western blot for three repeats in U-87 MG cells
Repeat 1
Repeat 2
Repeat 3
XIAP 53 kDa
GAPDH 36 kDa
miR-NC inhibitor
miR-NC inhibitor
miR-137 inhibitor
miR-NC inhibitor
miR-137 inhibitor
miR-137 inhibitor
Notes：1. We didn’t use Pre-stained luminescent Western blot marker, so there were no bands showing marker.
 2. We assayed GAPDH and XIAP successively on the same nitrocellulose membrane. The membrane was not cut.

## Slide 6
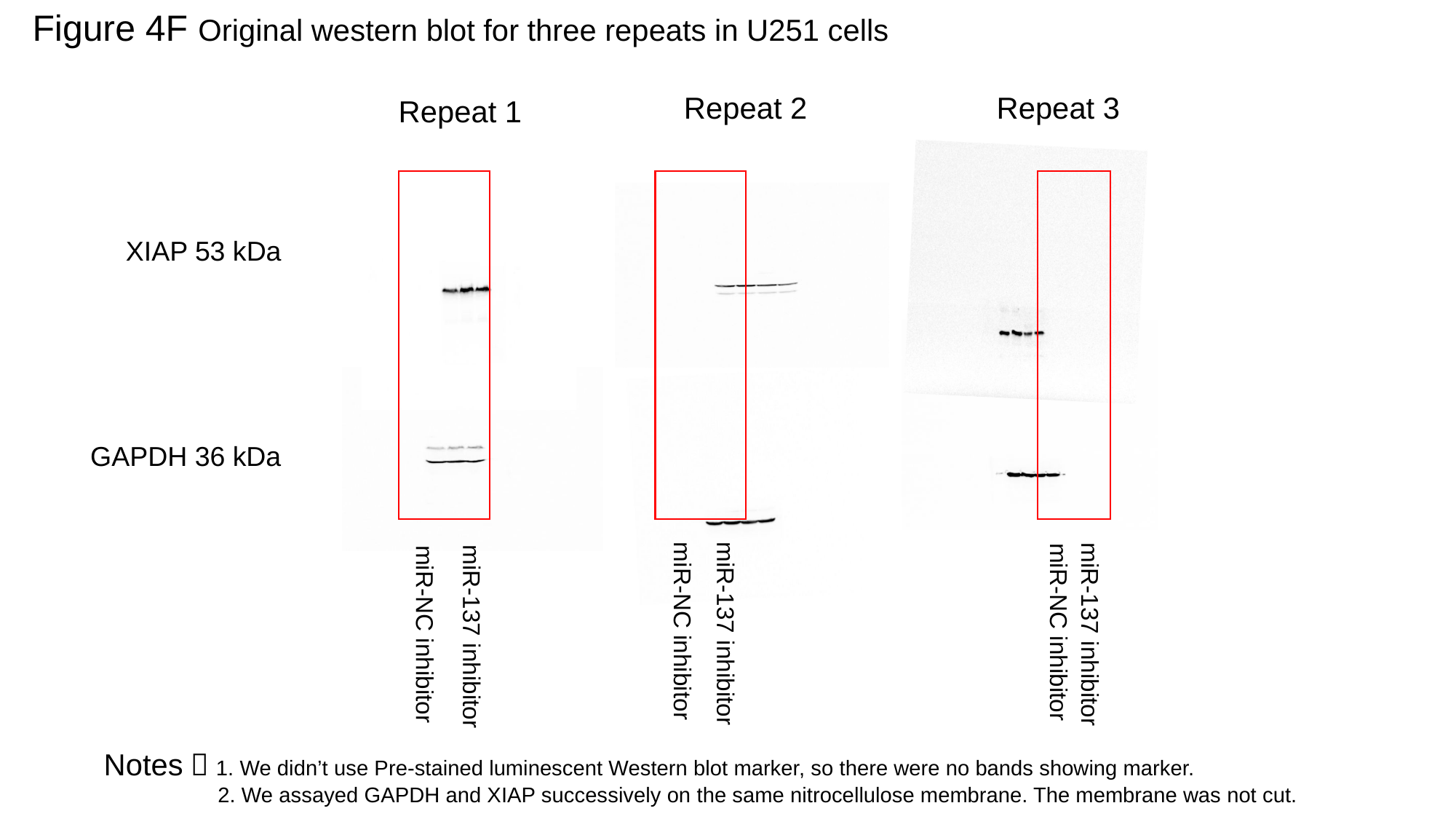

Figure 4F Original western blot for three repeats in U251 cells
Repeat 2
Repeat 3
Repeat 1
XIAP 53 kDa
GAPDH 36 kDa
miR-NC inhibitor
miR-NC inhibitor
miR-137 inhibitor
miR-NC inhibitor
miR-137 inhibitor
miR-137 inhibitor
Notes：1. We didn’t use Pre-stained luminescent Western blot marker, so there were no bands showing marker.
 2. We assayed GAPDH and XIAP successively on the same nitrocellulose membrane. The membrane was not cut.

## Slide 7
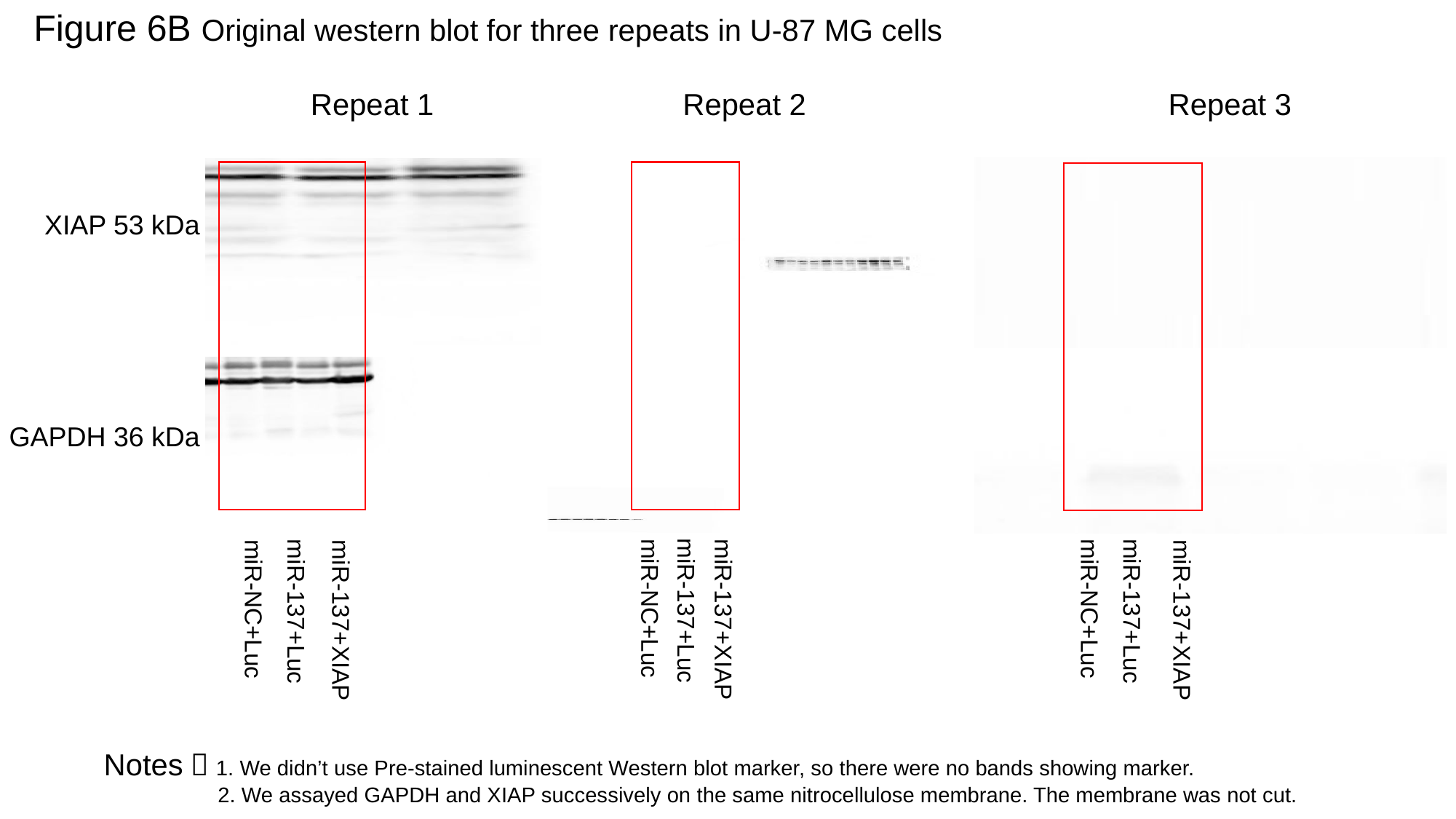

Figure 6B Original western blot for three repeats in U-87 MG cells
Repeat 1
Repeat 2
Repeat 3
XIAP 53 kDa
GAPDH 36 kDa
miR-NC+Luc
miR-NC+Luc
miR-NC+Luc
miR-137+Luc
miR-137+Luc
miR-137+Luc
miR-137+XIAP
miR-137+XIAP
miR-137+XIAP
Notes：1. We didn’t use Pre-stained luminescent Western blot marker, so there were no bands showing marker.
 2. We assayed GAPDH and XIAP successively on the same nitrocellulose membrane. The membrane was not cut.

## Slide 8
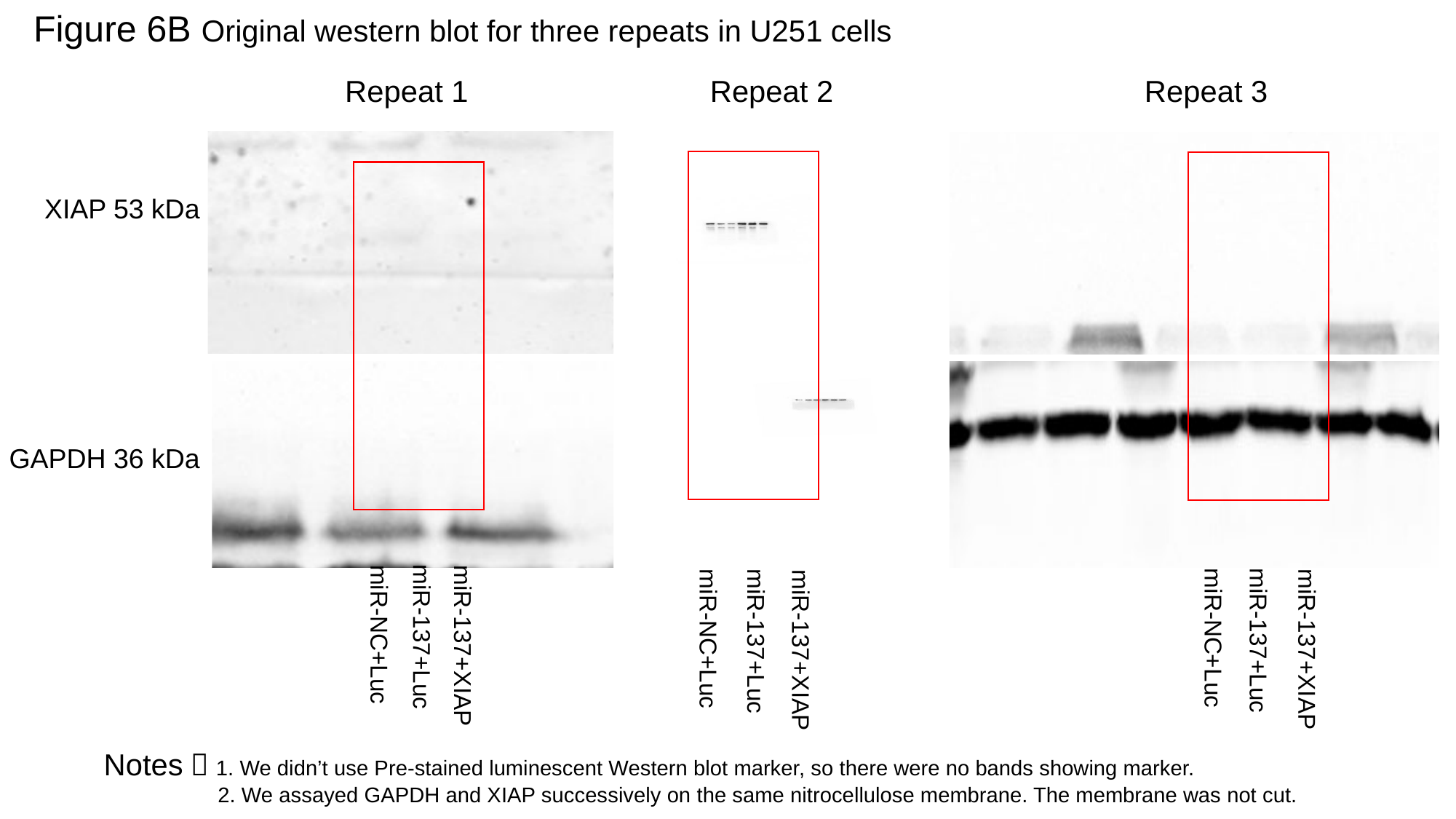

Figure 6B Original western blot for three repeats in U251 cells
Repeat 1
Repeat 2
Repeat 3
XIAP 53 kDa
GAPDH 36 kDa
miR-NC+Luc
miR-137+Luc
miR-NC+Luc
miR-NC+Luc
miR-137+Luc
miR-137+Luc
miR-137+XIAP
miR-137+XIAP
miR-137+XIAP
Notes：1. We didn’t use Pre-stained luminescent Western blot marker, so there were no bands showing marker.
 2. We assayed GAPDH and XIAP successively on the same nitrocellulose membrane. The membrane was not cut.
