# Supplementary figures and images for: A miR-137-XIAP axis contributes to the sensitivity of TRAIL-induced cell death in glioblastoma
Source: Front Oncol. 2022 Jul 28;12:870034. doi: 10.3389/fonc.2022.870034 (PMC9366219; doi:10.3389/fonc.2022.870034)

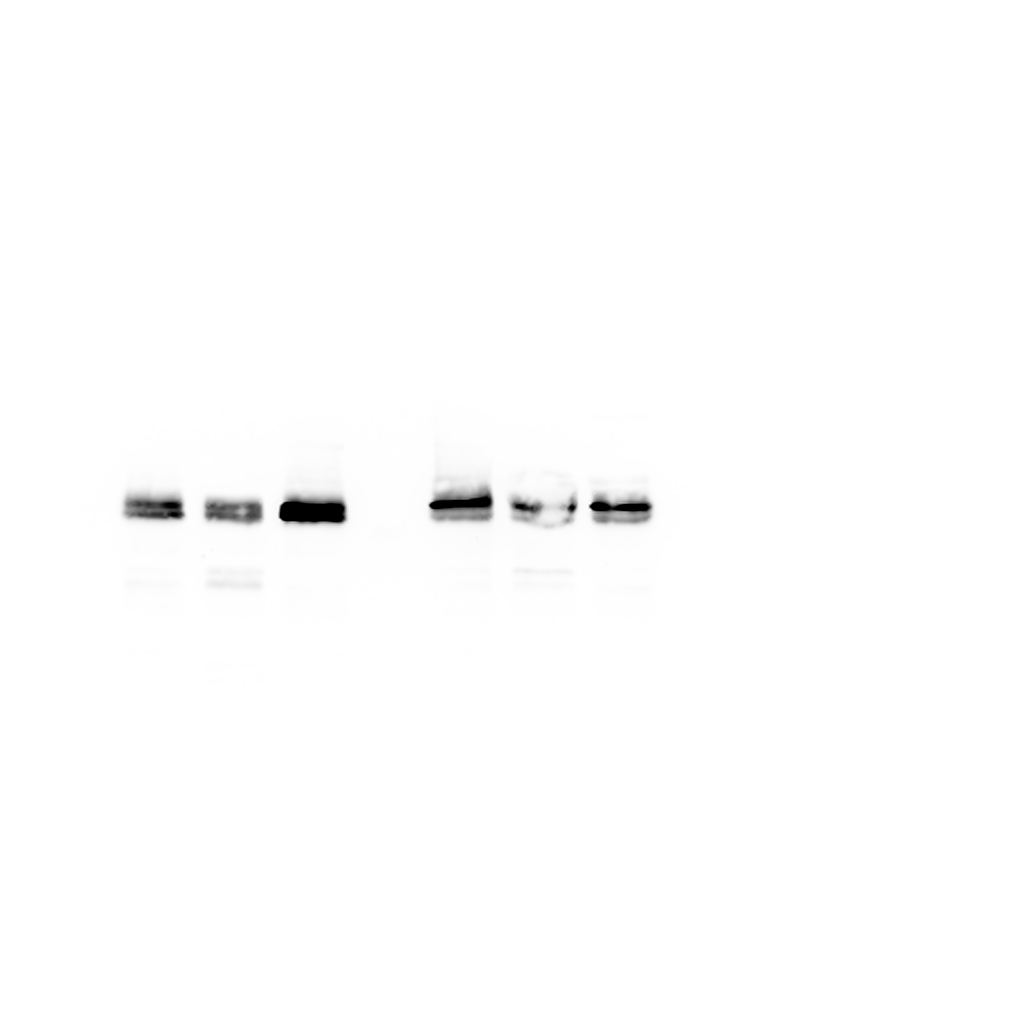

Supplement: Supplementary file 1 [file DataSheet_1.zip › Original source data - Western blot-Revised/Figure 2E/Cas-3 Repeat-1 U87 4-5 lane, U251 1-2 lane.tif]

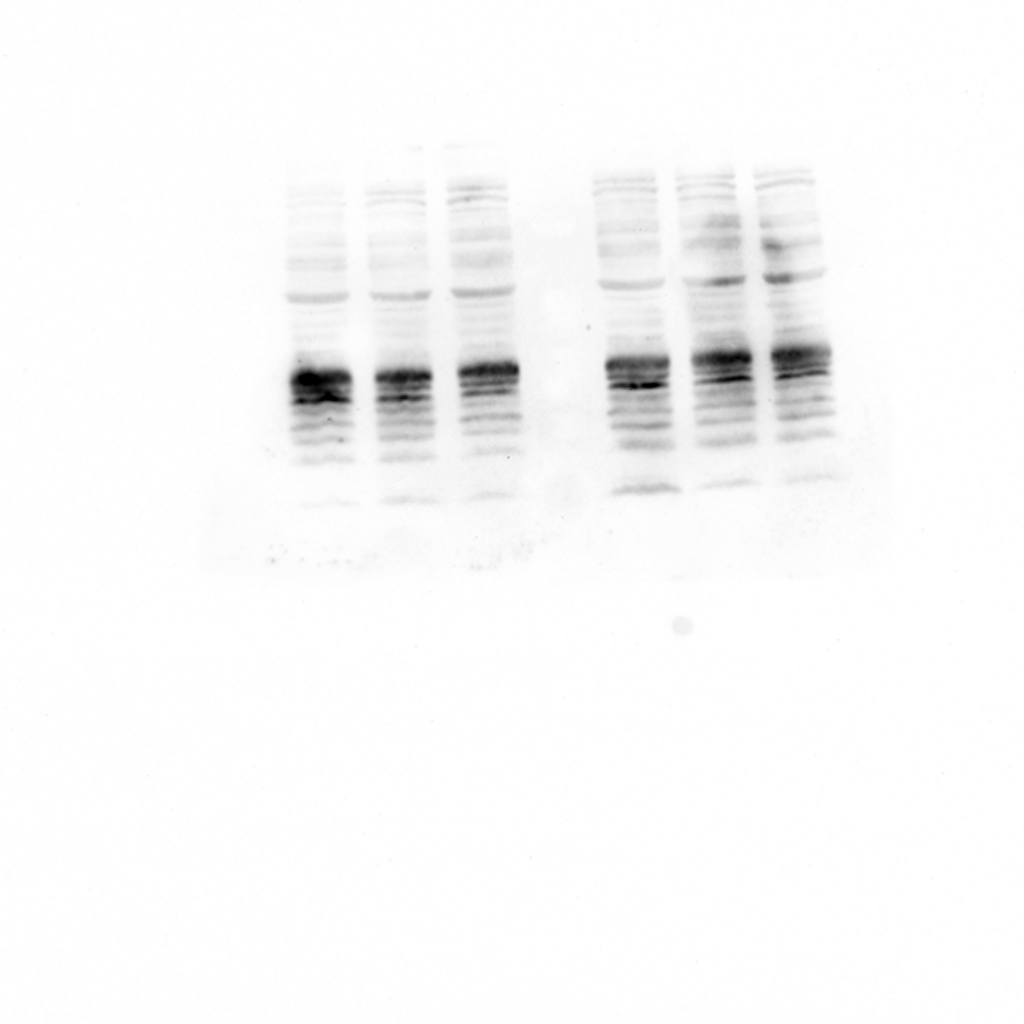

Supplement: Supplementary file 1 [file DataSheet_1.zip › Original source data - Western blot-Revised/Figure 2E/Cas-3 Repeat-2 U251 1-2 lane.tif]

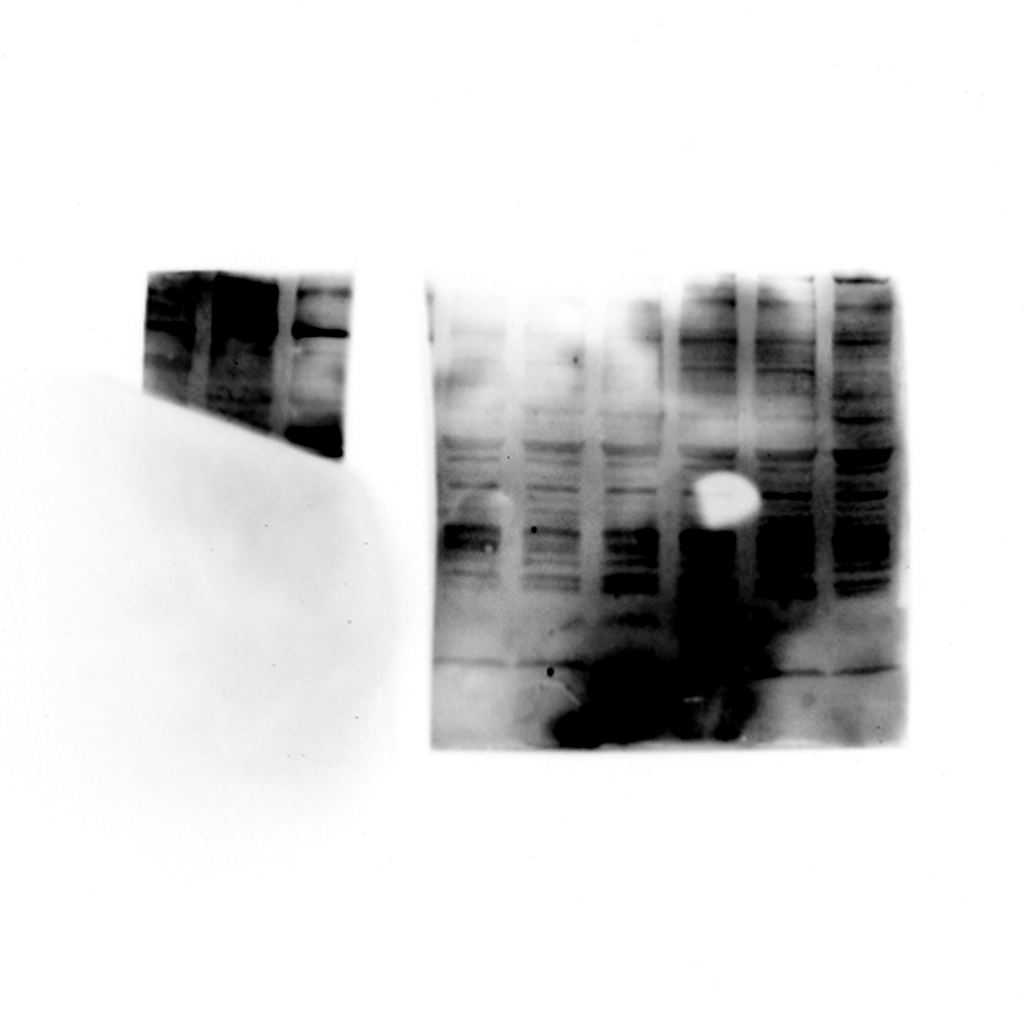

Supplement: Supplementary file 1 [file DataSheet_1.zip › Original source data - Western blot-Revised/Figure 2E/Cas-3 Repeat-2 U87 4-5 lane.tif]

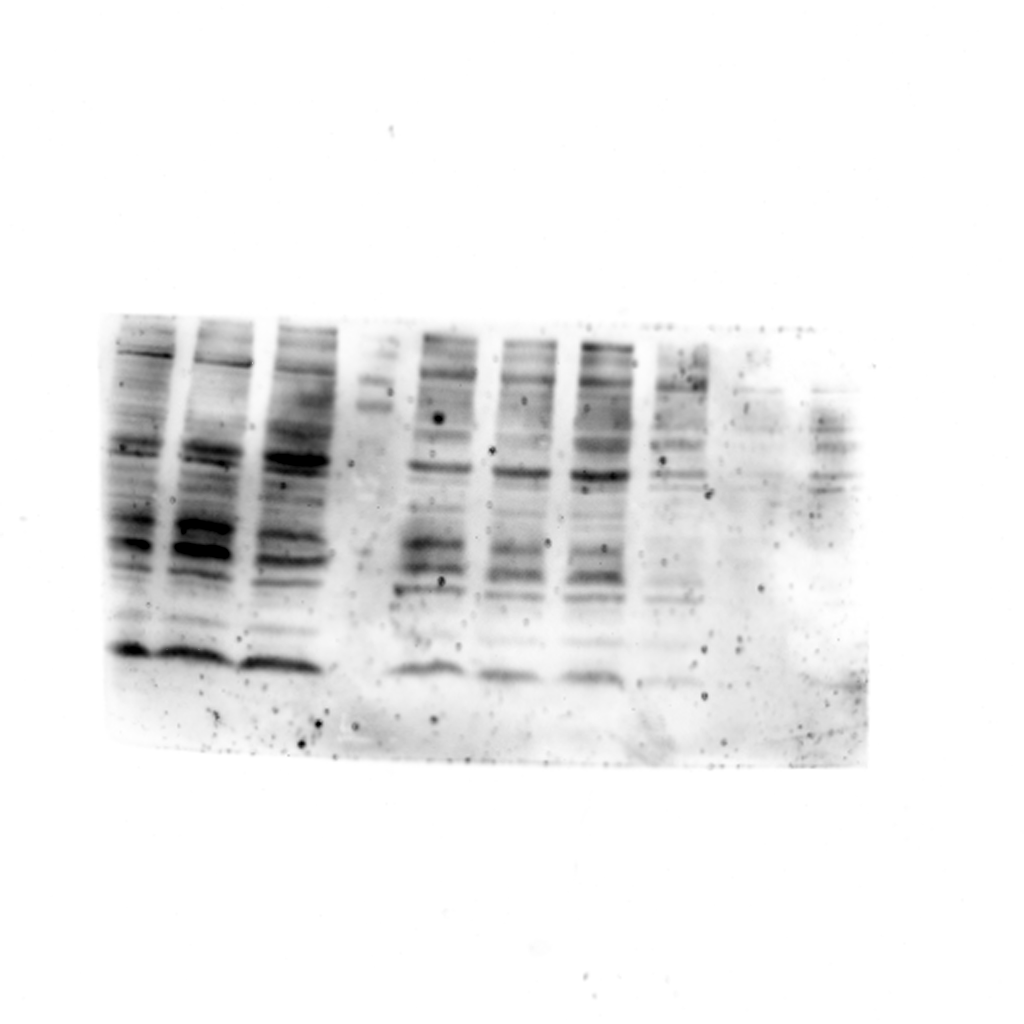

Supplement: Supplementary file 1 [file DataSheet_1.zip › Original source data - Western blot-Revised/Figure 2E/Cas-3 Repeat-3 U251 2-3 lane.tif]

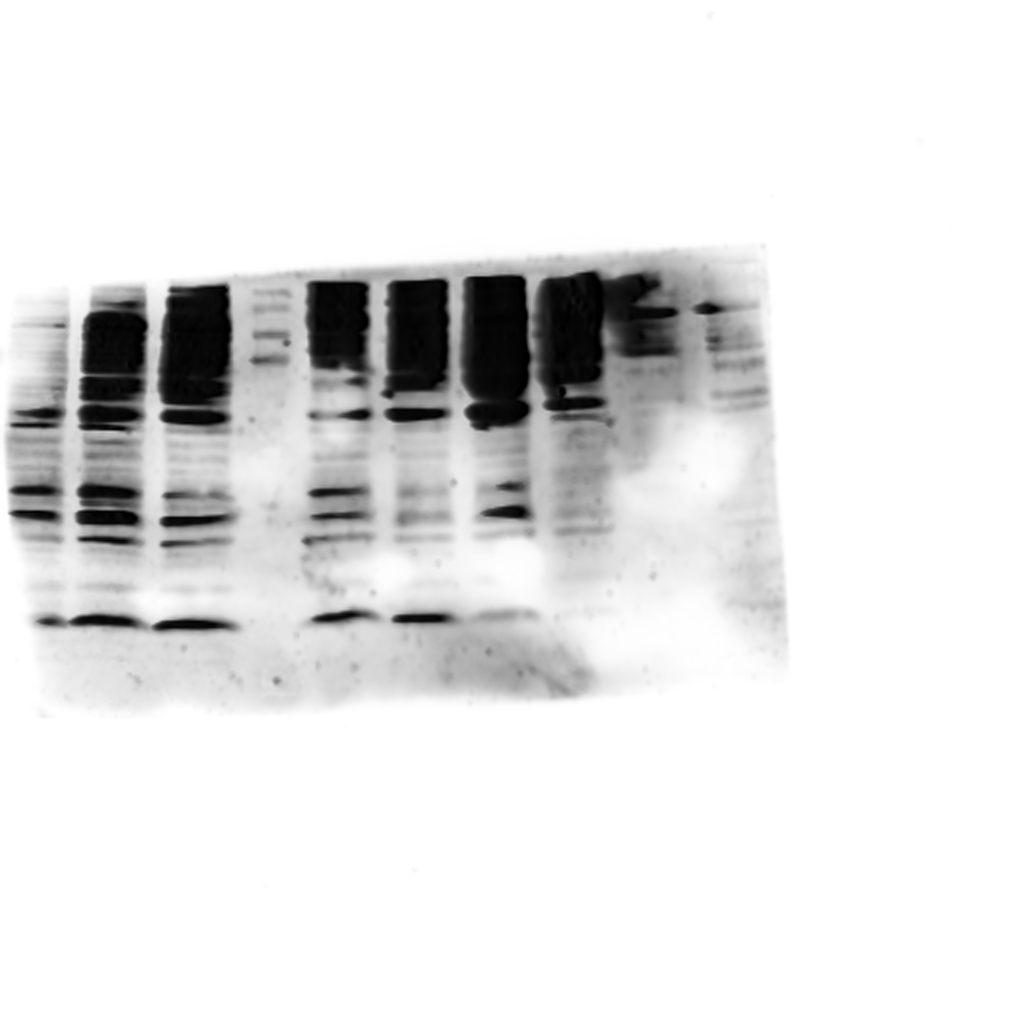

Supplement: Supplementary file 1 [file DataSheet_1.zip › Original source data - Western blot-Revised/Figure 2E/Cas-3 Repeat-3 U87 4-5 lane.tif]

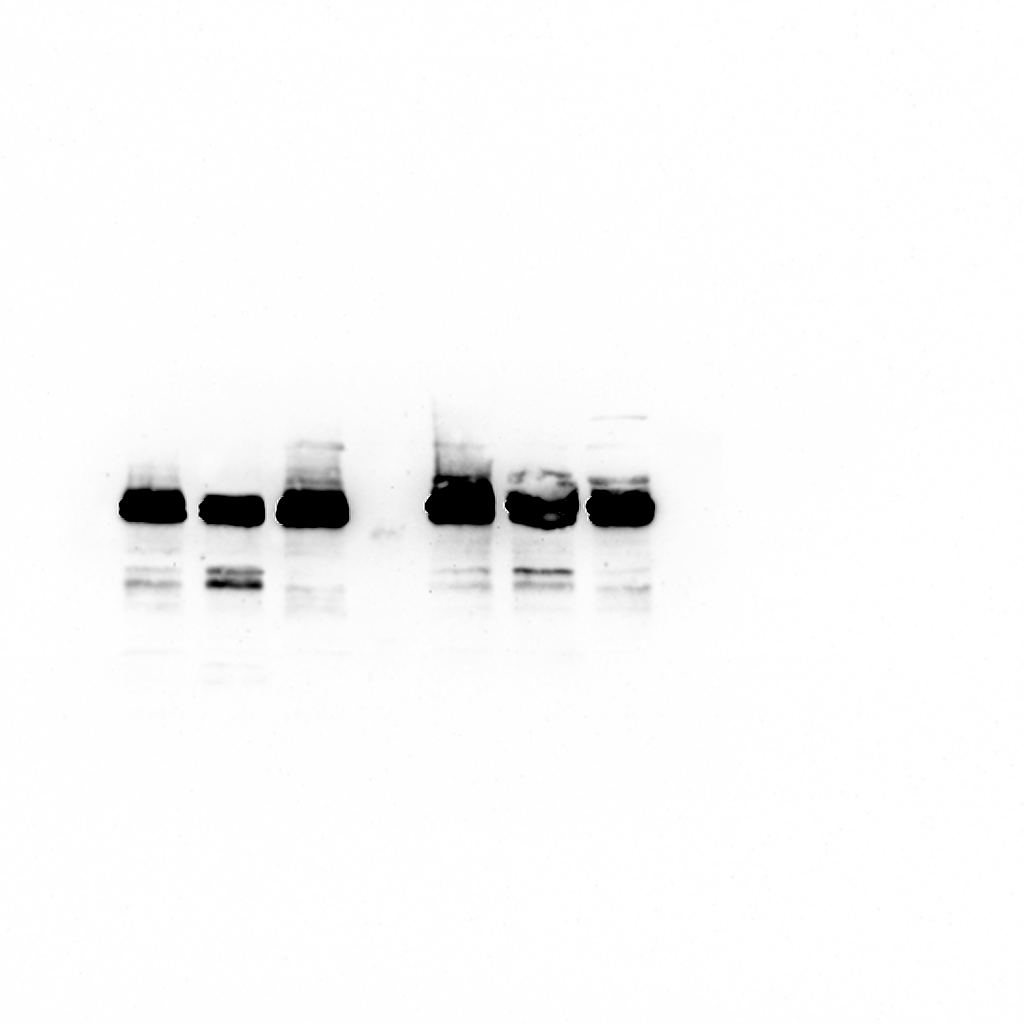

Supplement: Supplementary file 1 [file DataSheet_1.zip › Original source data - Western blot-Revised/Figure 2E/Cleaved Cas-3 Repeat-1 U87 4-5 lane, U251 1-2 lane.tif]

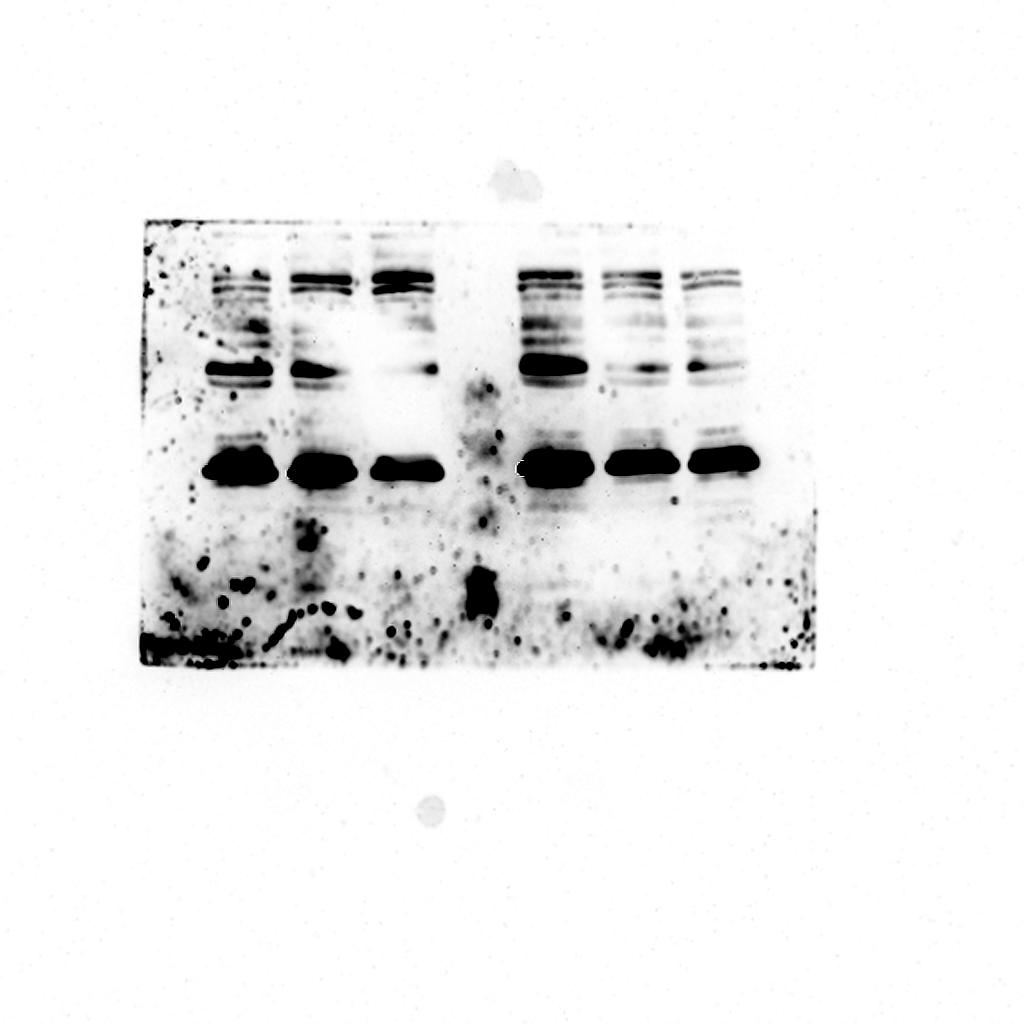

Supplement: Supplementary file 1 [file DataSheet_1.zip › Original source data - Western blot-Revised/Figure 2E/Cleaved Cas-3 Repeat-2 U251 1-2 lane.tif]

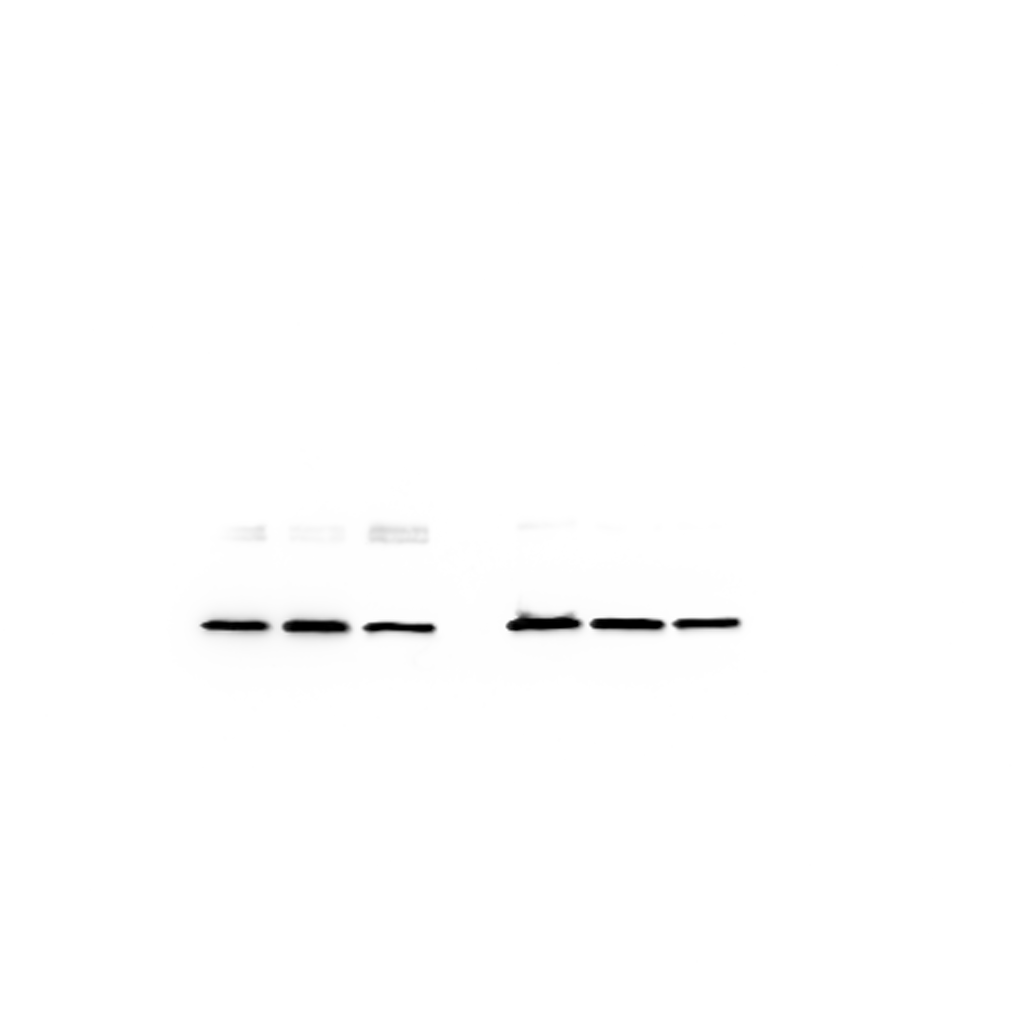

Supplement: Supplementary file 1 [file DataSheet_1.zip › Original source data - Western blot-Revised/Figure 2E/GAPDH Repeat-1 U87 4-5 lane, U251 1-2 lane.tif]

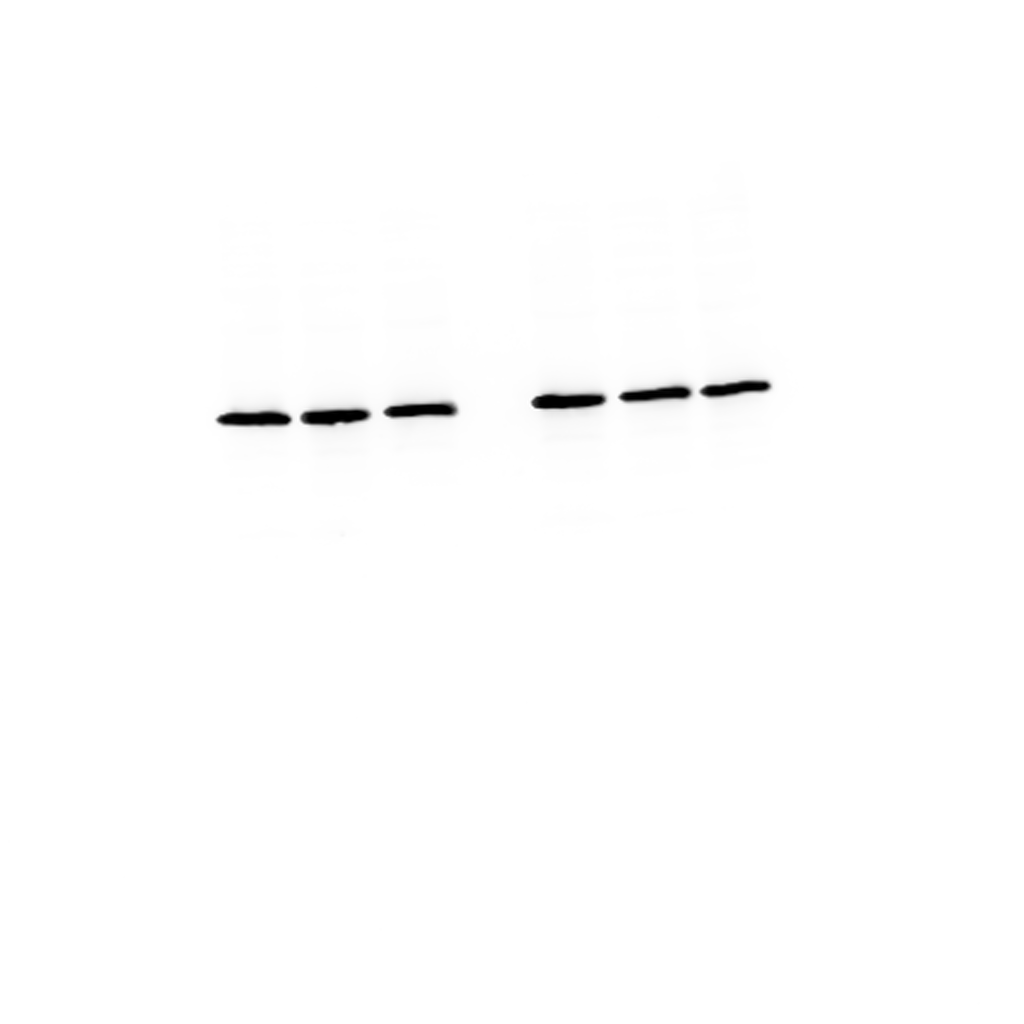

Supplement: Supplementary file 1 [file DataSheet_1.zip › Original source data - Western blot-Revised/Figure 2E/GAPDH Repeat-2 U251 1-2 lane.tif]

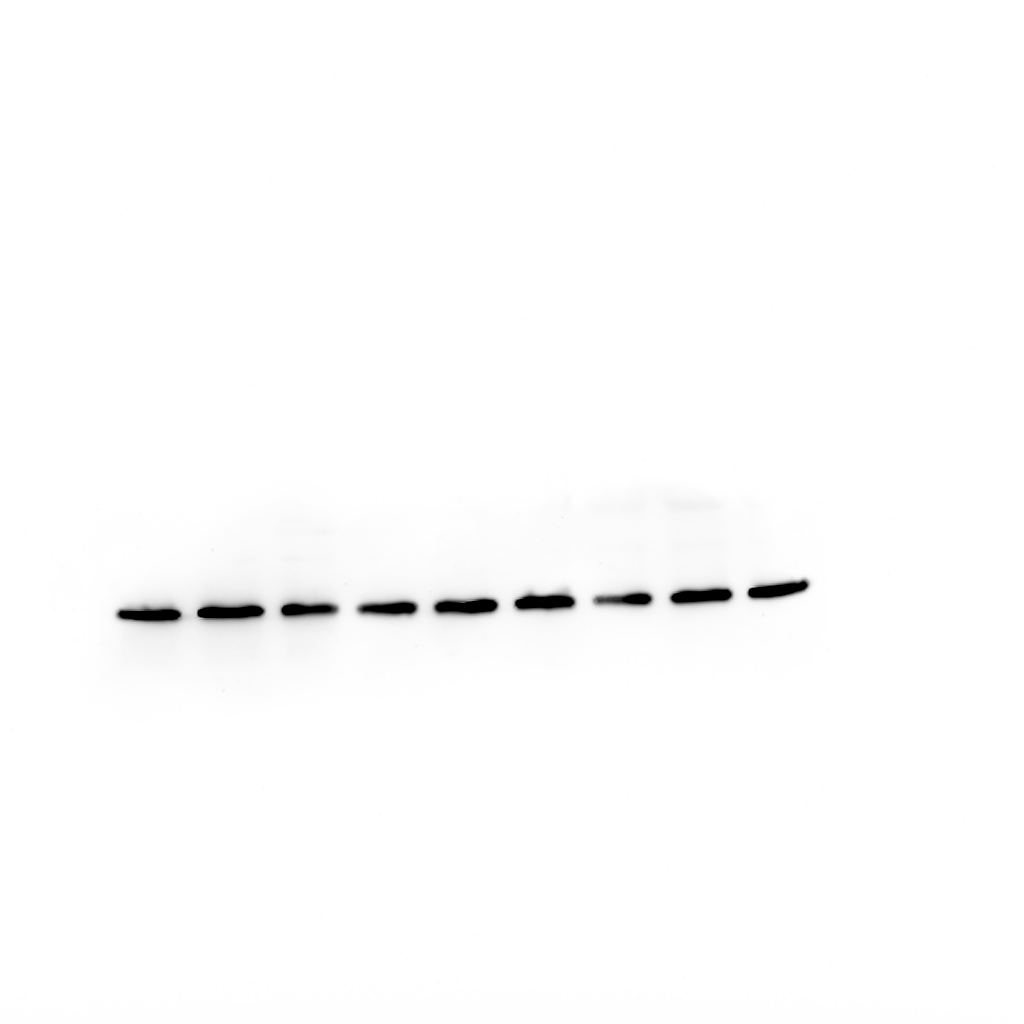

Supplement: Supplementary file 1 [file DataSheet_1.zip › Original source data - Western blot-Revised/Figure 2E/GAPDH Repeat-2 U87 4-5 lane.tif]

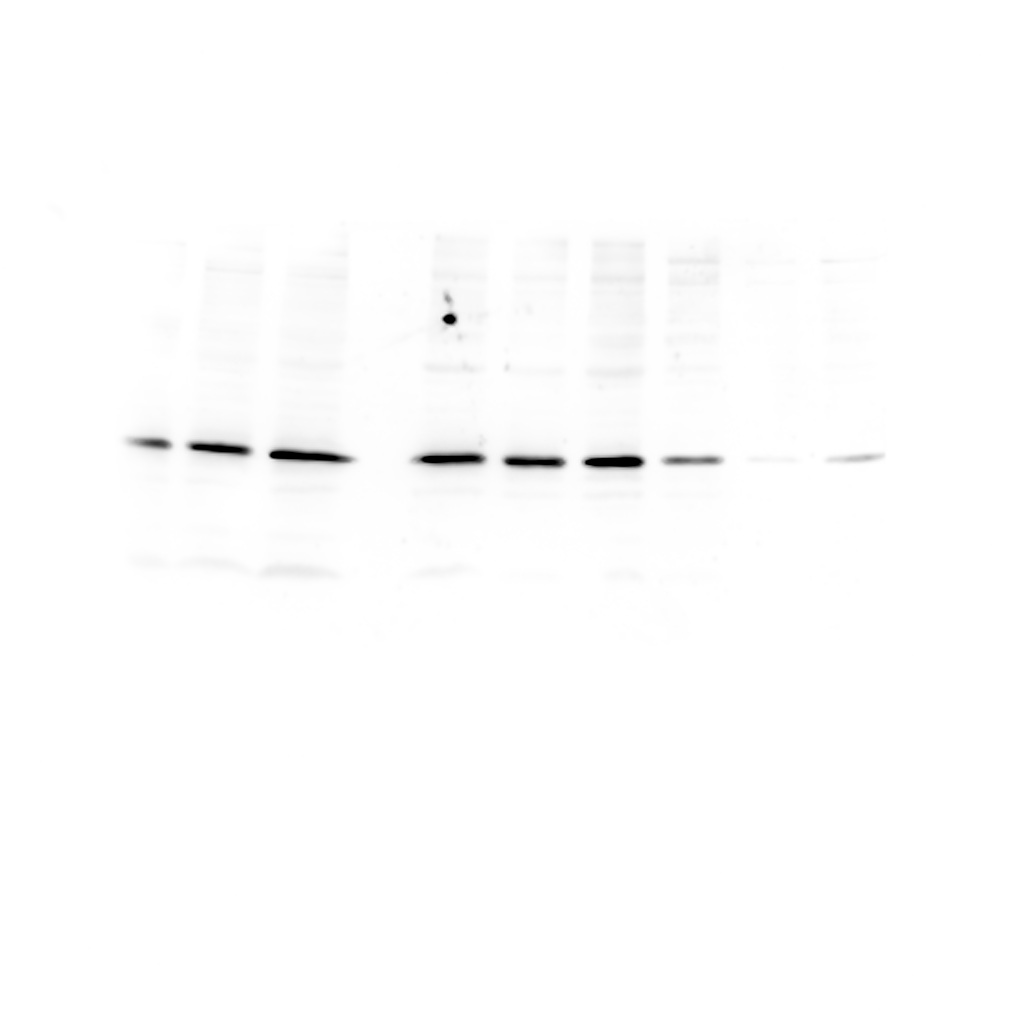

Supplement: Supplementary file 1 [file DataSheet_1.zip › Original source data - Western blot-Revised/Figure 2E/GAPDH Repeat-3 U251 2-3 lane, U87 4-5 lane.tif]

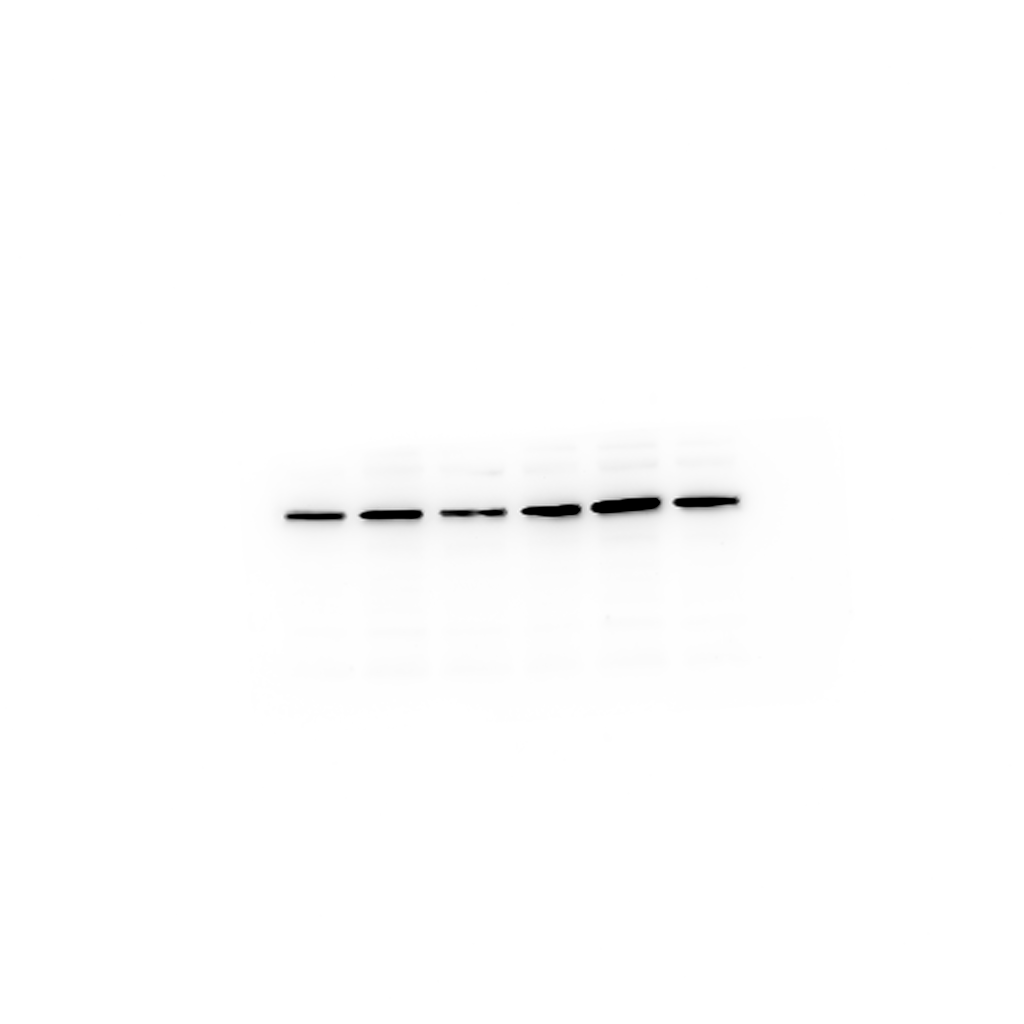

Supplement: Supplementary file 1 [file DataSheet_1.zip › Original source data - Western blot-Revised/Figure 4D/U251 Repeat-1 GAPDH 4-5 lane.tif]

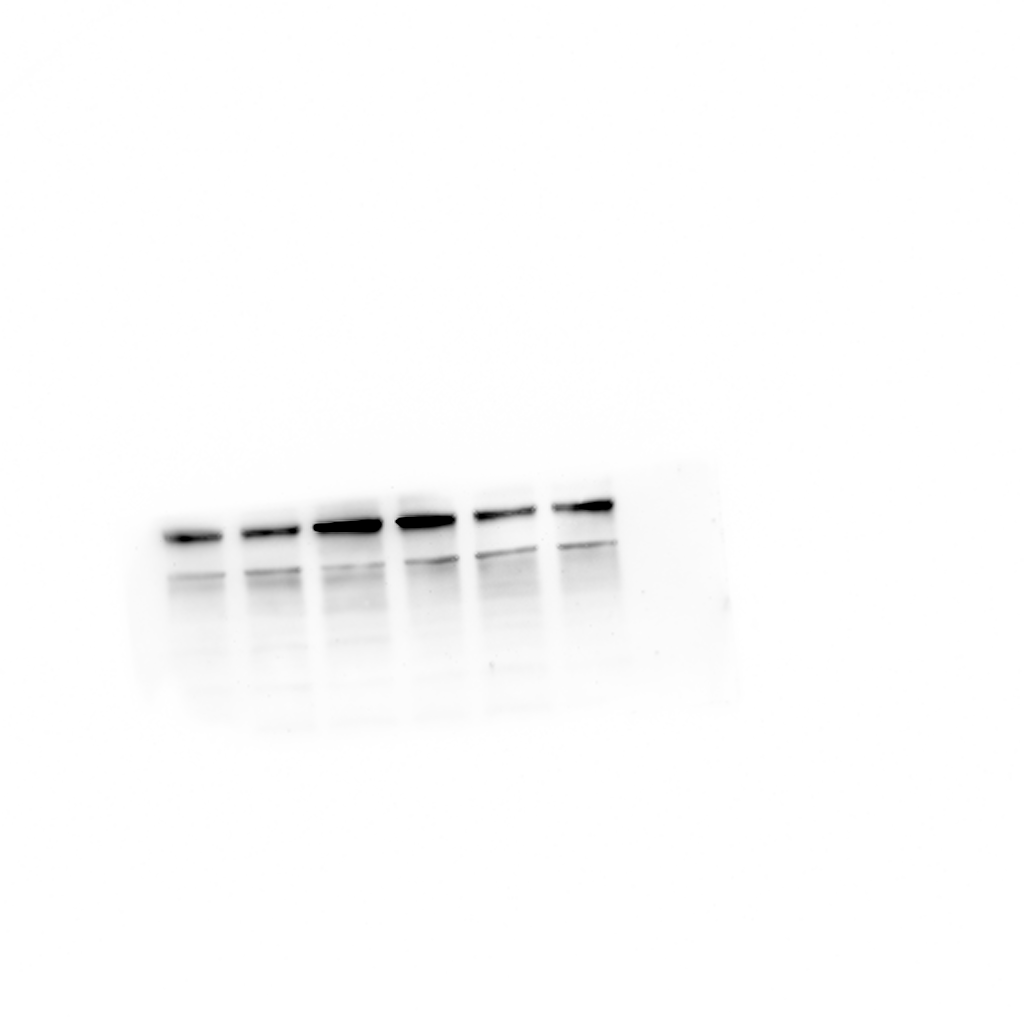

Supplement: Supplementary file 1 [file DataSheet_1.zip › Original source data - Western blot-Revised/Figure 4D/U251 Repeat-1 XIAP 4-5 lane.tif]

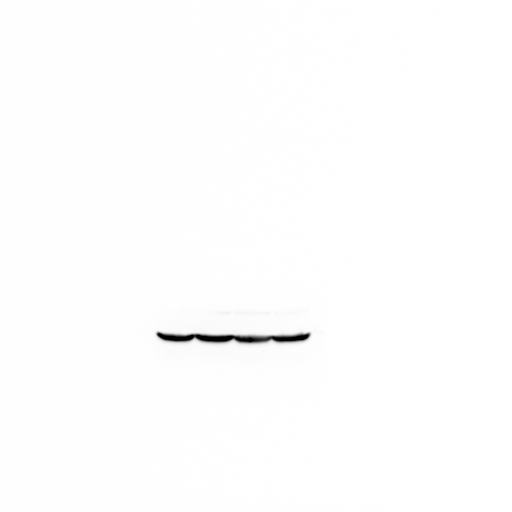

Supplement: Supplementary file 1 [file DataSheet_1.zip › Original source data - Western blot-Revised/Figure 4D/U251 Repeat-2 GAPDH 3-4 lane.tif]

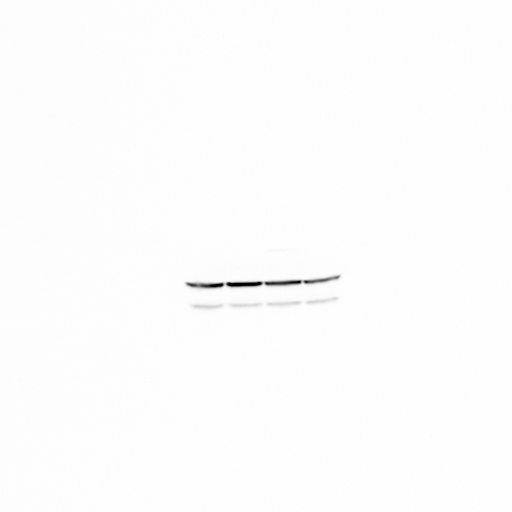

Supplement: Supplementary file 1 [file DataSheet_1.zip › Original source data - Western blot-Revised/Figure 4D/U251 Repeat-2 XIAP 3-4 lane.tif]

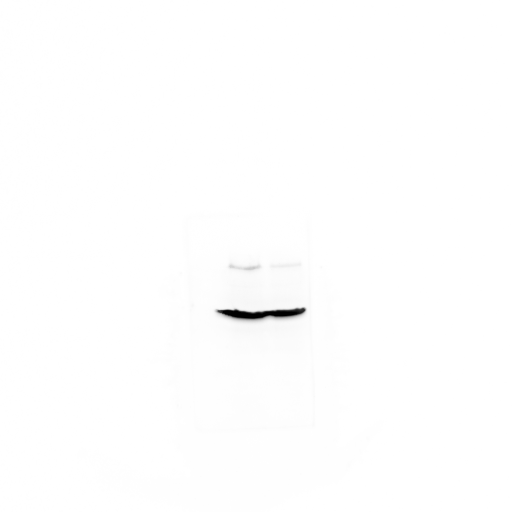

Supplement: Supplementary file 1 [file DataSheet_1.zip › Original source data - Western blot-Revised/Figure 4D/U251 Repeat-3 GAPDH 1-2 lane.tif]

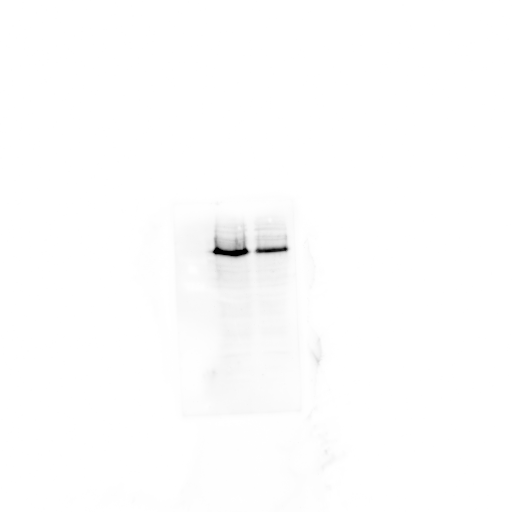

Supplement: Supplementary file 1 [file DataSheet_1.zip › Original source data - Western blot-Revised/Figure 4D/U251 Repeat-3 XIAP 1-2 lane.tif]

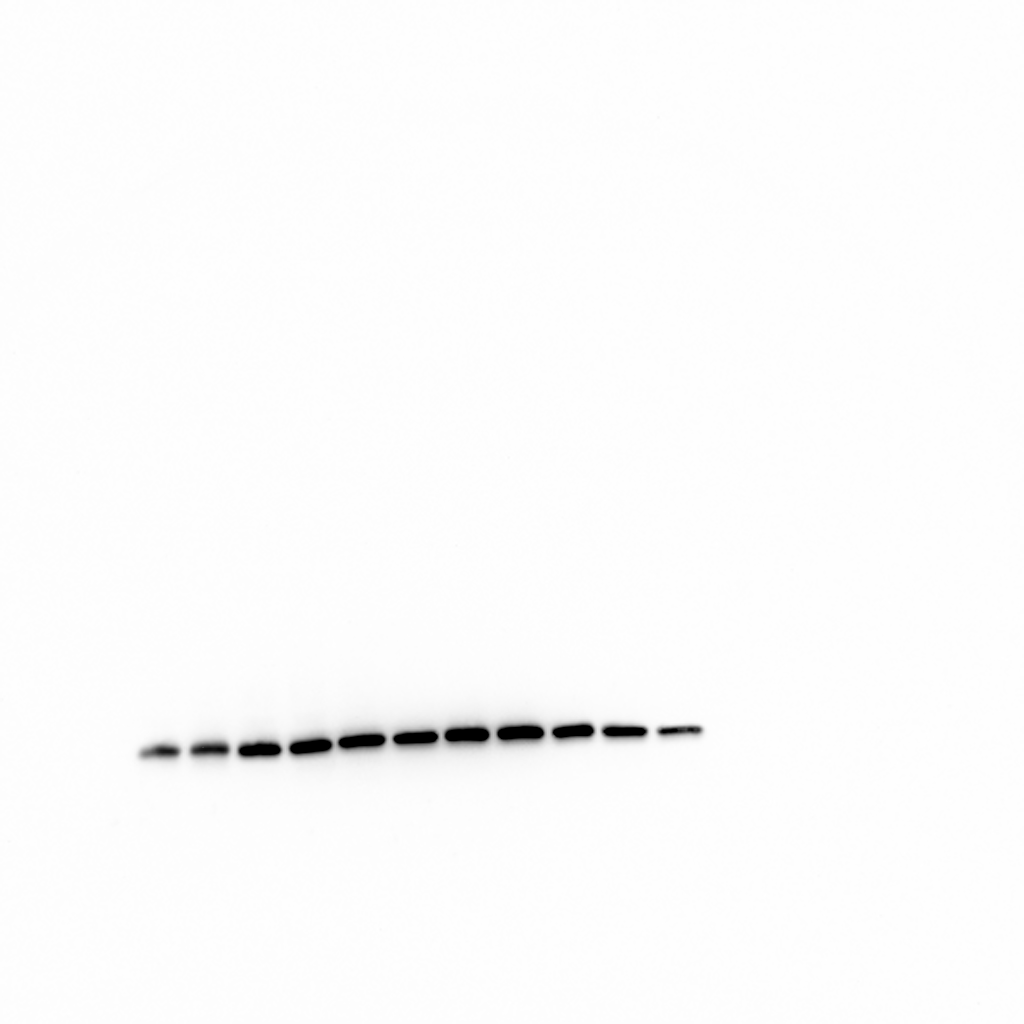

Supplement: Supplementary file 1 [file DataSheet_1.zip › Original source data - Western blot-Revised/Figure 4D/U87 Repeat-1 GAPDH 1-2 lane.tif]

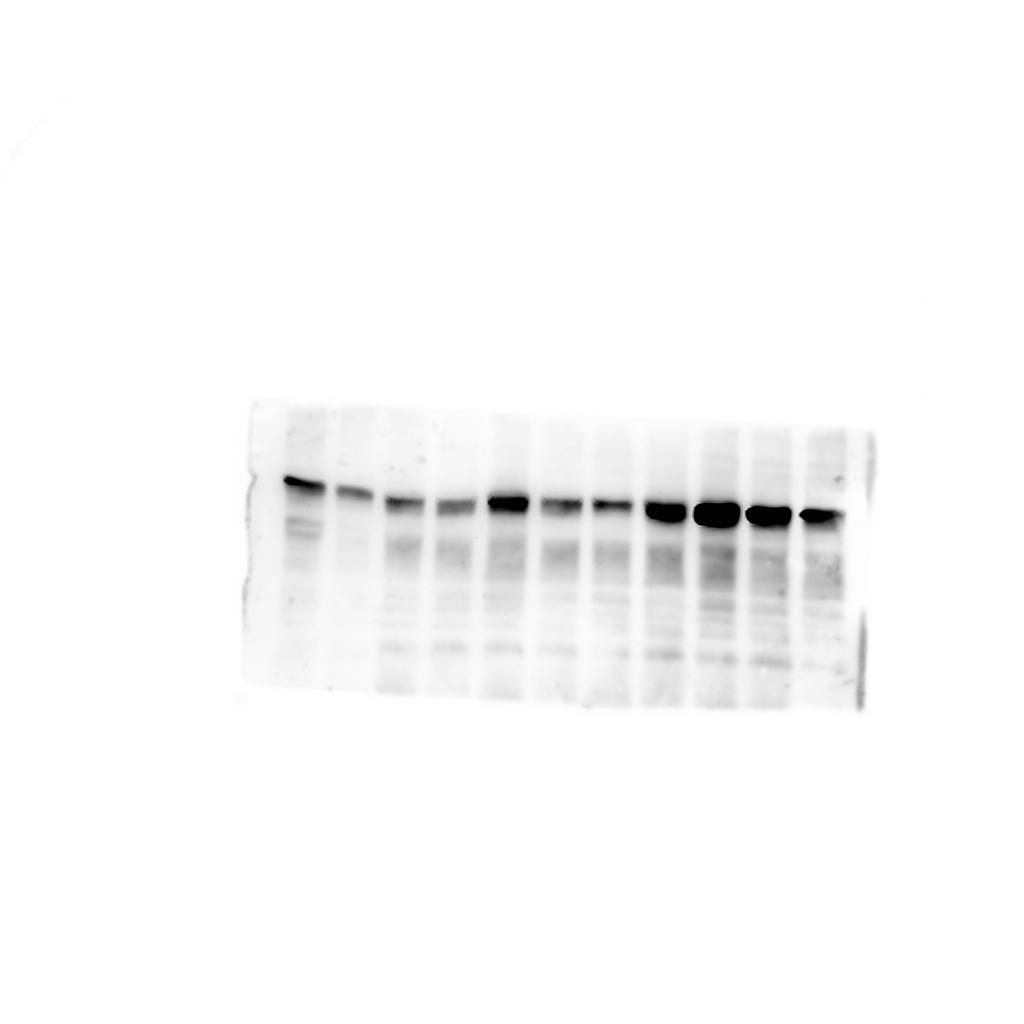

Supplement: Supplementary file 1 [file DataSheet_1.zip › Original source data - Western blot-Revised/Figure 4D/U87 Repeat-1 XIAP 1-2 lane.tif]

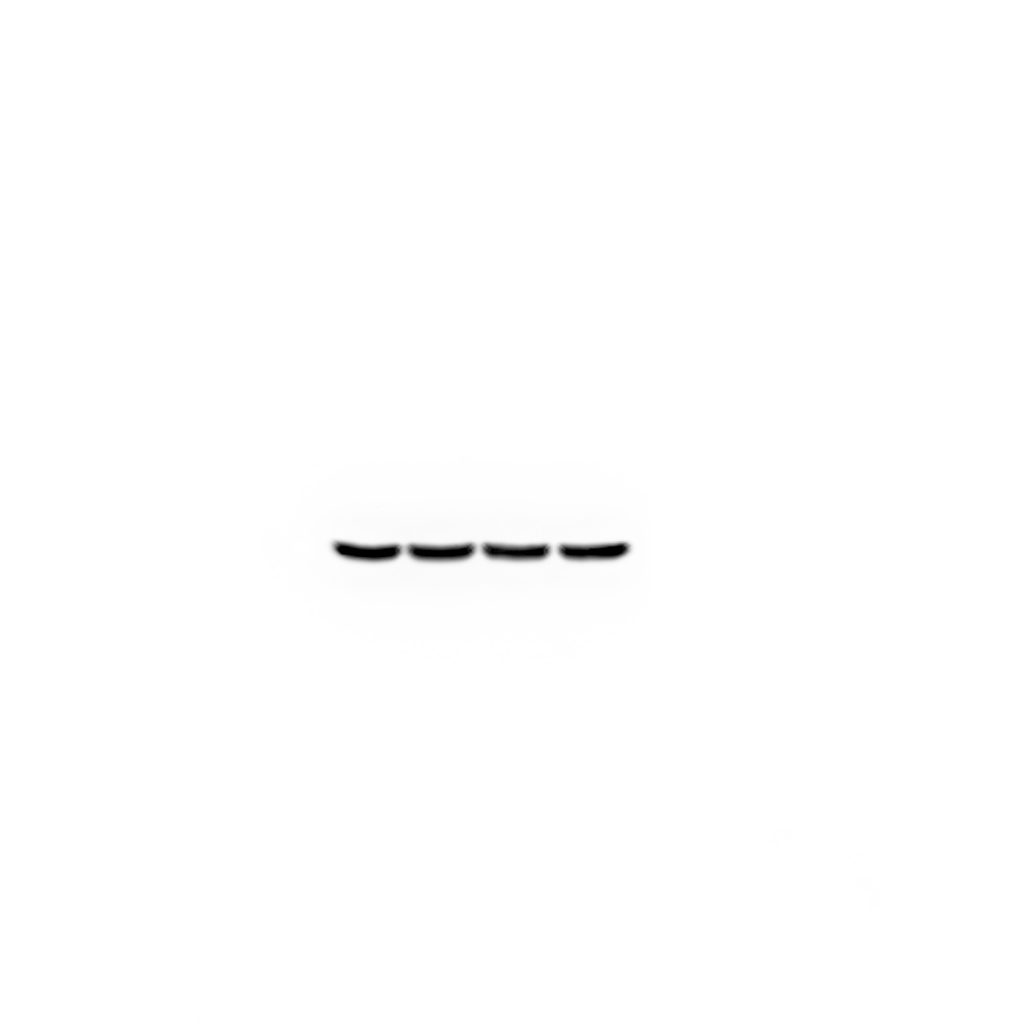

Supplement: Supplementary file 1 [file DataSheet_1.zip › Original source data - Western blot-Revised/Figure 4D/U87 Repeat-2 GAPDH 1-2 lane.tif]

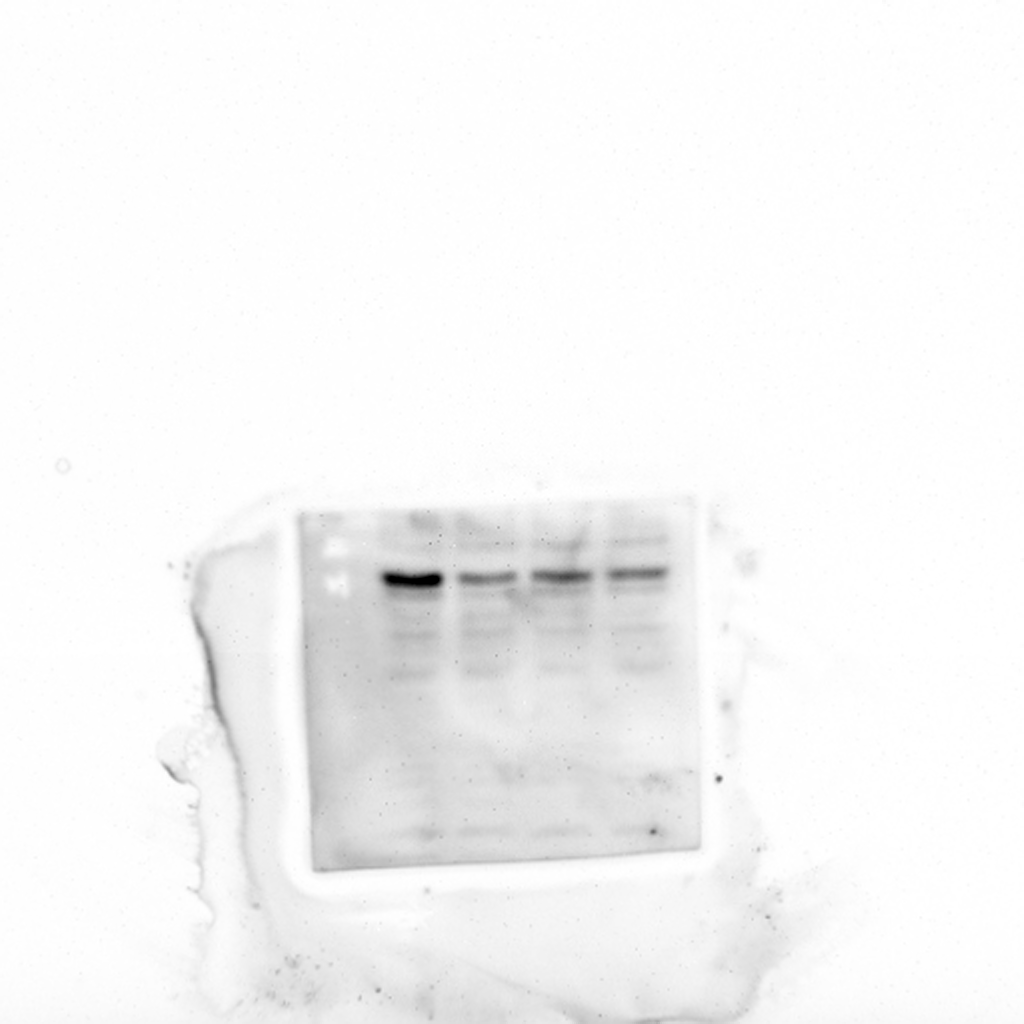

Supplement: Supplementary file 1 [file DataSheet_1.zip › Original source data - Western blot-Revised/Figure 4D/U87 Repeat-2 XIAP 1-2 lane.tif]

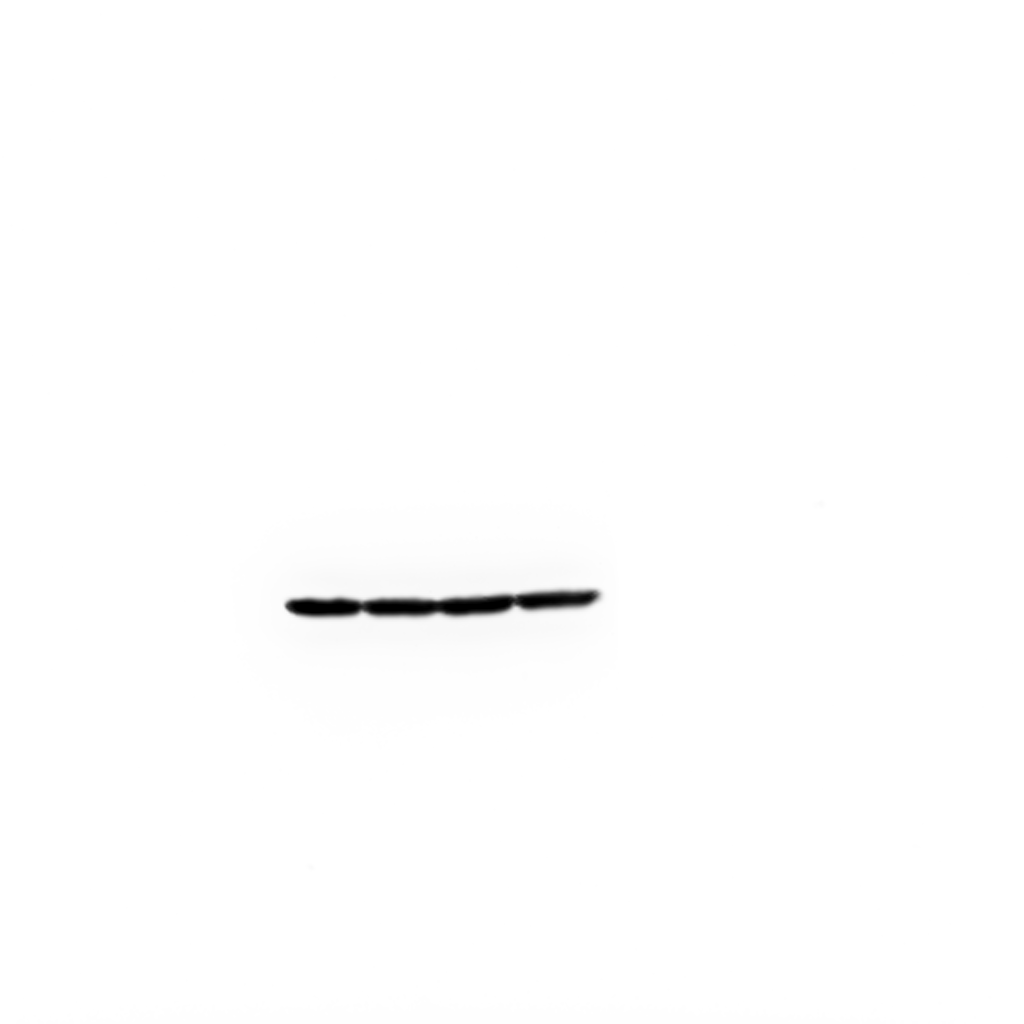

Supplement: Supplementary file 1 [file DataSheet_1.zip › Original source data - Western blot-Revised/Figure 4D/U87 Repeat-3 GAPDH 1-2 lane.tif]

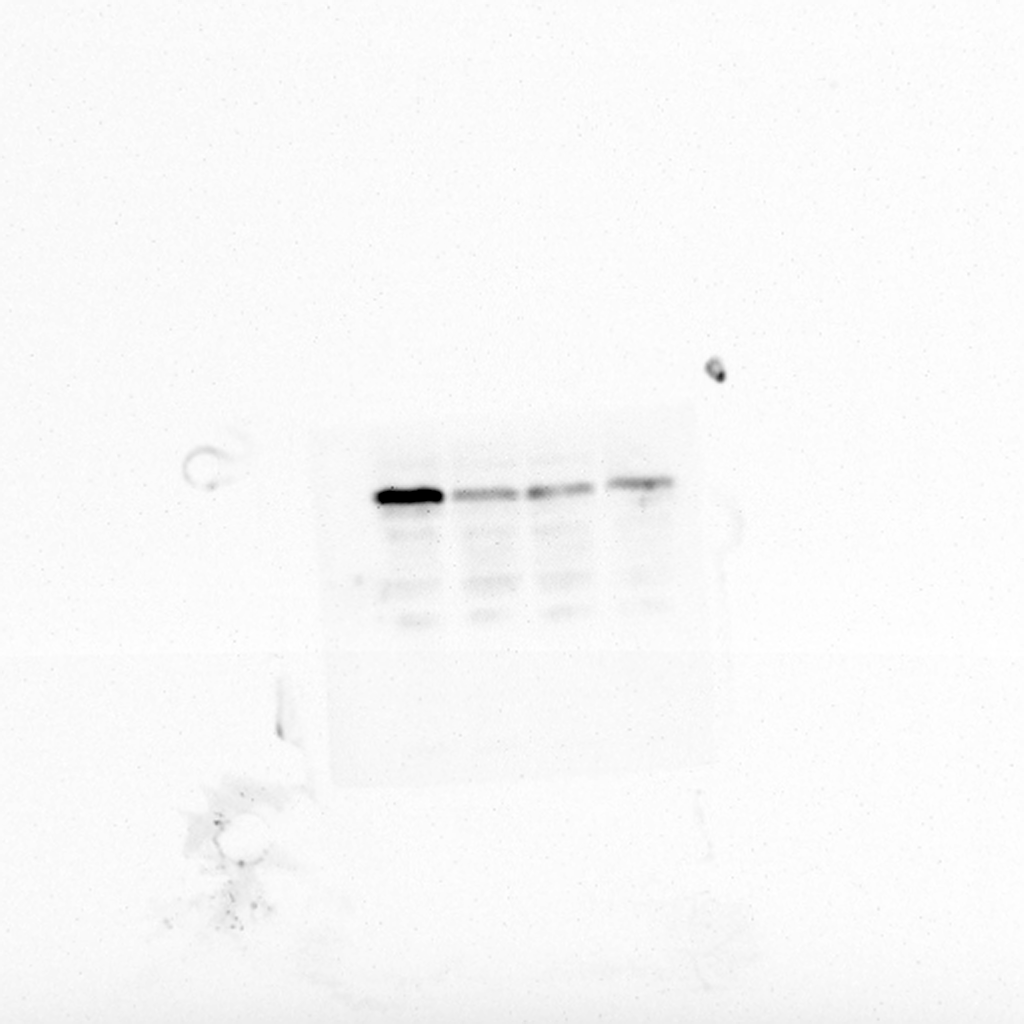

Supplement: Supplementary file 1 [file DataSheet_1.zip › Original source data - Western blot-Revised/Figure 4D/U87 Repeat-3 XIAP 1-2 lane.tif]

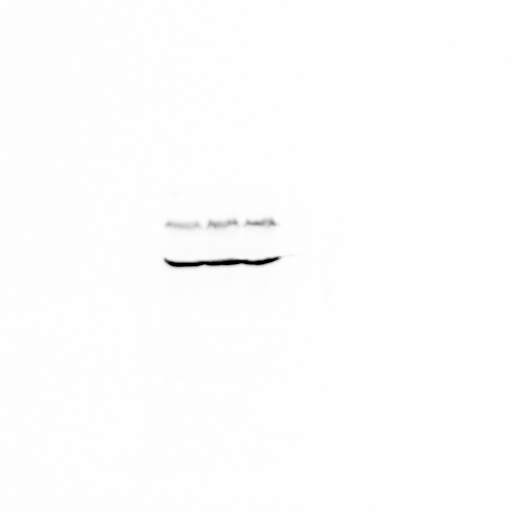

Supplement: Supplementary file 1 [file DataSheet_1.zip › Original source data - Western blot-Revised/Figure 4F/U251 Repeat-1 GAPDH 1-2 lane.tif]

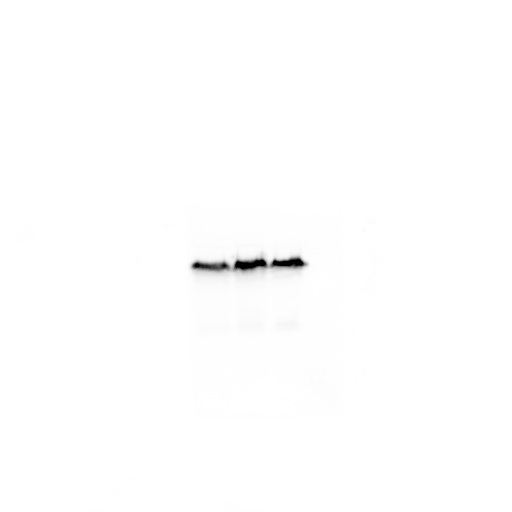

Supplement: Supplementary file 1 [file DataSheet_1.zip › Original source data - Western blot-Revised/Figure 4F/U251 Repeat-1 XIAP 1-2 lane.tif]

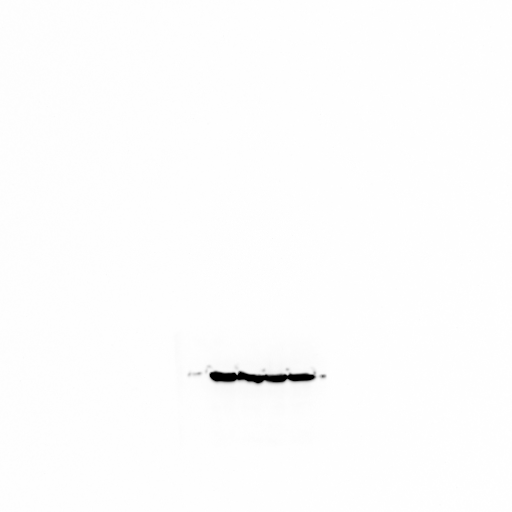

Supplement: Supplementary file 1 [file DataSheet_1.zip › Original source data - Western blot-Revised/Figure 4F/U251 Repeat-3 GAPDH 3-4 lane.tif]

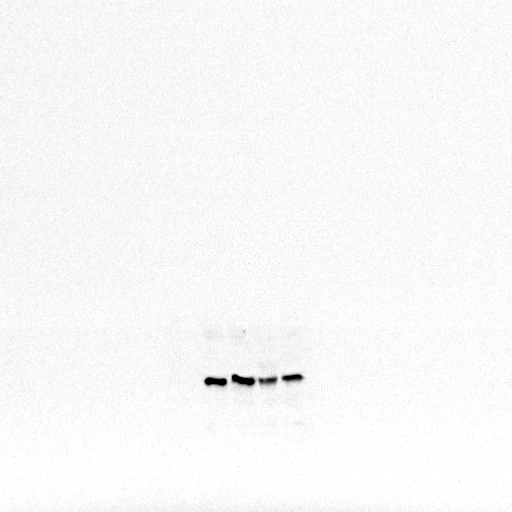

Supplement: Supplementary file 1 [file DataSheet_1.zip › Original source data - Western blot-Revised/Figure 4F/U251 Repeat-3 XIAP 3-4 lane.tif]

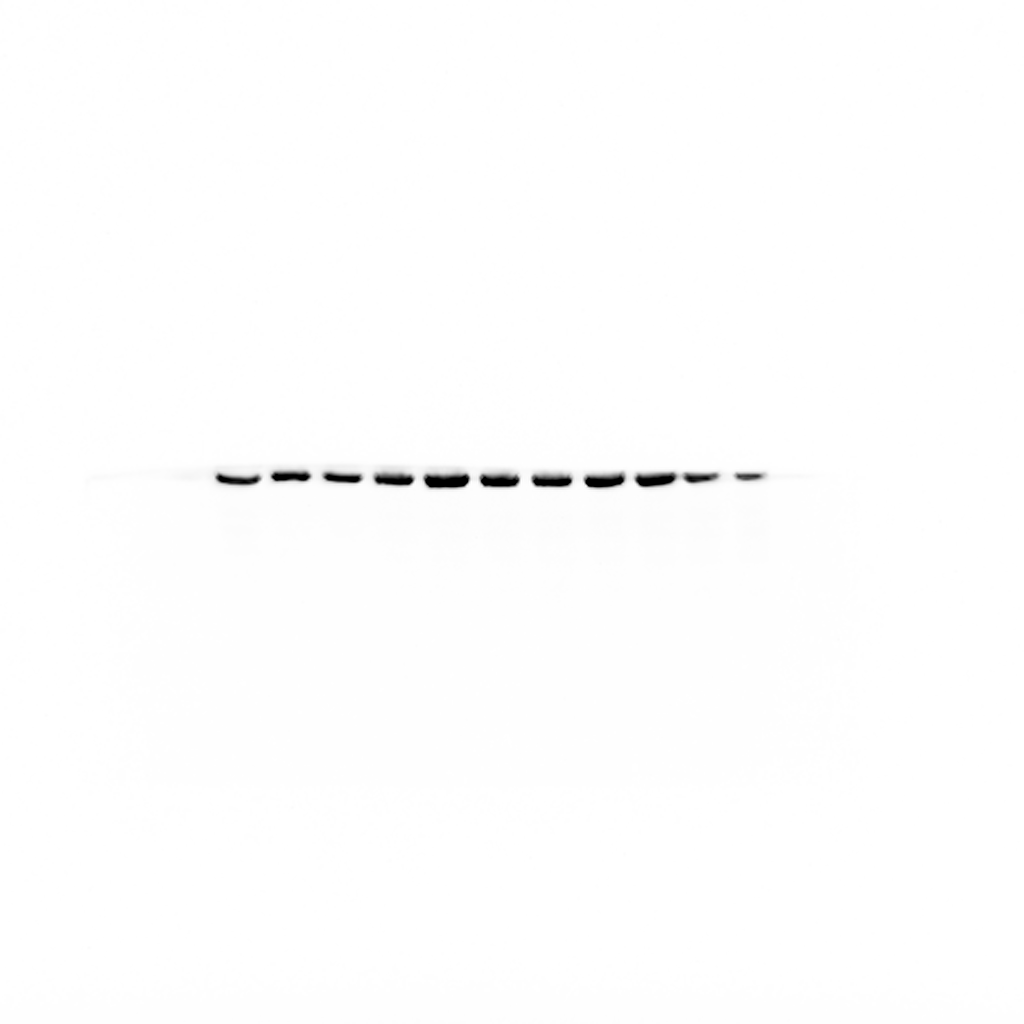

Supplement: Supplementary file 1 [file DataSheet_1.zip › Original source data - Western blot-Revised/Figure 4F/U87 Repeat-1 GAPDH 3-4 lane.tif]

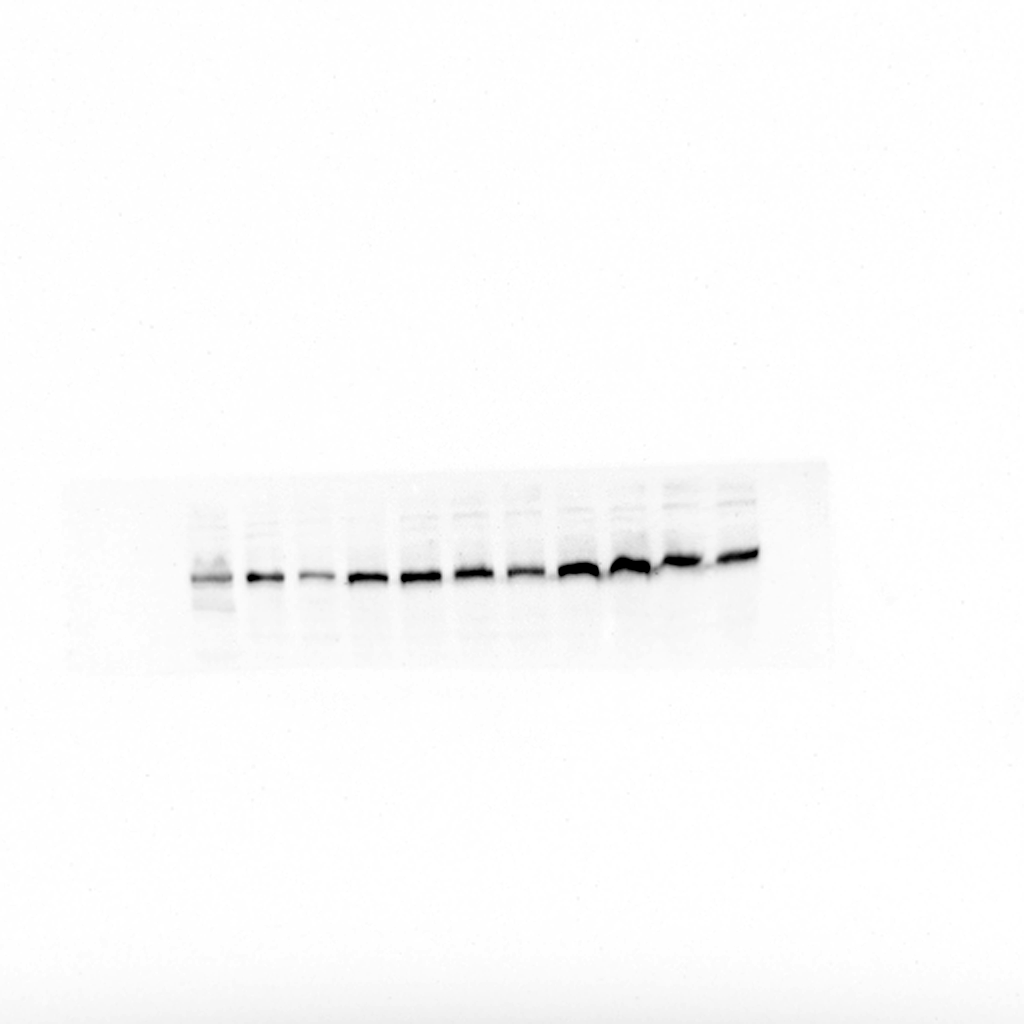

Supplement: Supplementary file 1 [file DataSheet_1.zip › Original source data - Western blot-Revised/Figure 4F/U87 Repeat-1 XIAP 3-4 lane.tif]

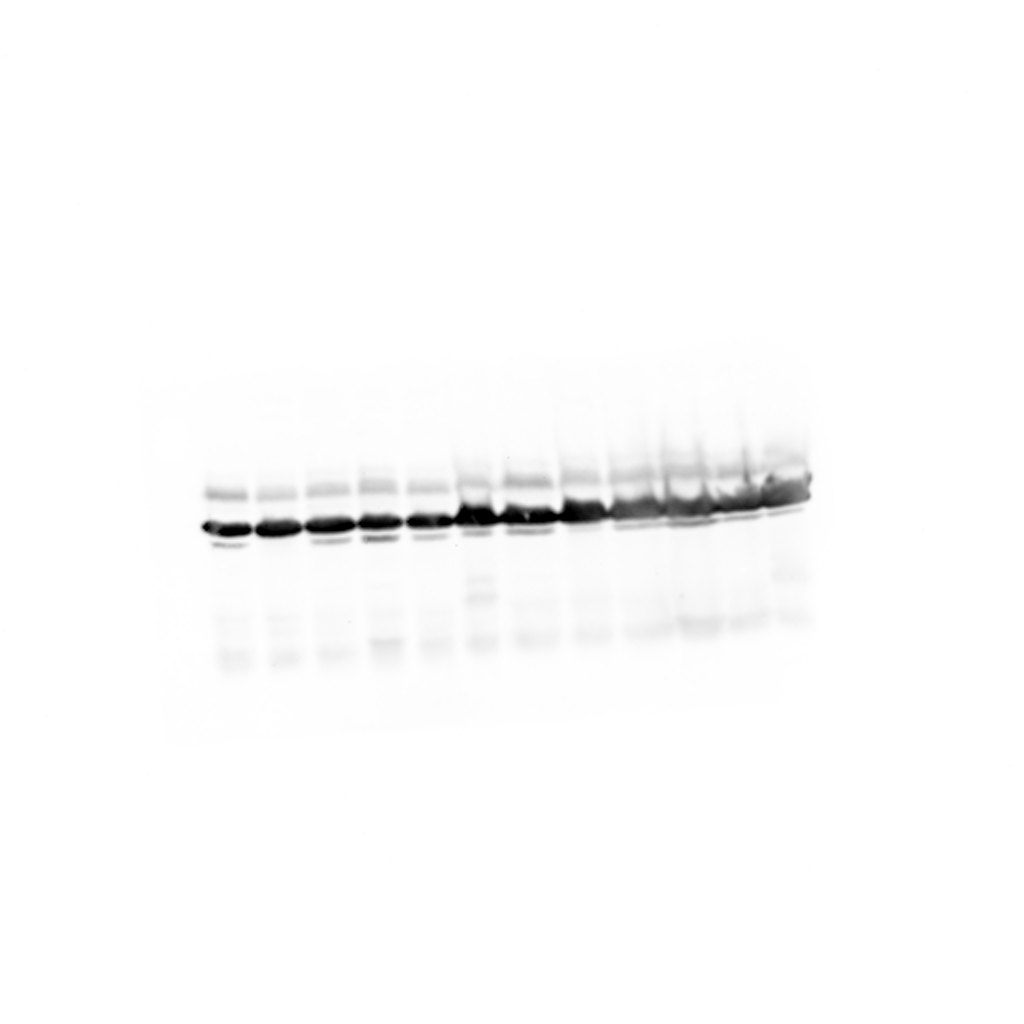

Supplement: Supplementary file 1 [file DataSheet_1.zip › Original source data - Western blot-Revised/Figure 4F/U87 Repeat-2 GAPDH 2-3 lane.tif]

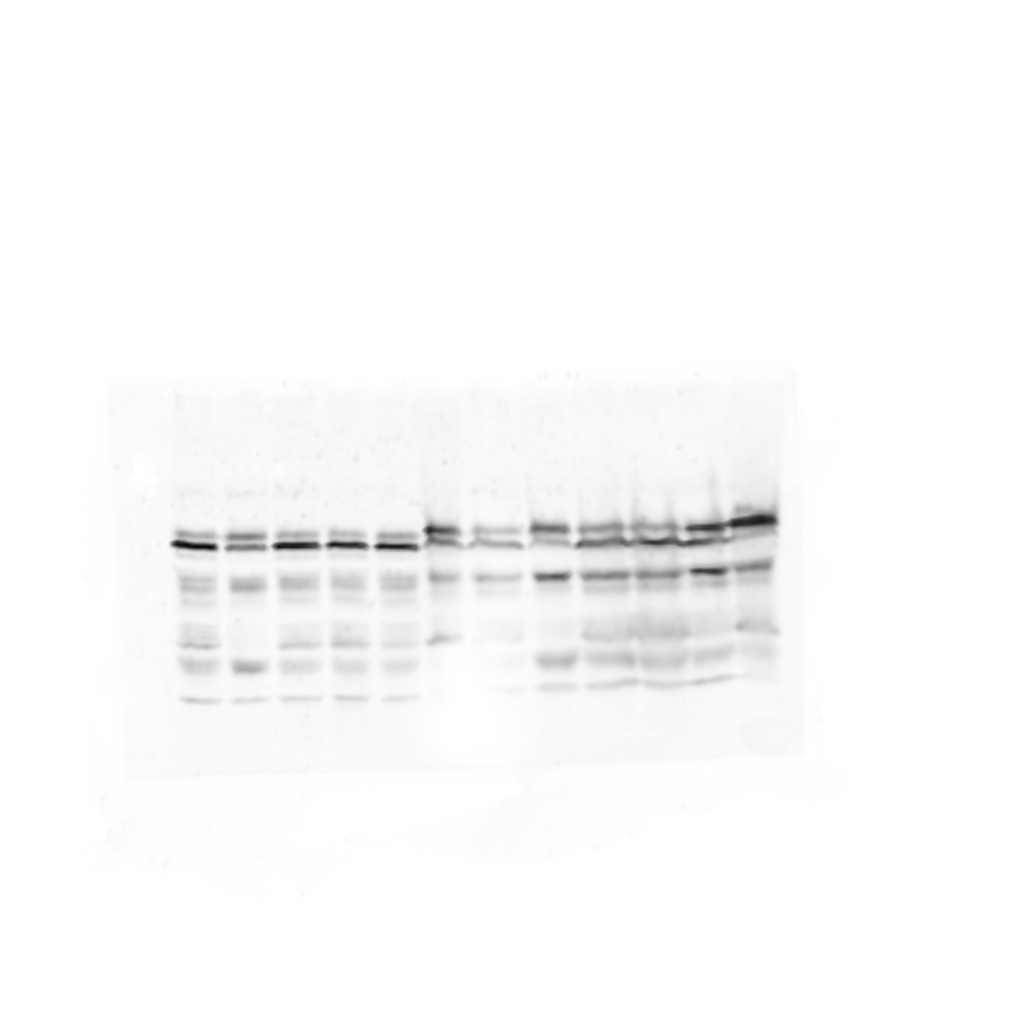

Supplement: Supplementary file 1 [file DataSheet_1.zip › Original source data - Western blot-Revised/Figure 4F/U87 Repeat-2 XIAP 2-3 lane.tif]

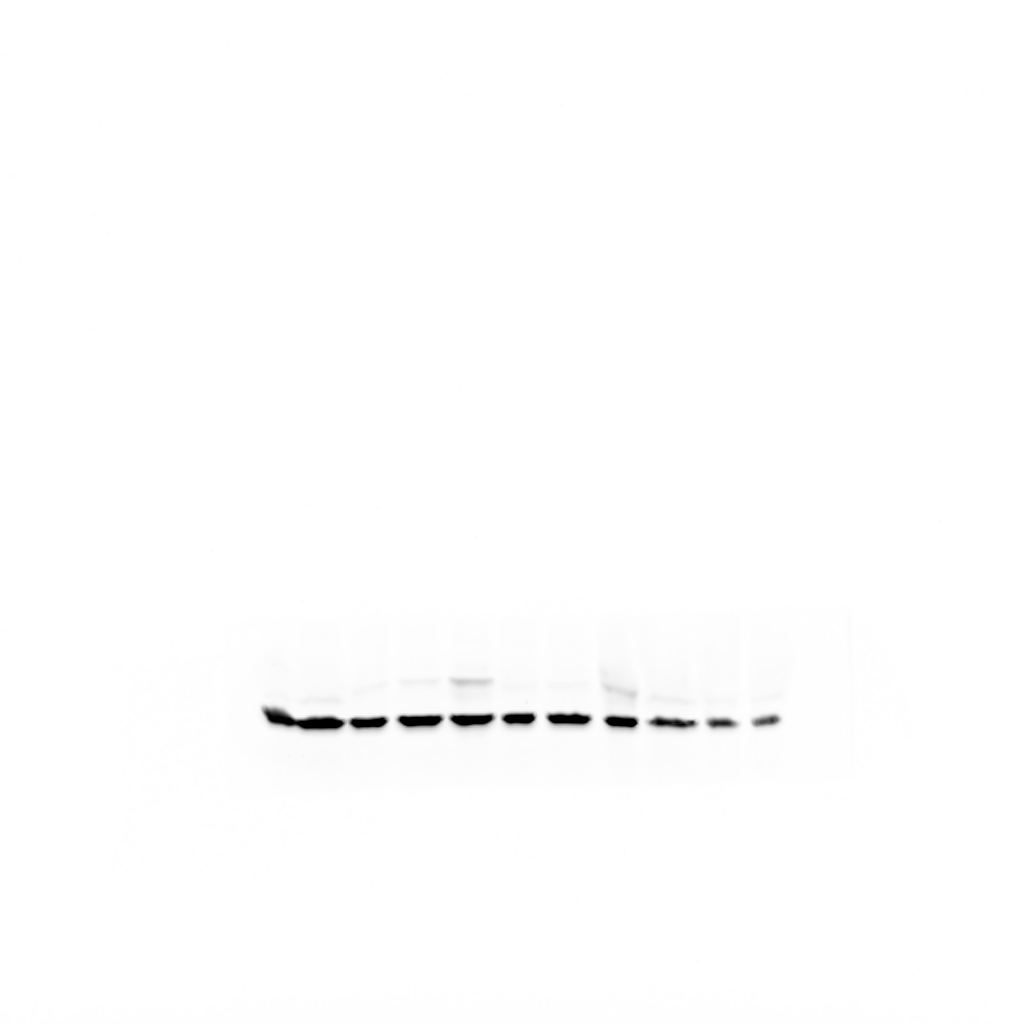

Supplement: Supplementary file 1 [file DataSheet_1.zip › Original source data - Western blot-Revised/Figure 4F/U87 Repeat-3 GAPDH 3-4 lane.tif]

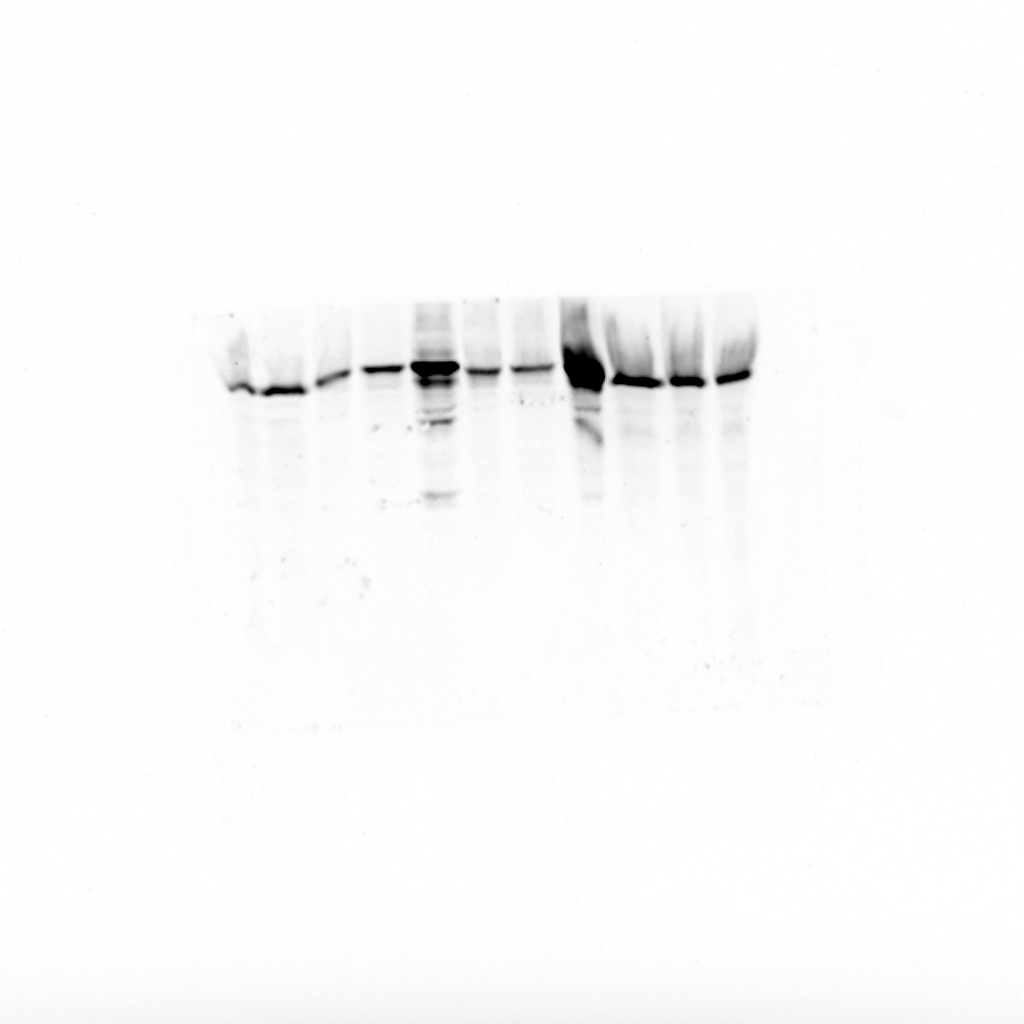

Supplement: Supplementary file 1 [file DataSheet_1.zip › Original source data - Western blot-Revised/Figure 4F/U87 Repeat-3 XIAP 3-4 lane.tif]

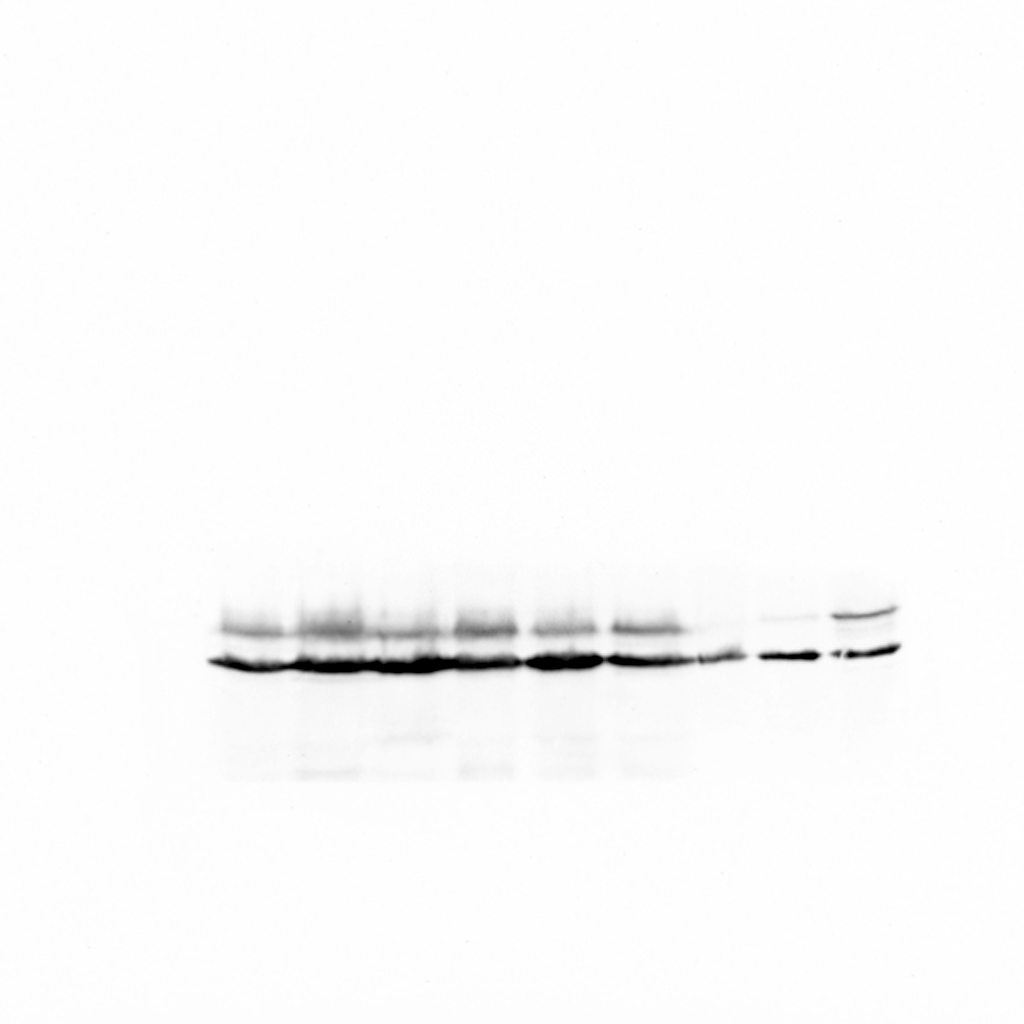

Supplement: Supplementary file 1 [file DataSheet_1.zip › Original source data - Western blot-Revised/Figure 6B/U251 Repeat-1 GAPDH 4-6 lane.tif]

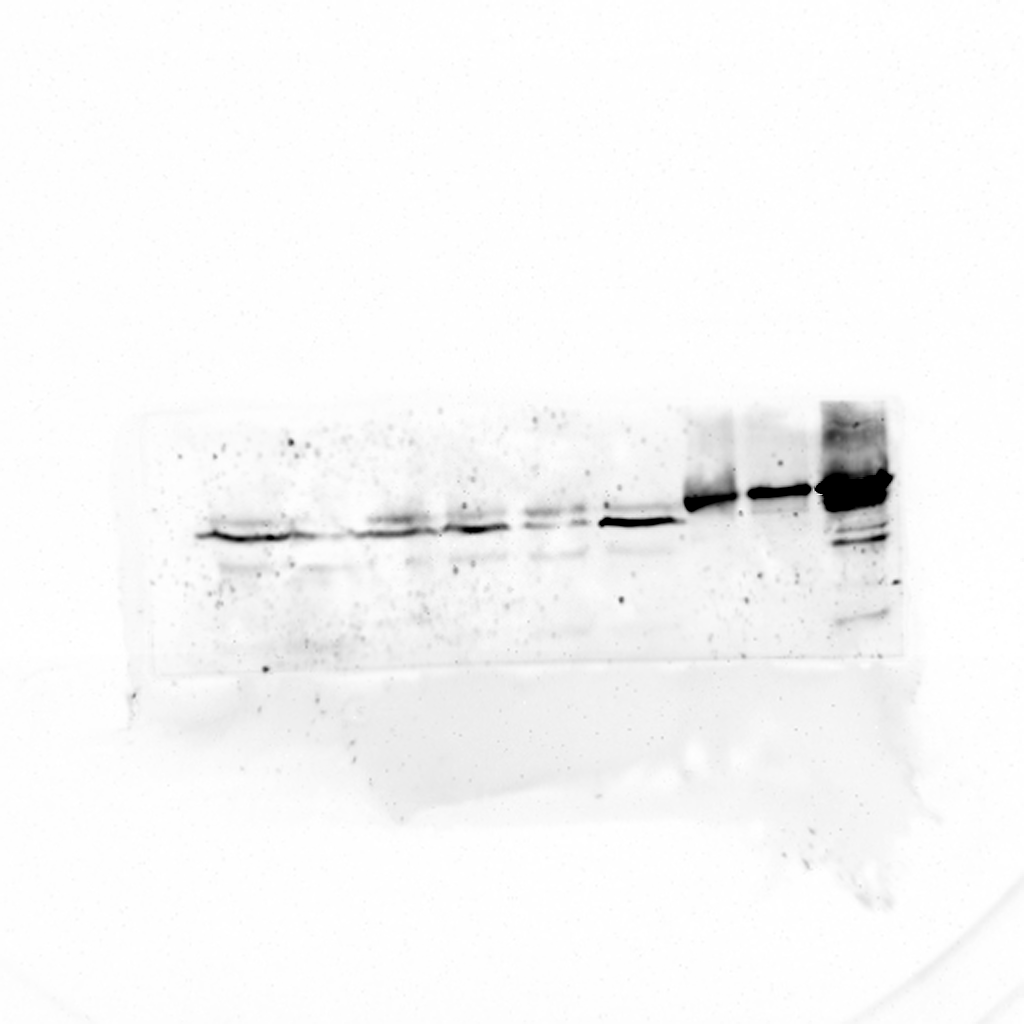

Supplement: Supplementary file 1 [file DataSheet_1.zip › Original source data - Western blot-Revised/Figure 6B/U251 Repeat-1 XIAP 4-6 lane.tif]

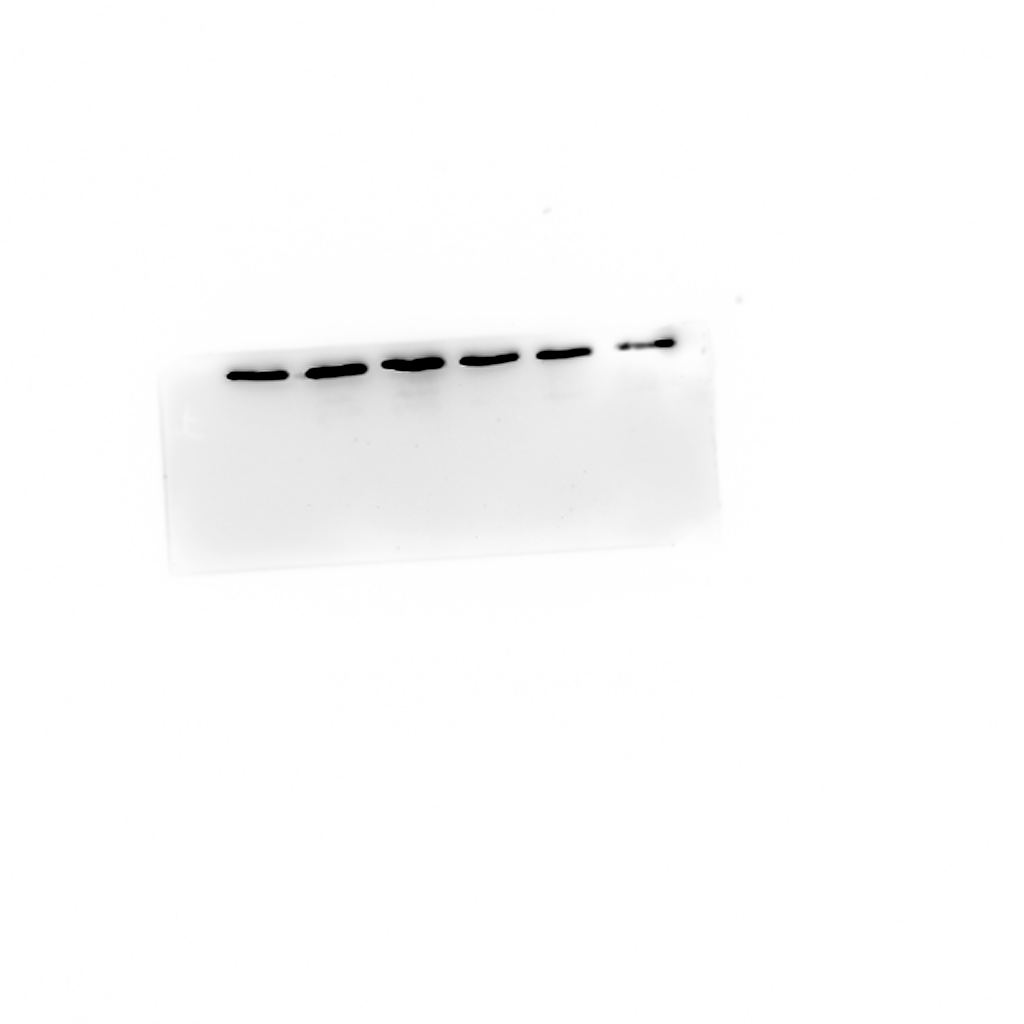

Supplement: Supplementary file 1 [file DataSheet_1.zip › Original source data - Western blot-Revised/Figure 6B/U251 Repeat-2 GAPDH 2-4 lane.tif]

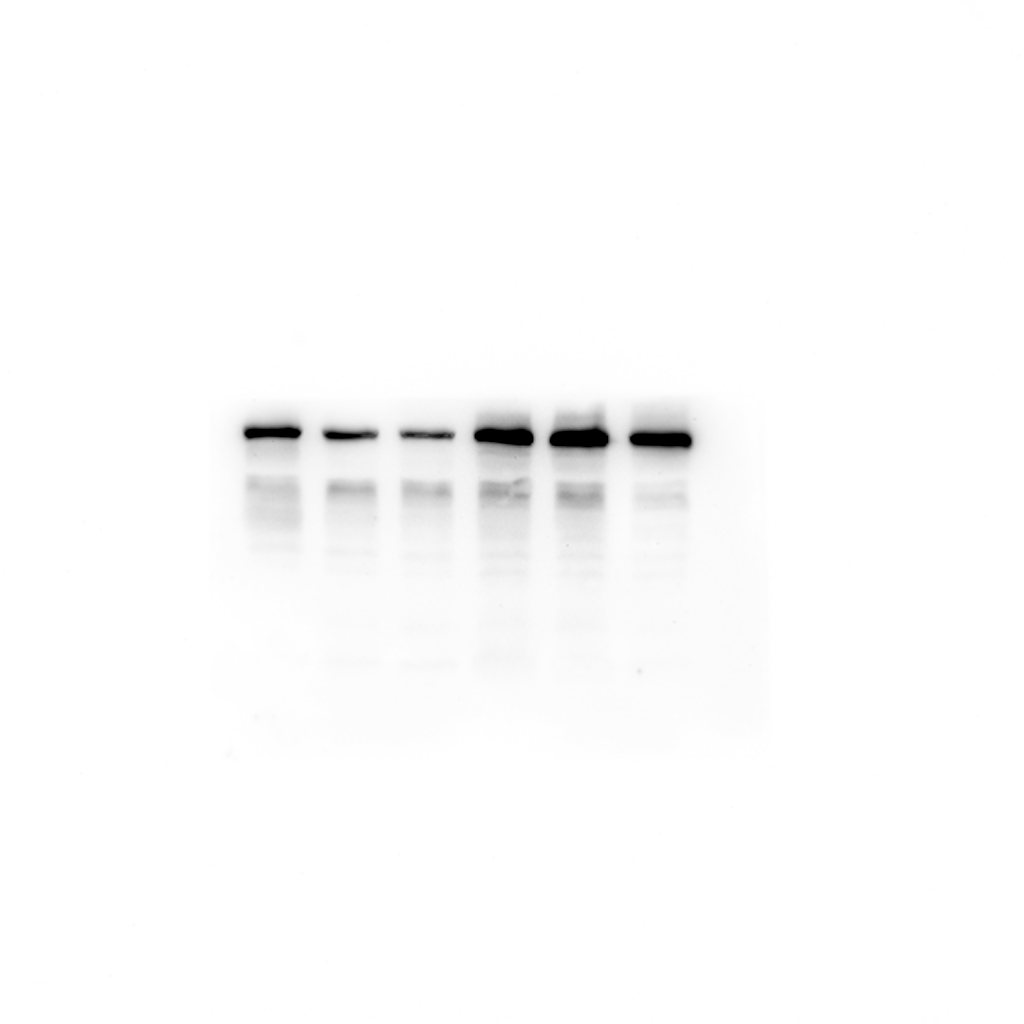

Supplement: Supplementary file 1 [file DataSheet_1.zip › Original source data - Western blot-Revised/Figure 6B/U251 Repeat-2 XIAP 2-4 lane.tif]

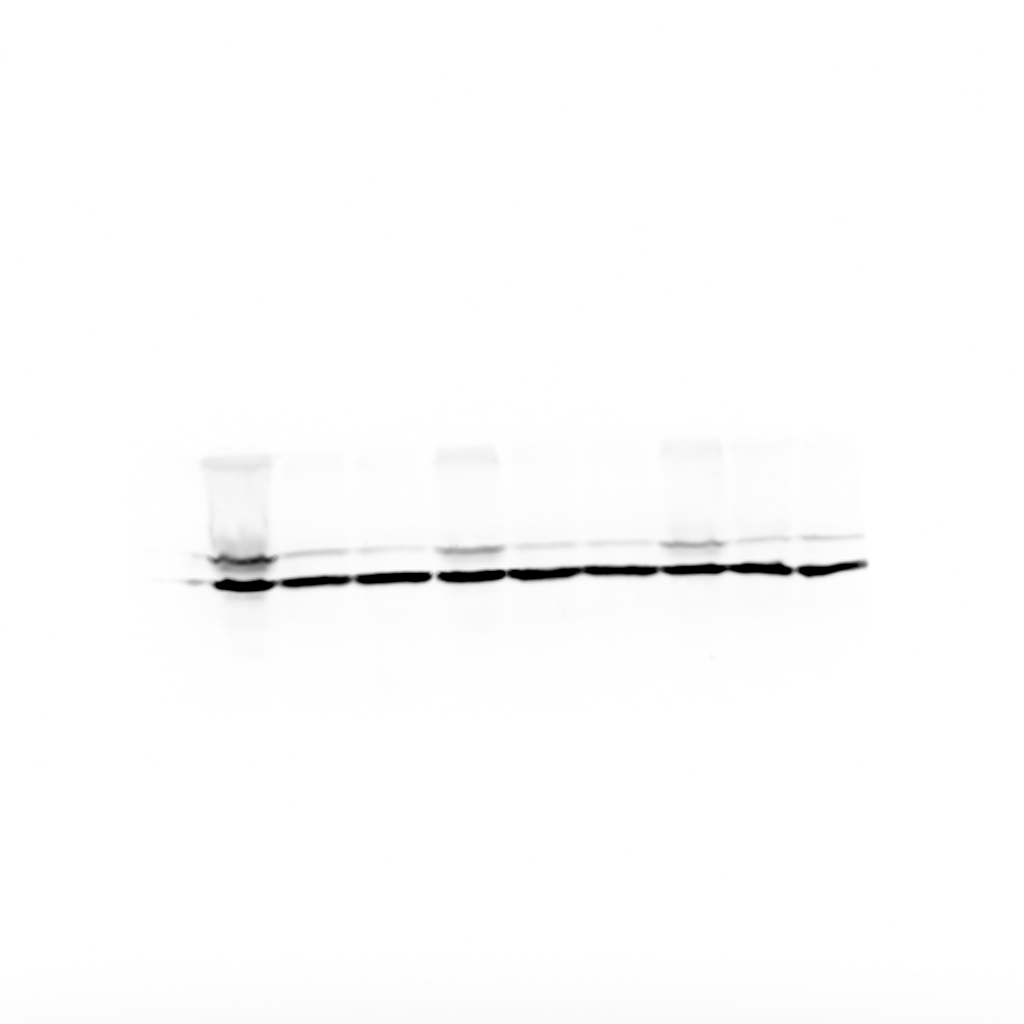

Supplement: Supplementary file 1 [file DataSheet_1.zip › Original source data - Western blot-Revised/Figure 6B/U251 Repeat-3 GAPDH 5-7 lane.tif]

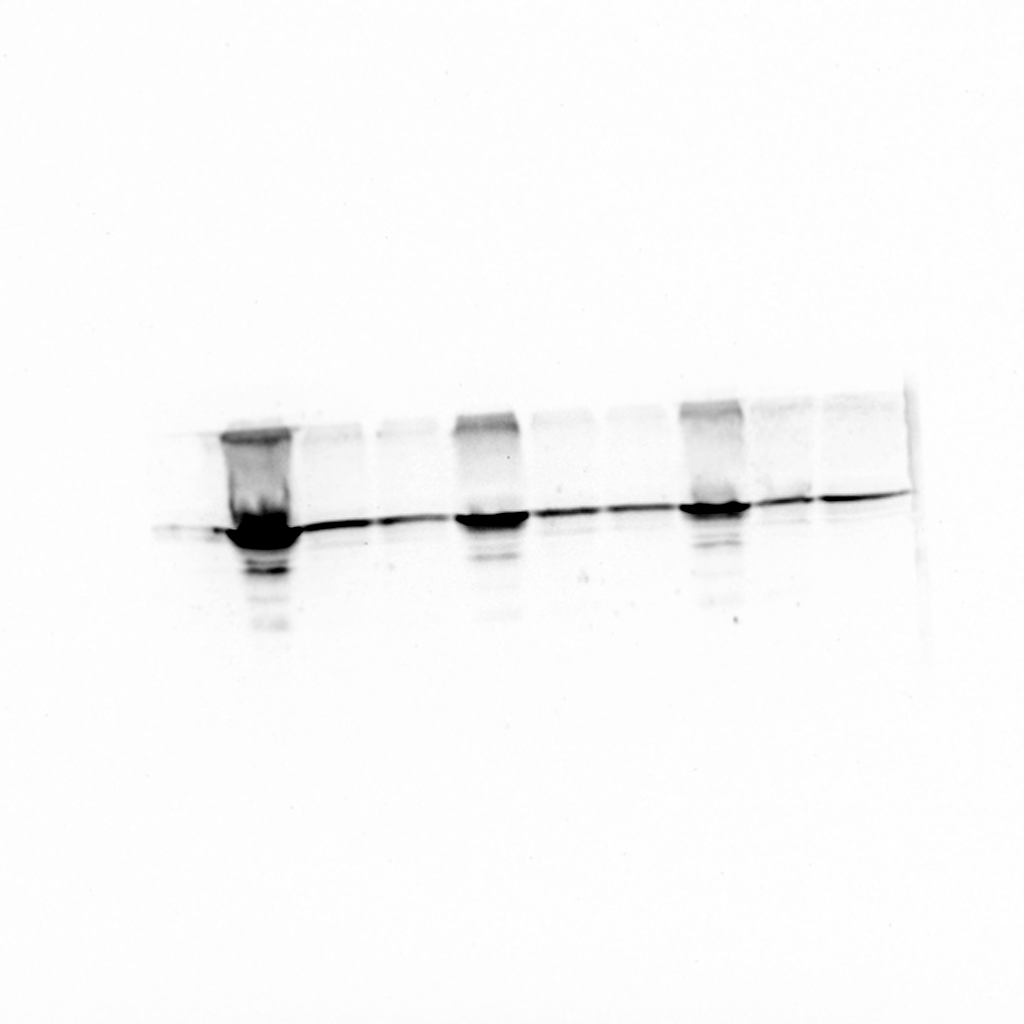

Supplement: Supplementary file 1 [file DataSheet_1.zip › Original source data - Western blot-Revised/Figure 6B/U251 Repeat-3 XIAP 5-7 lane.tif]

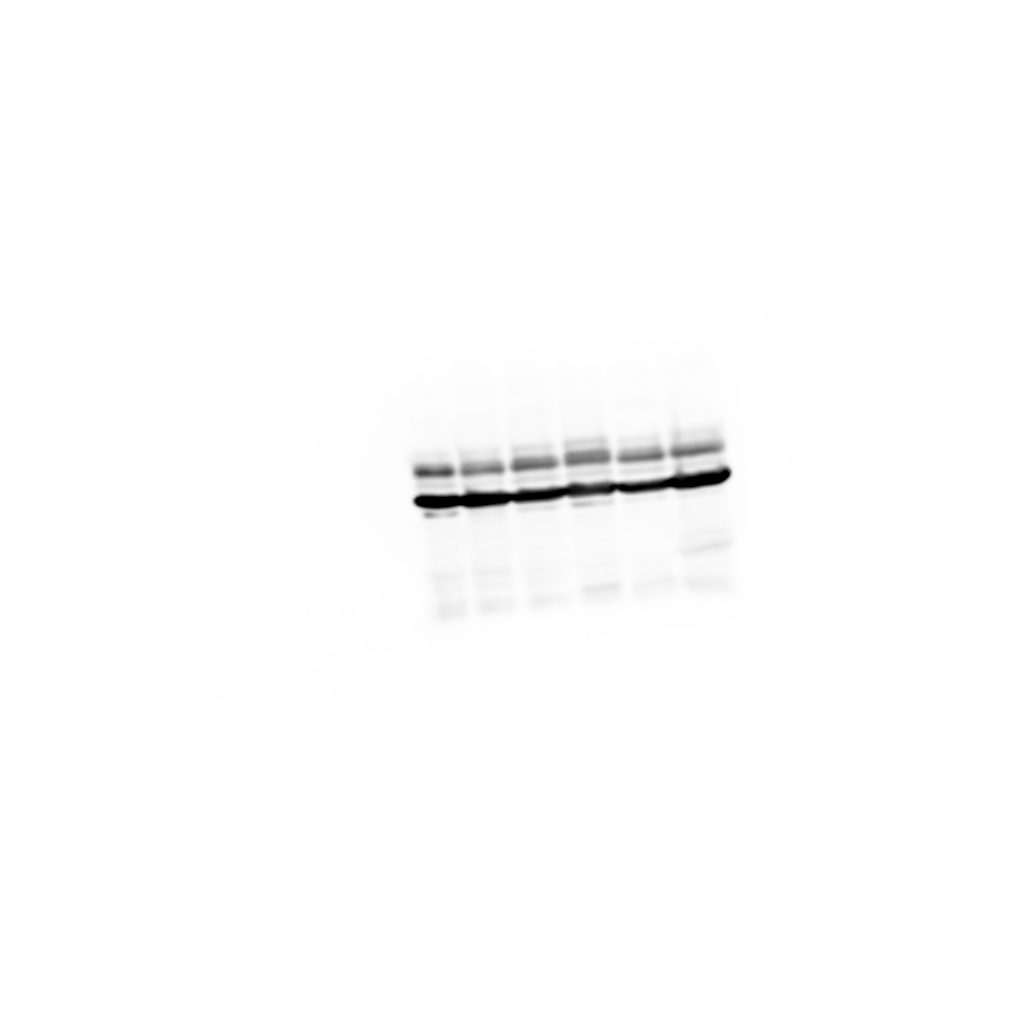

Supplement: Supplementary file 1 [file DataSheet_1.zip › Original source data - Western blot-Revised/Figure 6B/U87 Repeat-1 GAPDH 1-3 lane.tif]

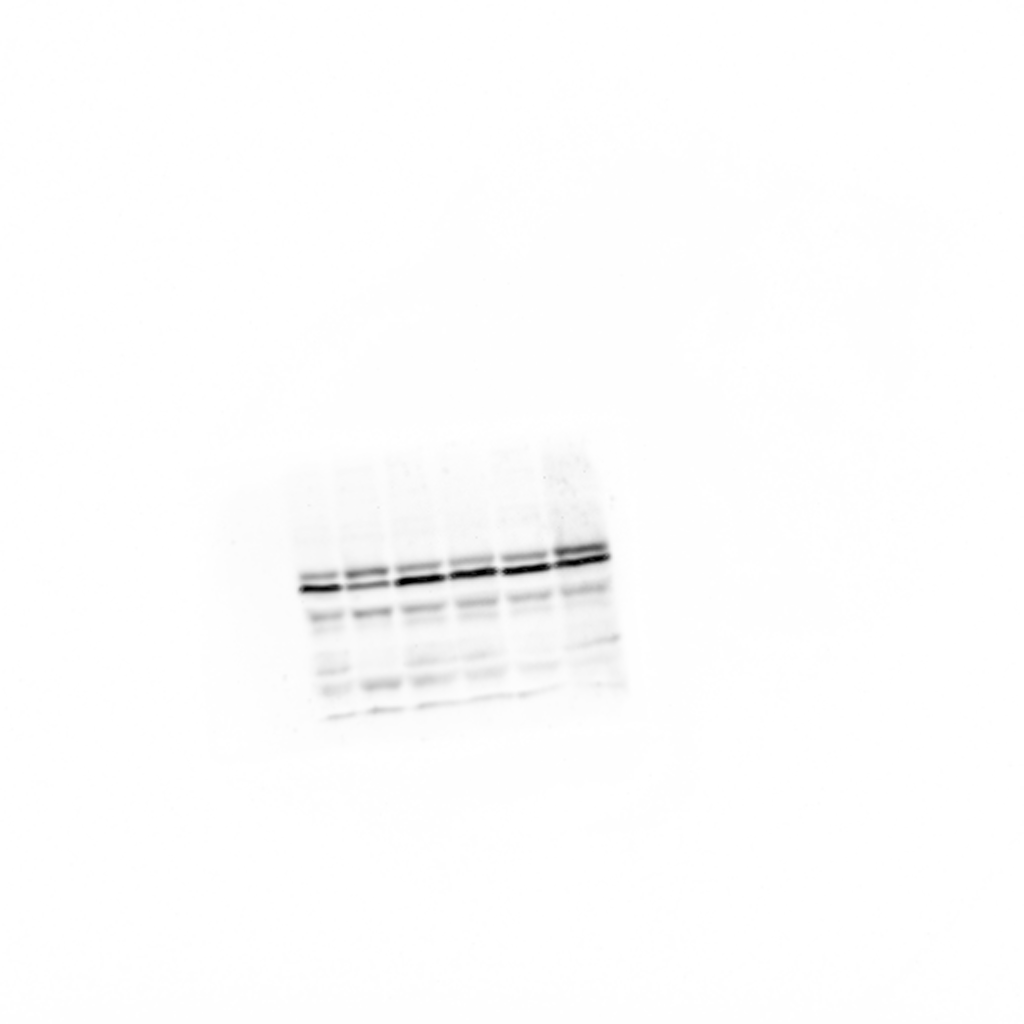

Supplement: Supplementary file 1 [file DataSheet_1.zip › Original source data - Western blot-Revised/Figure 6B/U87 Repeat-1 XIAP 1-3 lane.tif]

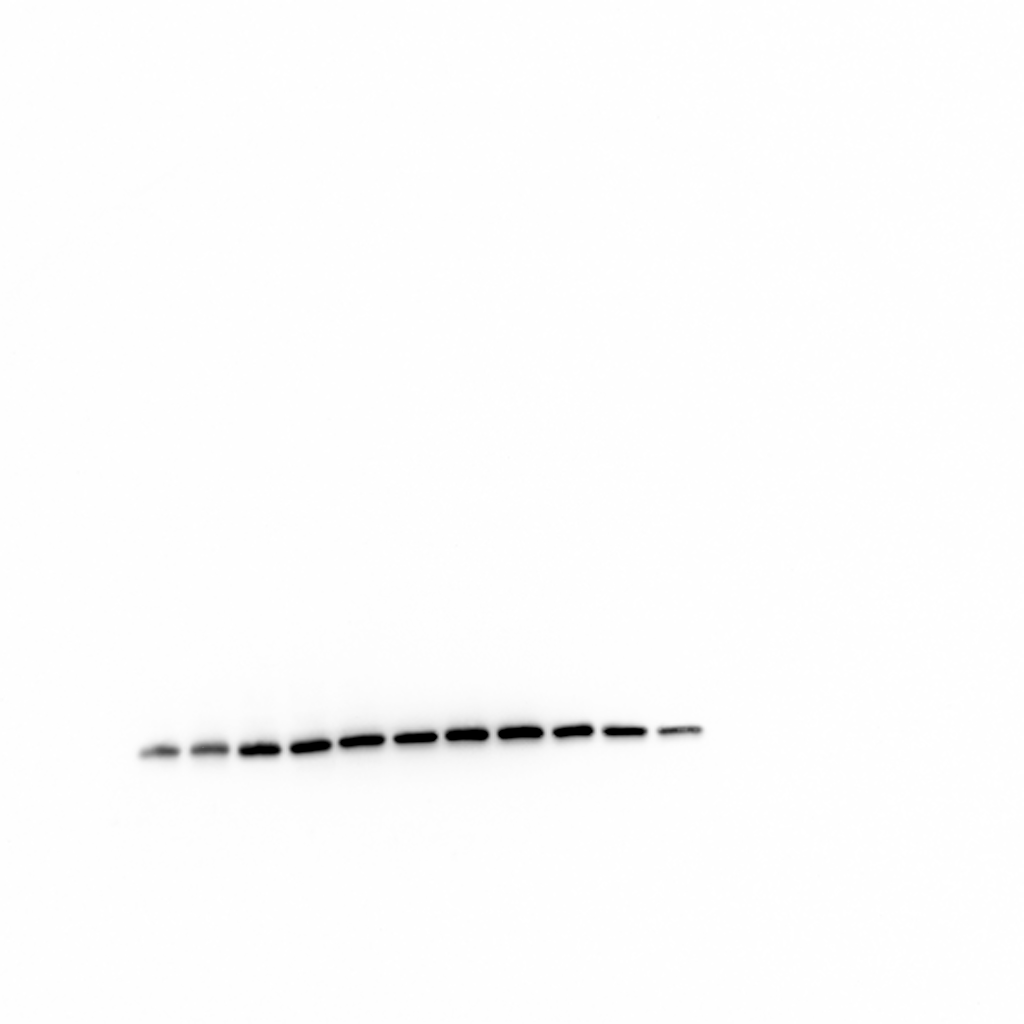

Supplement: Supplementary file 1 [file DataSheet_1.zip › Original source data - Western blot-Revised/Figure 6B/U87 Repeat-2 GAPDH 3-5 lane.tif]

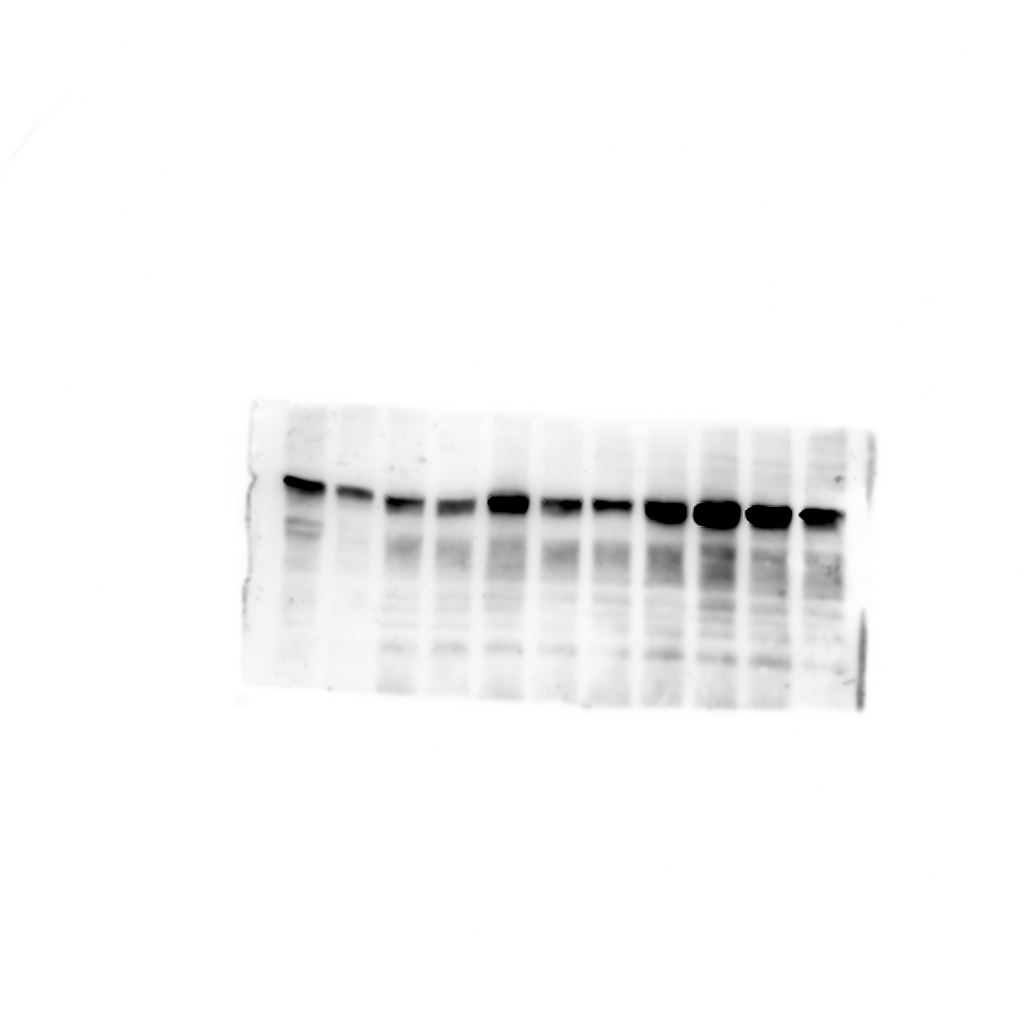

Supplement: Supplementary file 1 [file DataSheet_1.zip › Original source data - Western blot-Revised/Figure 6B/U87 Repeat-2 XIAP 3-5 lane.tif]

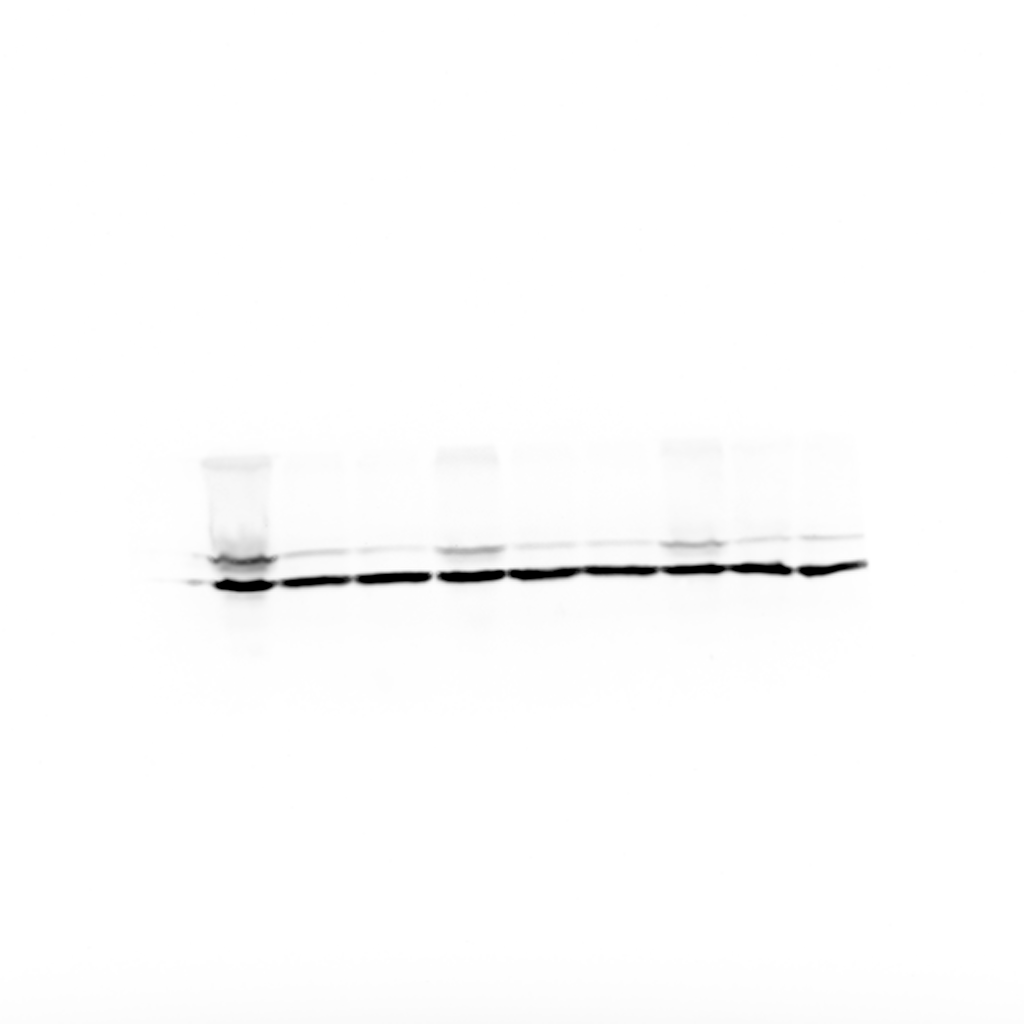

Supplement: Supplementary file 1 [file DataSheet_1.zip › Original source data - Western blot-Revised/Figure 6B/U87 Repeat-3 GAPDH 2-4 lane.tif]

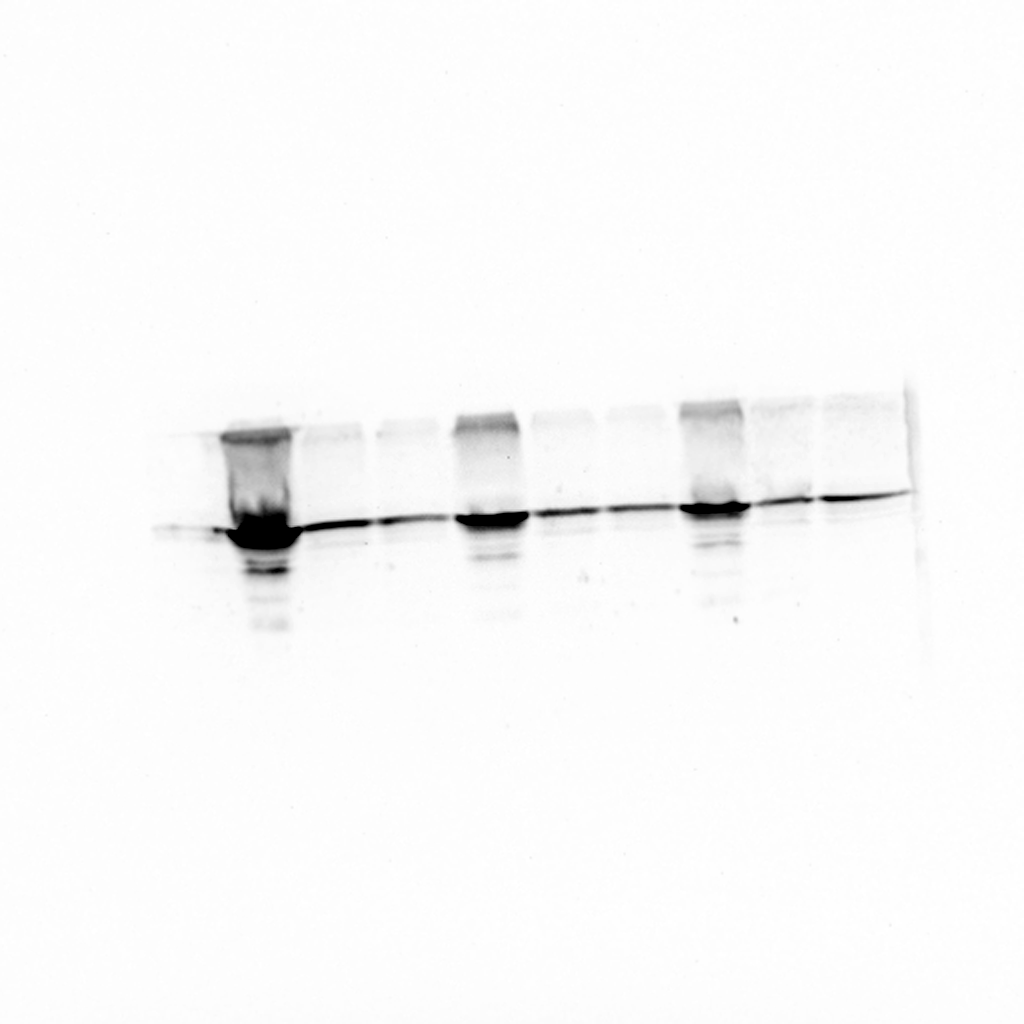

Supplement: Supplementary file 1 [file DataSheet_1.zip › Original source data - Western blot-Revised/Figure 6B/U87 Repeat-3 XIAP 2-4 lane.tif]

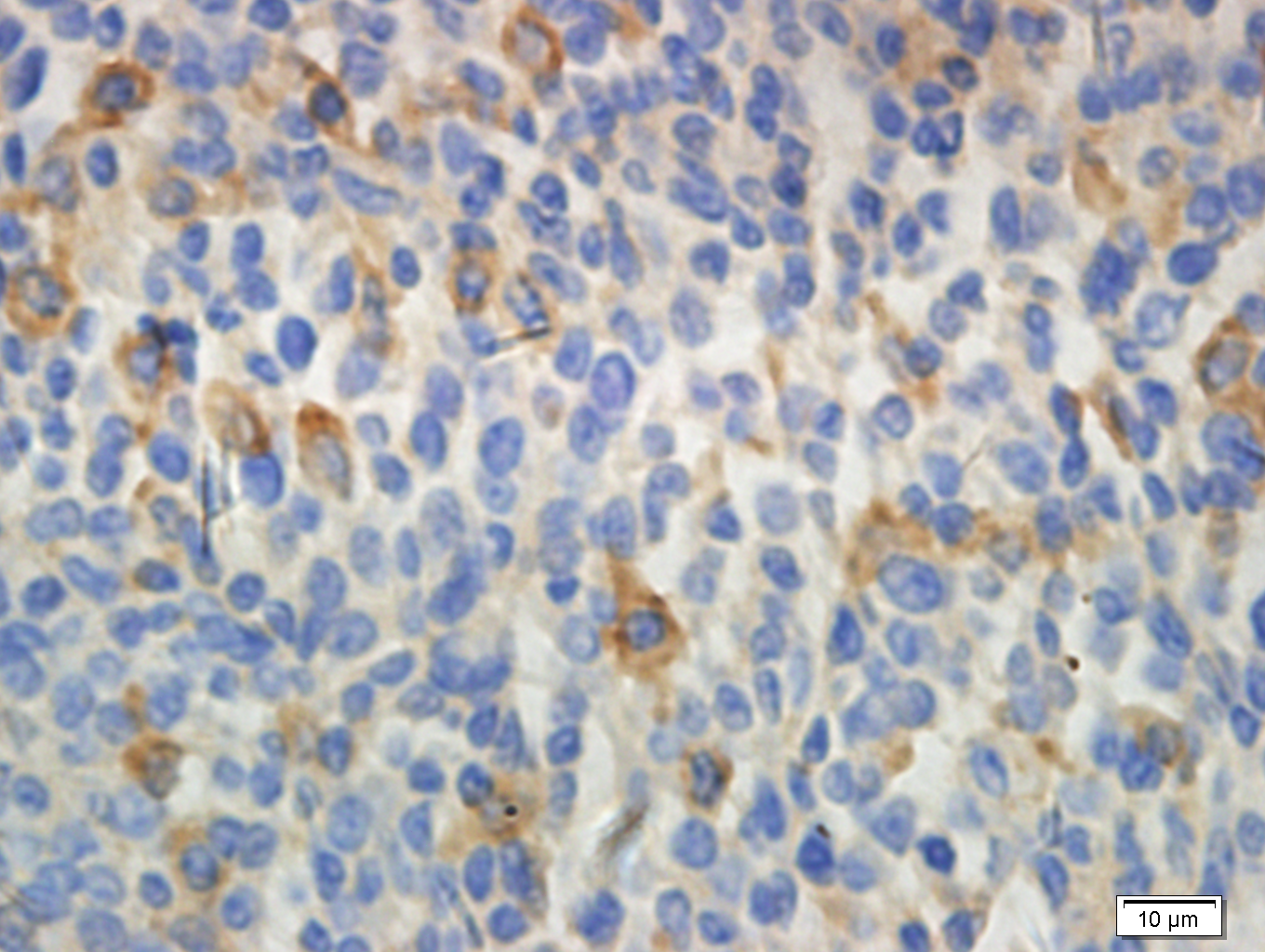

Supplement: Supplementary file 3 [file DataSheet_3.zip › Original source data - Microscopy images -1-Revised/Figure 7A/Luc 100X.tif]

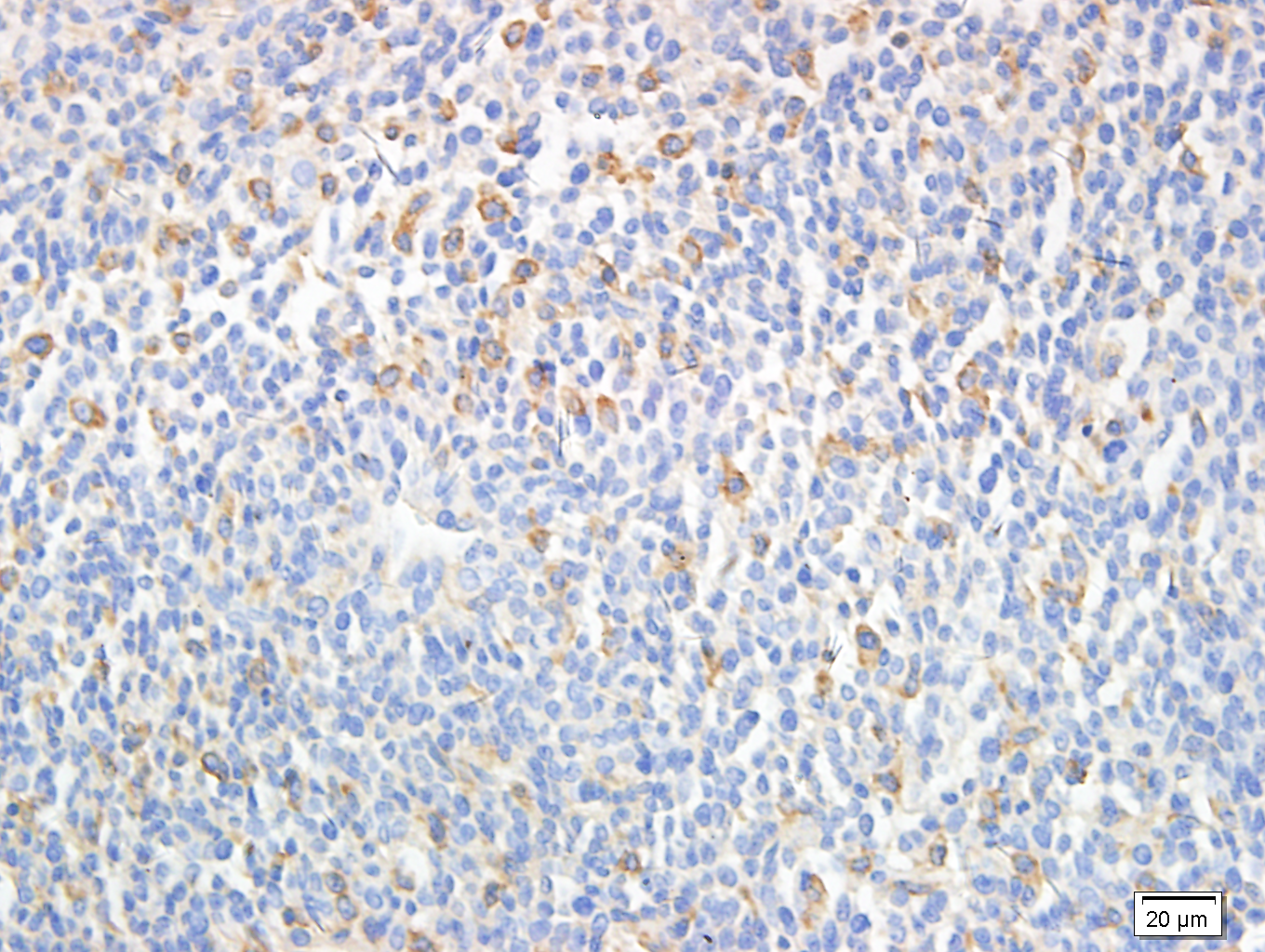

Supplement: Supplementary file 3 [file DataSheet_3.zip › Original source data - Microscopy images -1-Revised/Figure 7A/Luc 40X.tif]

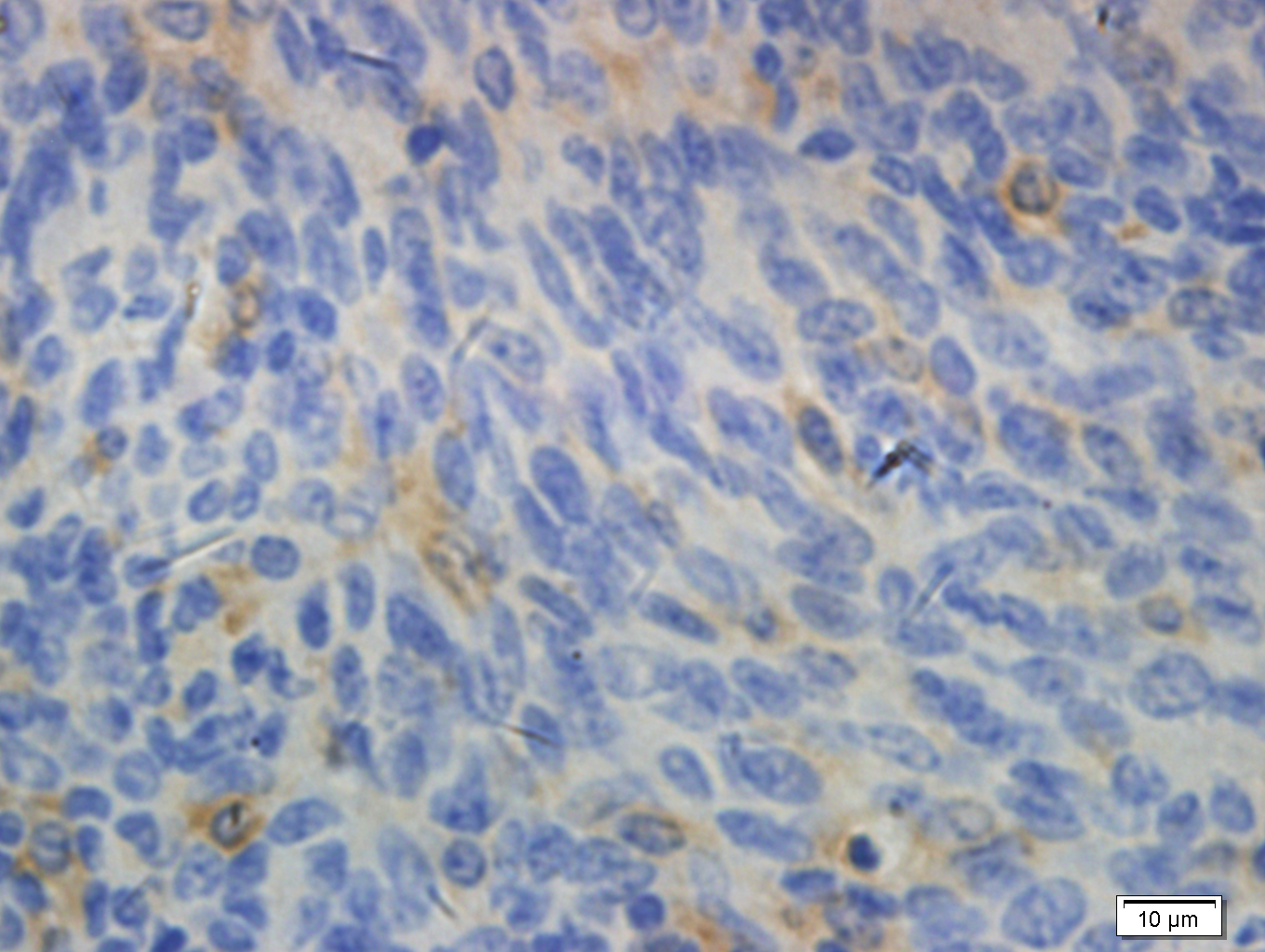

Supplement: Supplementary file 3 [file DataSheet_3.zip › Original source data - Microscopy images -1-Revised/Figure 7A/miR-137 100X.tif]

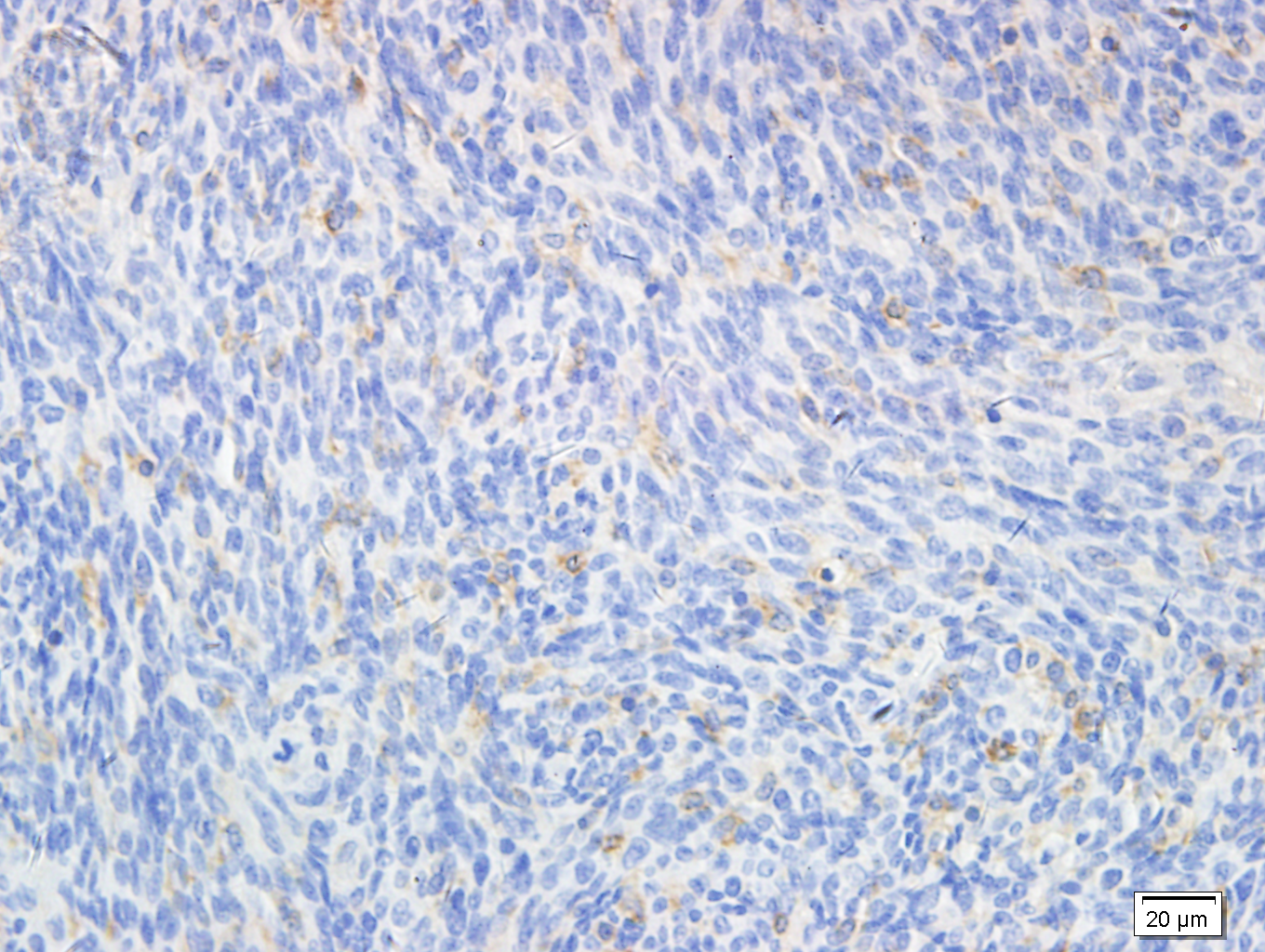

Supplement: Supplementary file 3 [file DataSheet_3.zip › Original source data - Microscopy images -1-Revised/Figure 7A/miR-137 40X.tif]

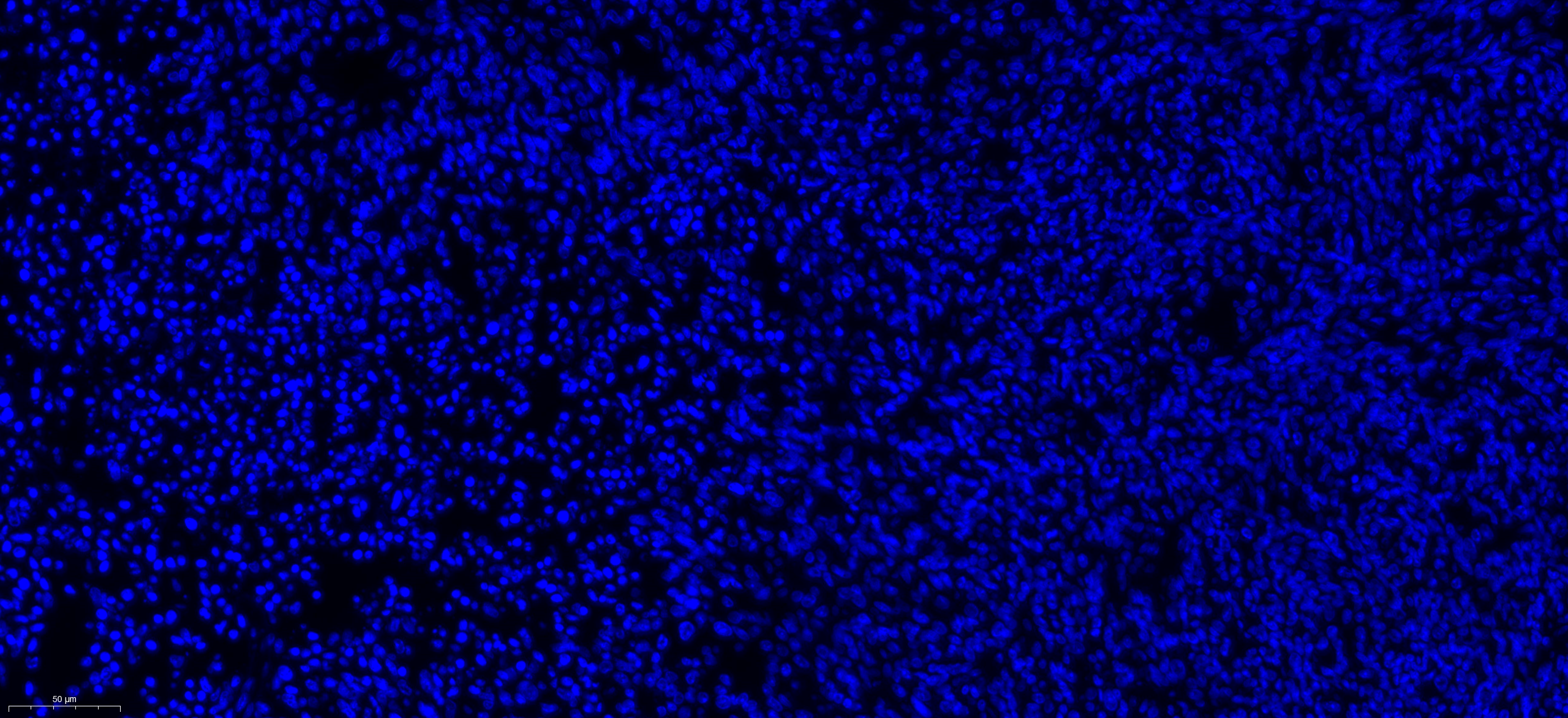

Supplement: Supplementary file 4 [file DataSheet_4.zip › Original source data - Microscopy images -2-Revised/Figure 7E/Luc 40x DAPI.tif]

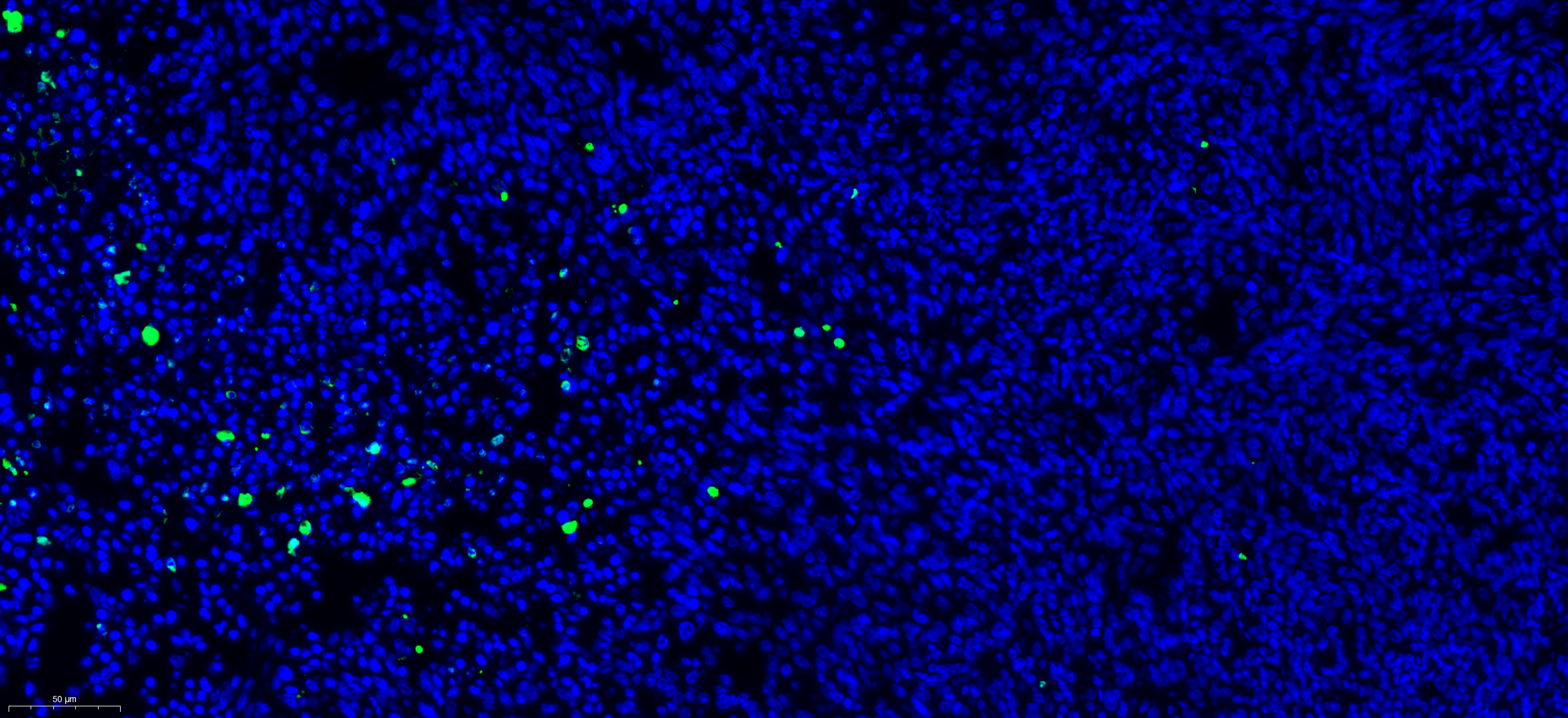

Supplement: Supplementary file 4 [file DataSheet_4.zip › Original source data - Microscopy images -2-Revised/Figure 7E/Luc 40x MERGE.tif]

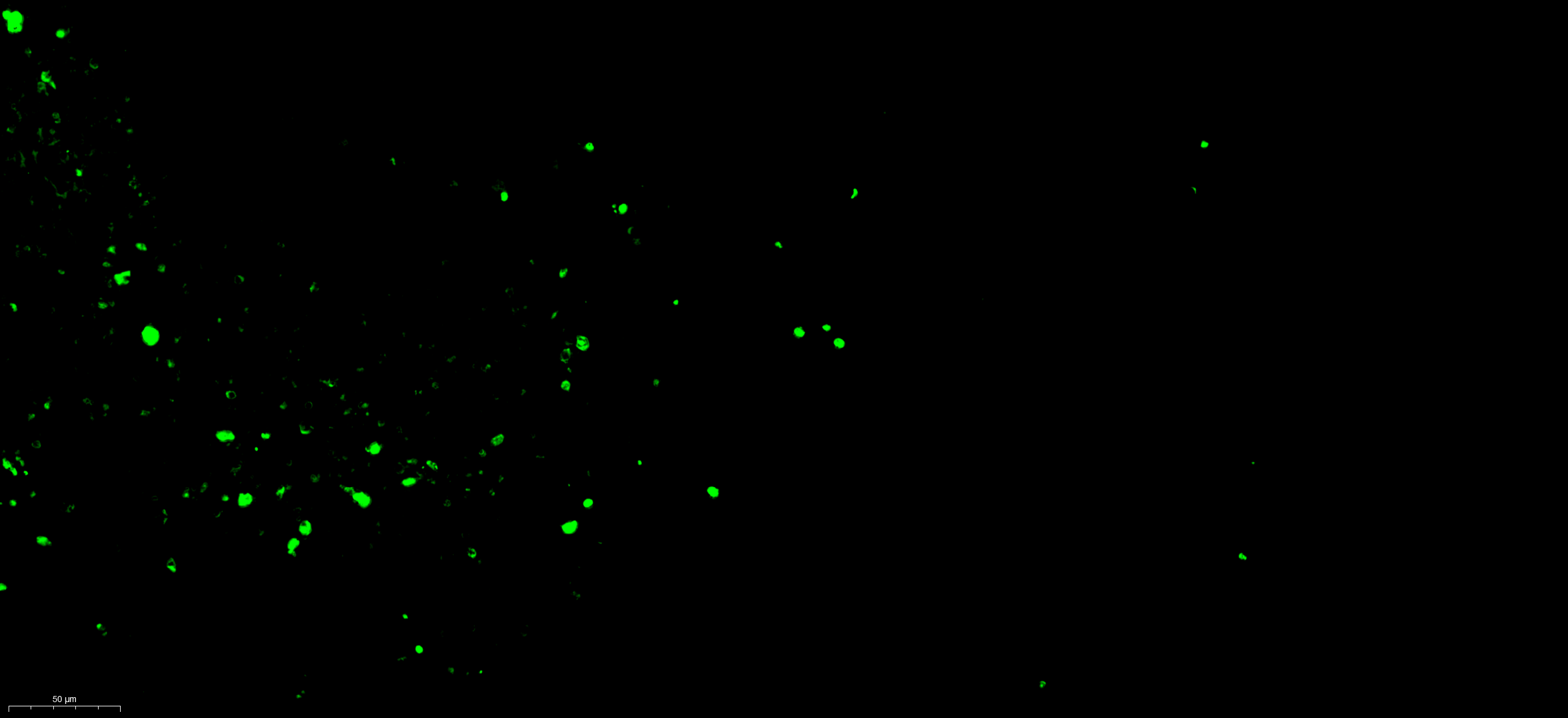

Supplement: Supplementary file 4 [file DataSheet_4.zip › Original source data - Microscopy images -2-Revised/Figure 7E/Luc 40x TUNEL.tif]

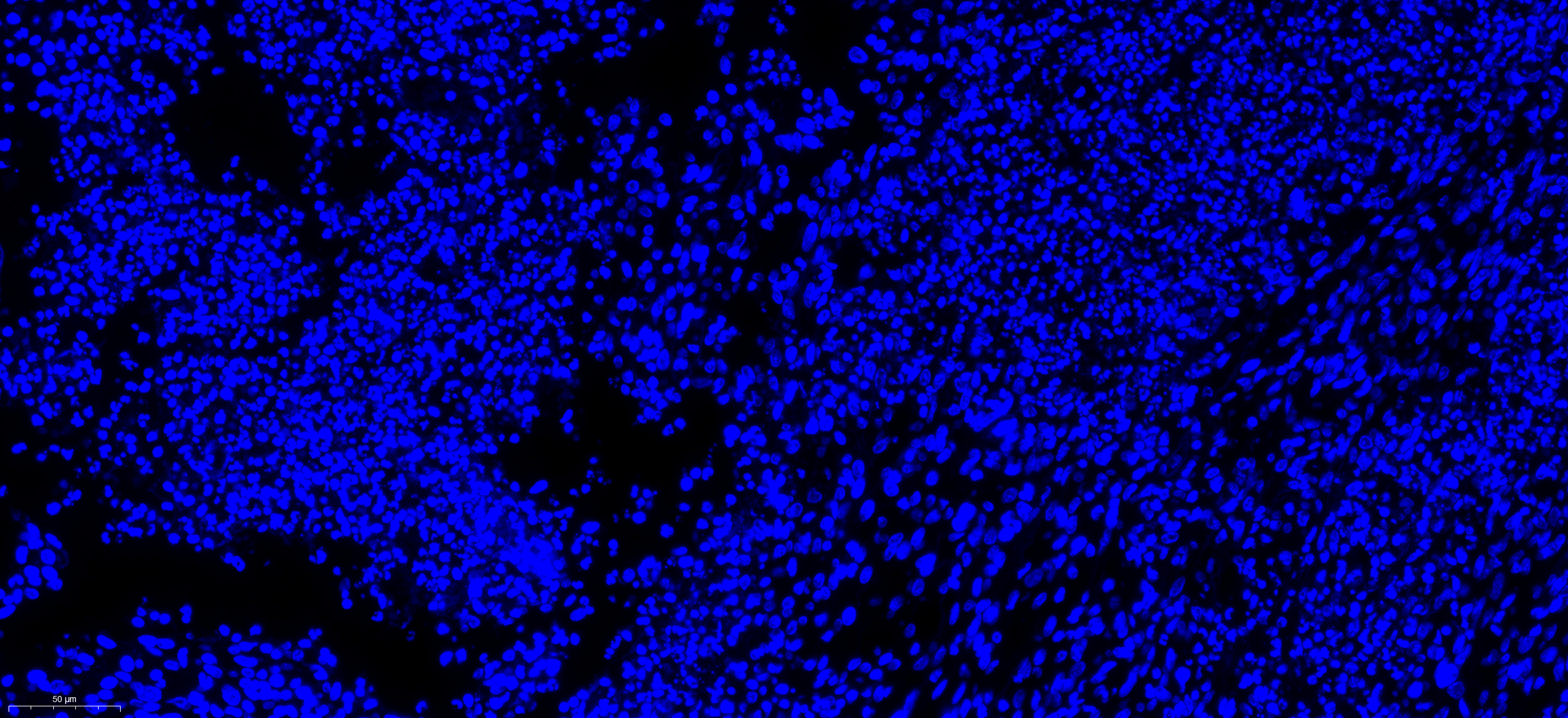

Supplement: Supplementary file 4 [file DataSheet_4.zip › Original source data - Microscopy images -2-Revised/Figure 7E/Luc+TRAIL 40x DAPI.tif]

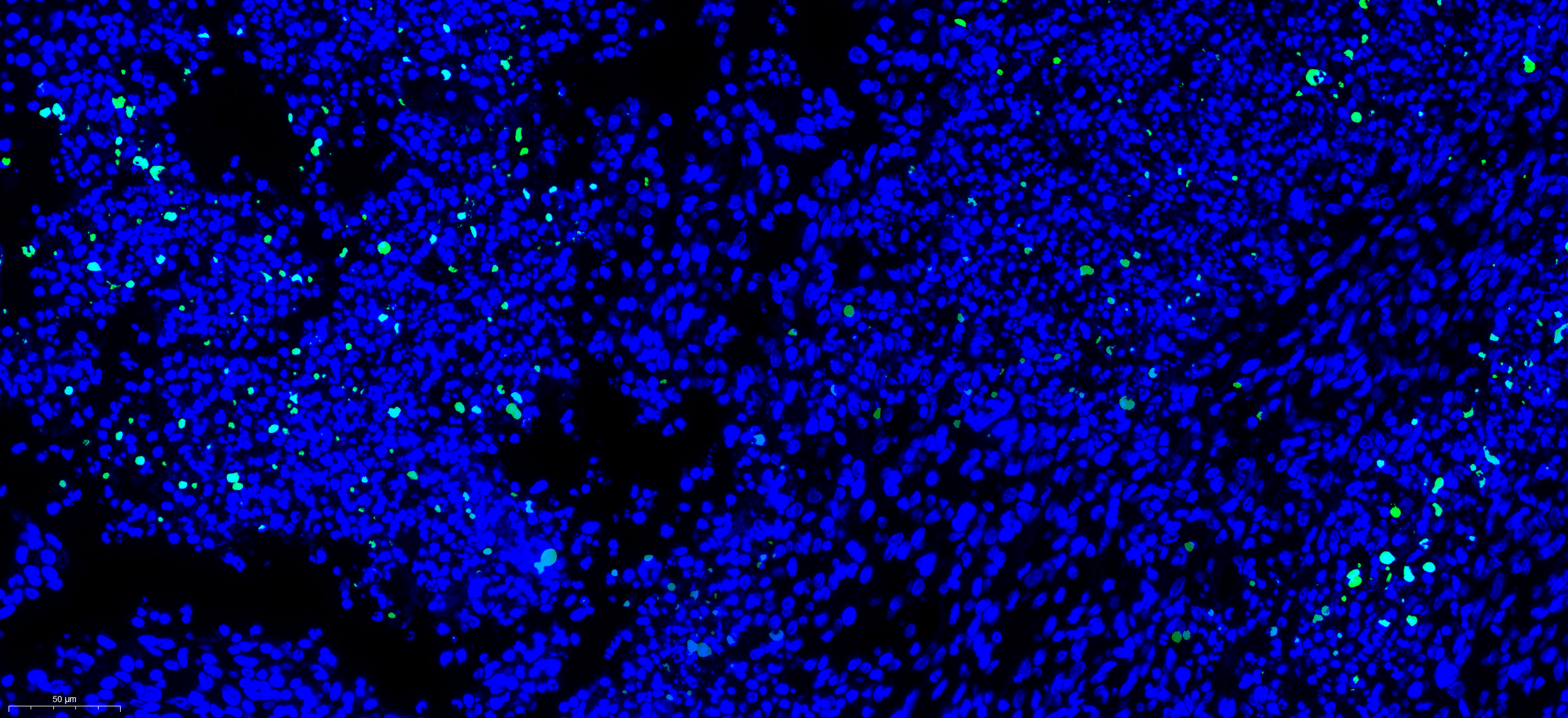

Supplement: Supplementary file 4 [file DataSheet_4.zip › Original source data - Microscopy images -2-Revised/Figure 7E/Luc+TRAIL 40x MERGE.tif]

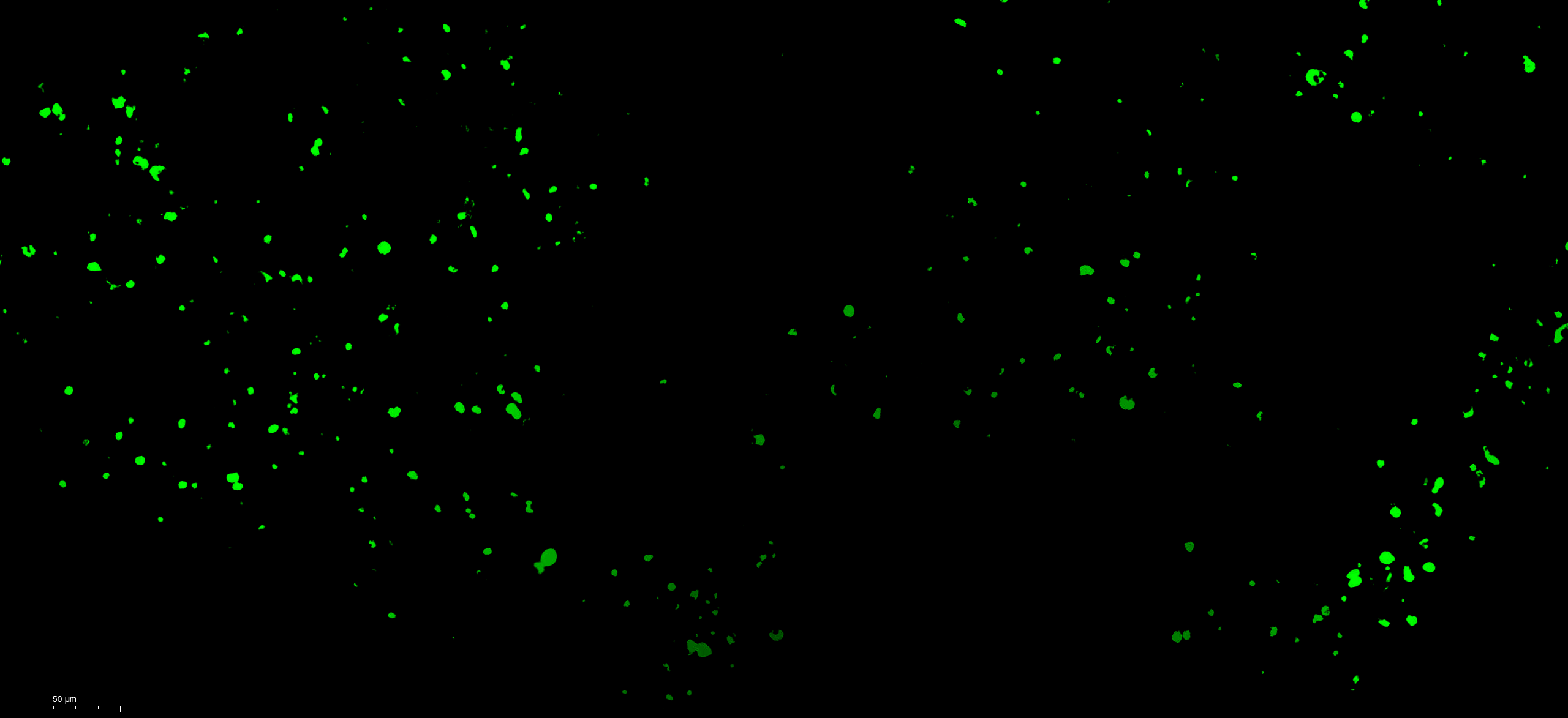

Supplement: Supplementary file 4 [file DataSheet_4.zip › Original source data - Microscopy images -2-Revised/Figure 7E/Luc+TRAIL 40x TUNEL.tif]

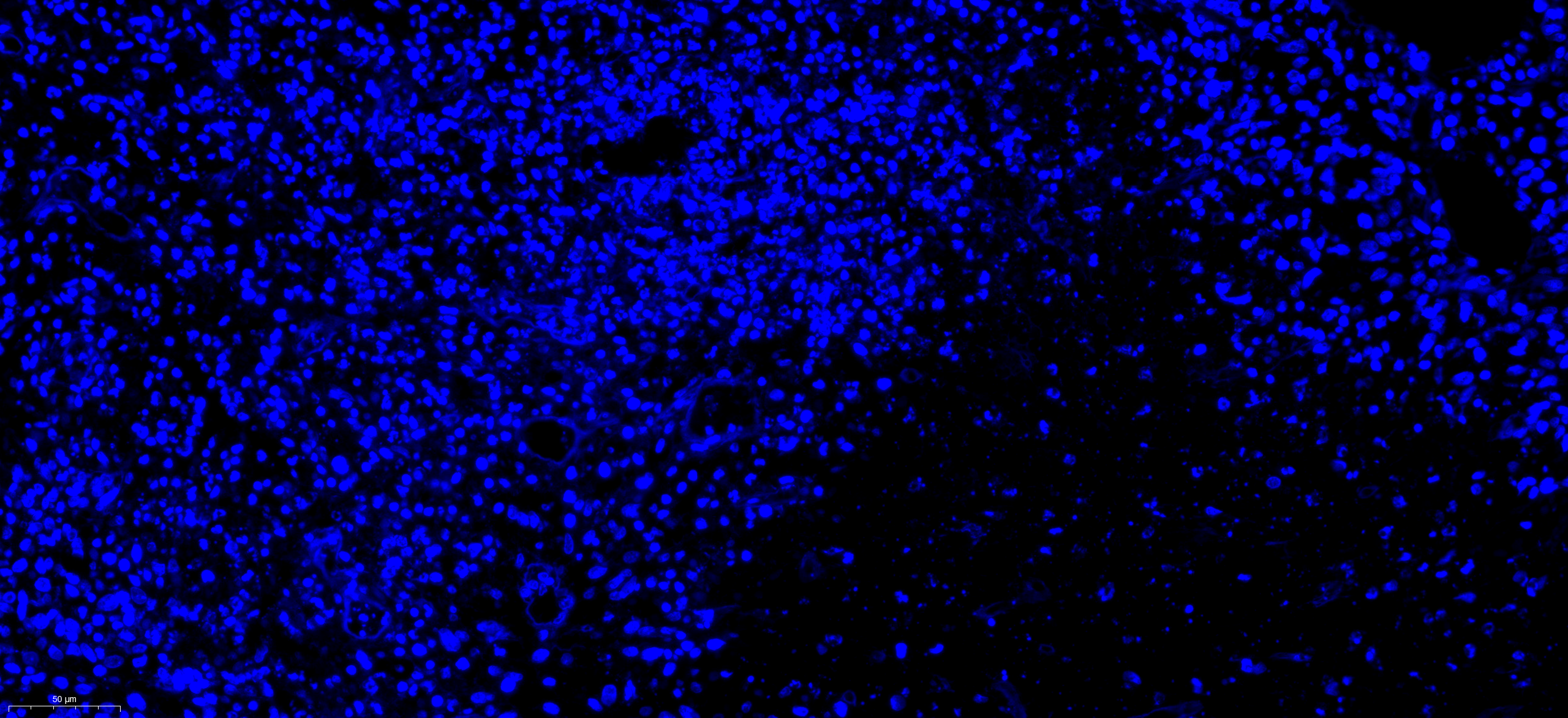

Supplement: Supplementary file 4 [file DataSheet_4.zip › Original source data - Microscopy images -2-Revised/Figure 7E/miR-137 40x DAPI.tif]

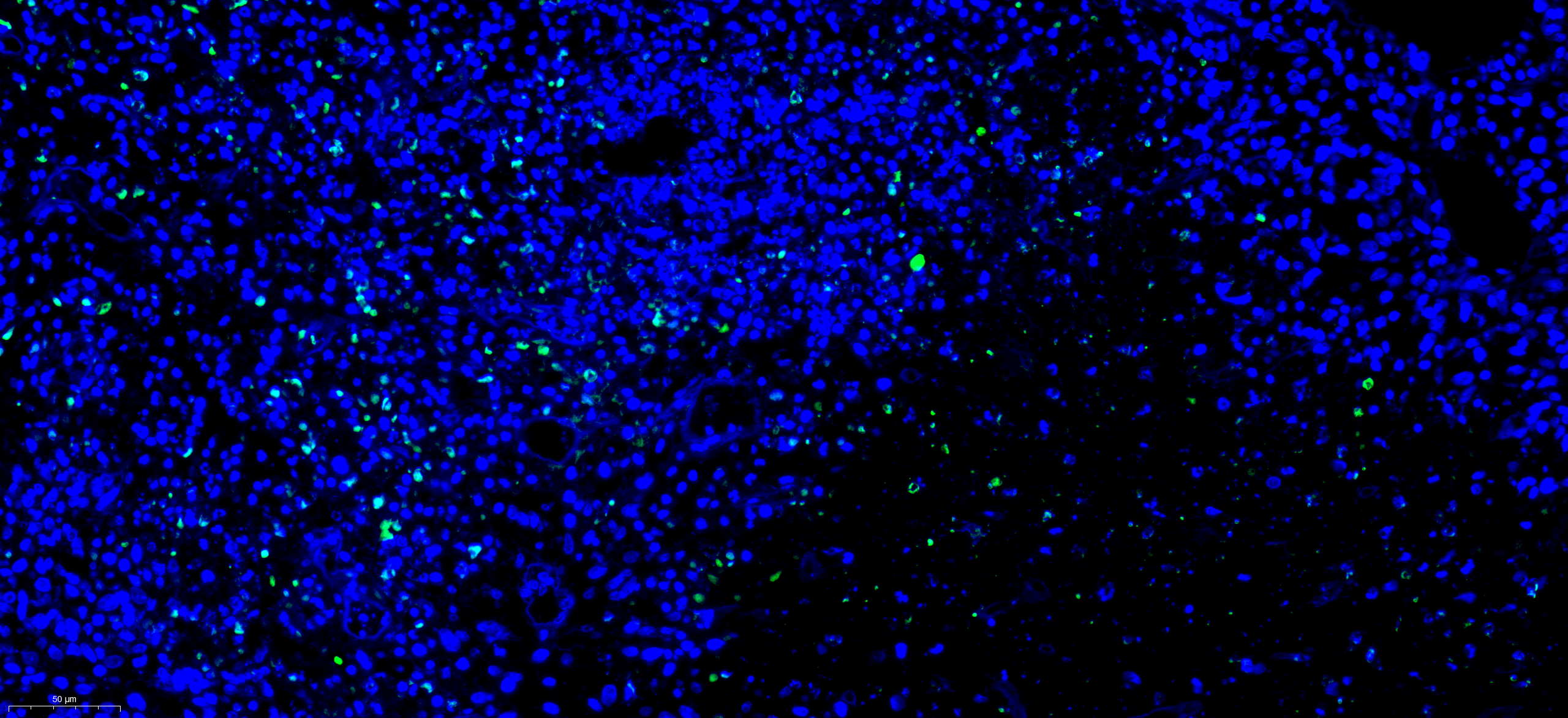

Supplement: Supplementary file 4 [file DataSheet_4.zip › Original source data - Microscopy images -2-Revised/Figure 7E/miR-137 40x MERGE.tif]

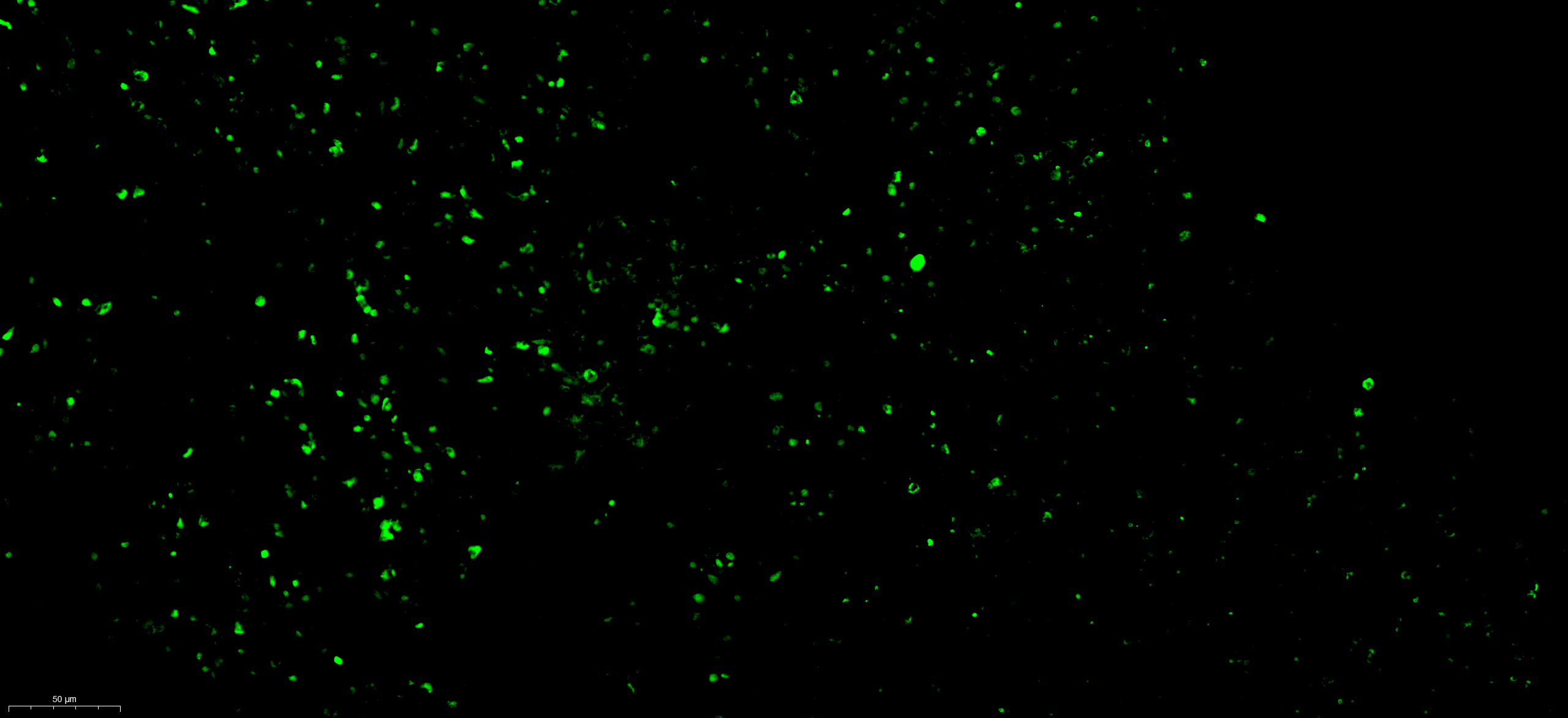

Supplement: Supplementary file 4 [file DataSheet_4.zip › Original source data - Microscopy images -2-Revised/Figure 7E/miR-137 40x TUNEL.tif]

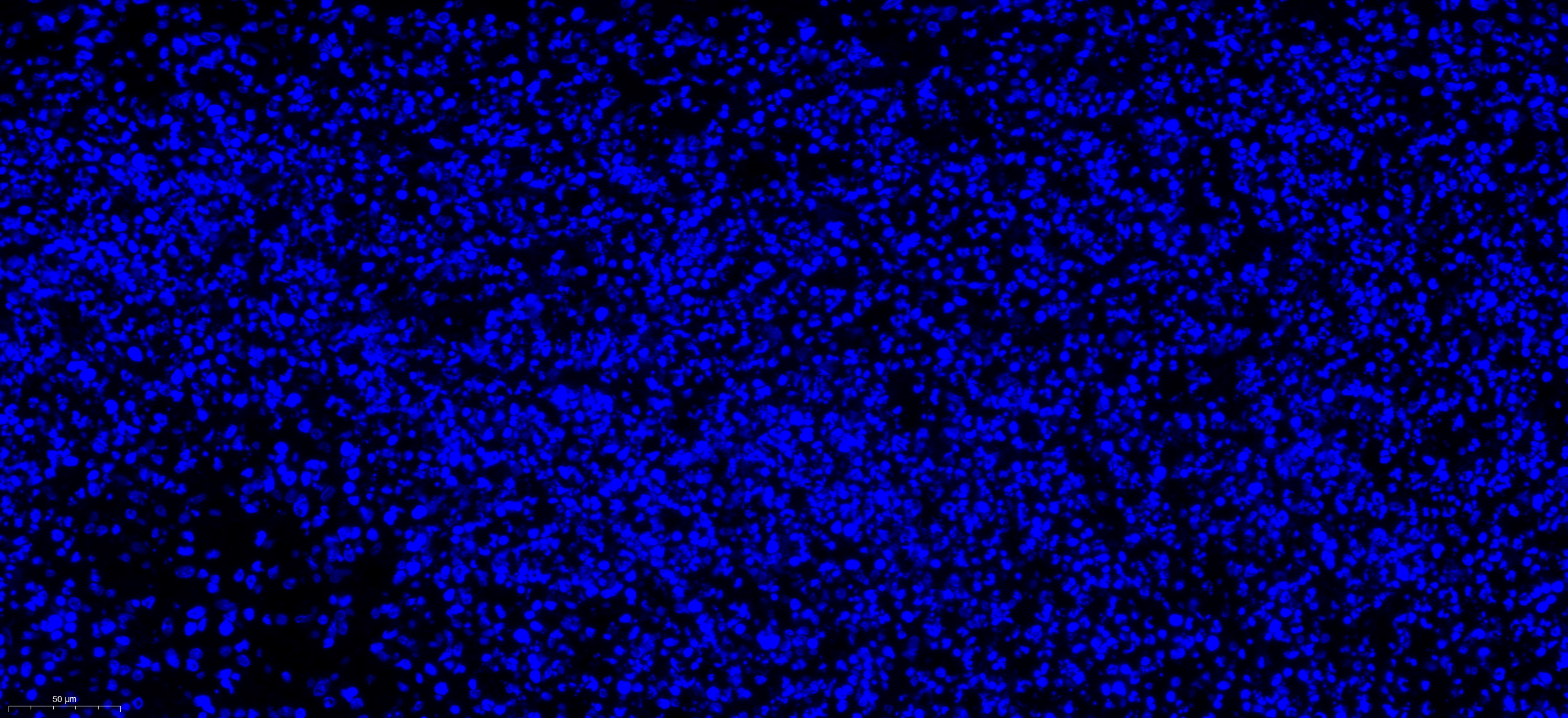

Supplement: Supplementary file 4 [file DataSheet_4.zip › Original source data - Microscopy images -2-Revised/Figure 7E/miR-137+TRAIL 40x DAPI.jpg]

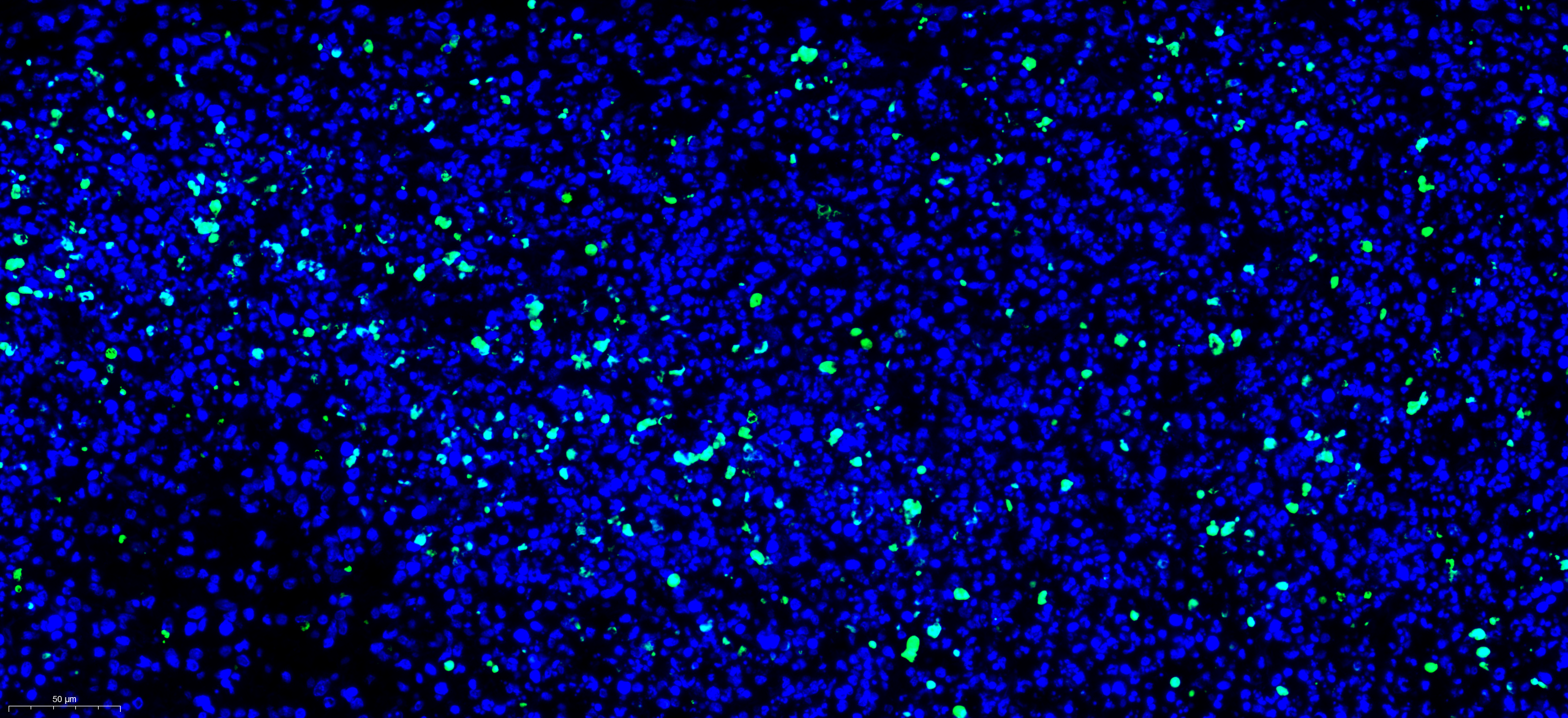

Supplement: Supplementary file 4 [file DataSheet_4.zip › Original source data - Microscopy images -2-Revised/Figure 7E/miR-137+TRAIL 40x MERGE.tif]

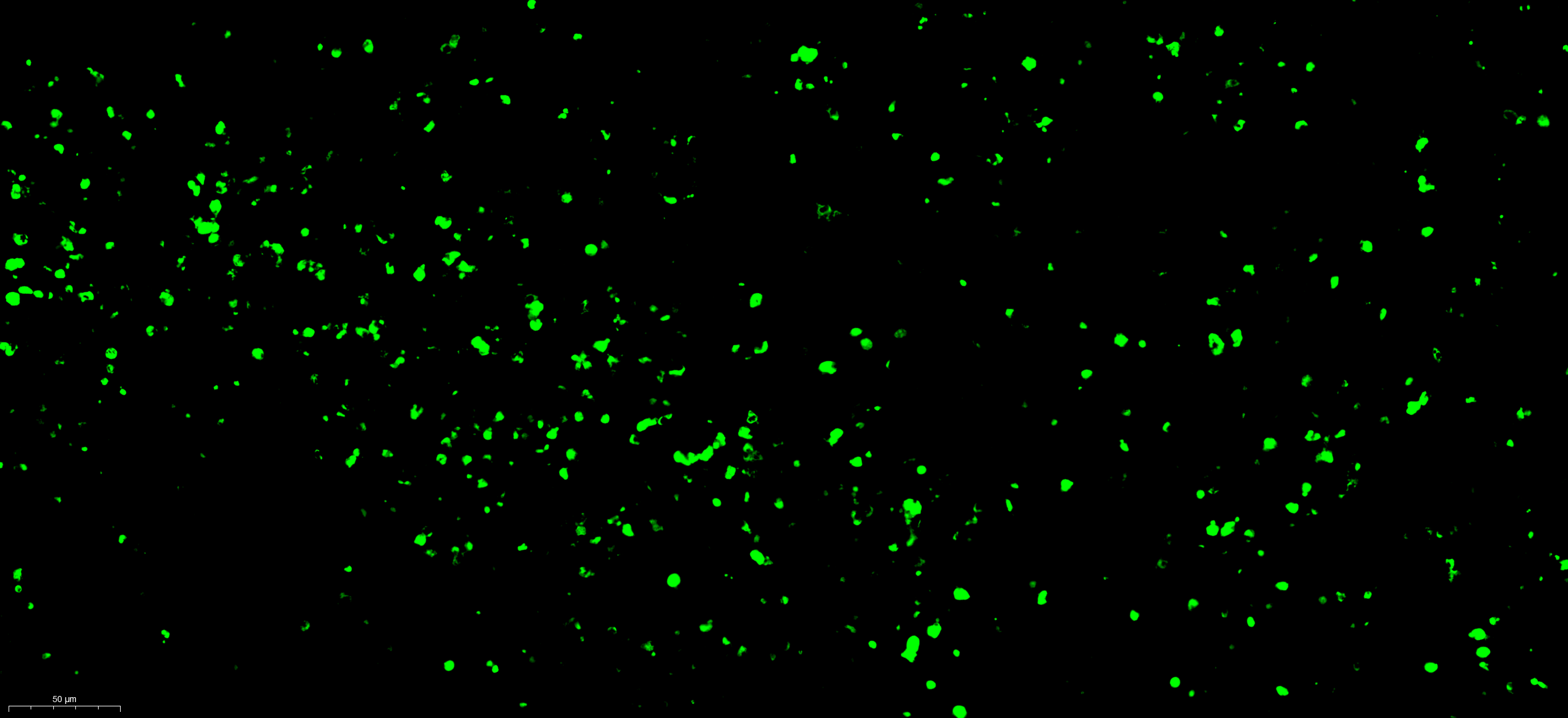

Supplement: Supplementary file 4 [file DataSheet_4.zip › Original source data - Microscopy images -2-Revised/Figure 7E/miR-137+TRAIL 40x TUNEL.tif]
