# Supplementary material for: A miR-137-XIAP axis contributes to the sensitivity of TRAIL-induced cell death in glioblastoma
Source: Front Oncol. 2022 Jul 28;12:870034. doi: 10.3389/fonc.2022.870034 (PMC9366219; doi:10.3389/fonc.2022.870034)
Supplement: Supplementary file 5 [file Table_1.docx]

**Table S1 Primers used for qRT-PCR.**

| **qRT-PCR primers** | **Sequences (5’-3’)** |
| --- | --- |
| **BAG4** | Forward: AATGGAGCGTATGGTCCAACA |
|  | Reverse: GGTGCATAATAAGCCCCTGAGT |
| **AKT2** | Forward: ACCACAGTCATCGAGAGGACC |
|  | Reverse: GGAGCCACACTTGTAGTCCA |
| **BCL2L11** | Forward: TAAGTTCTGAGTGTGACCGAGA |
|  | Reverse: GCTCTGTCTGTAGGGAGGTAGG |
| **JUN** | Forward: TCCAAGTGCCGAAAAAGGAAG |
|  | Reverse: CGAGTTCTGAGCTTTCAAGGT |
| **CASP3** | Forward: CATGGAAGCGAATCAATGGACT |
|  | Reverse: CTGTACCAGACCGAGATGTCA |
| **CREM** | Forward: ACAGTACGCAGCACAATCAG |
|  | Reverse: CTGGTAAGTTGGCATGTCACC |
| **JDP2** | Forward: CCCAGCCCGTGAAAAGTGA |
|  | Reverse: CGGTGTCGGTTCAGCATCA |
| **MAP3K1** | Forward: CATCAGGTCGCACAGTGAAAT |
|  | Reverse: TCAGGGCTATATGGTGAGAAGC |
| **MAP2K4** | Forward: TCCCAATCCTACAGGAGTTCAA |
|  | Reverse: CCAGTGTTGTTCAGGGGAGA |
| **MAP4K2** | Forward: GGCAGCTACCTCAGGAATGAC |
|  | Reverse: CCAGTGGCATGGTAAATCTCC |
| **MAP4K5** | Forward: CCCCATGCAATCATTCGTCAT |
|  | Reverse: CCCATTTCATCTCGTGCTTCTG |
